# Multikinase inhibitors modulate non-constitutive proteasome expression in colorectal cancer cells

**DOI:** 10.3389/fmolb.2024.1351641

**Published:** 2024-05-07

**Authors:** Alexander Burov, Ekaterina Grigorieva, Timofey Lebedev, Valeria Vedernikova, Vladimir Popenko, Tatiana Astakhova, Olga Leonova, Pavel Spirin, Vladimir Prassolov, Vadim Karpov, Alexey Morozov

**Affiliations:** ^1^ Laboratory of Regulation of Intracellular Proteolysis, Engelhardt Institute of Molecular Biology, Russian Academy of Sciences, Moscow, Russia; ^2^ Moscow Institute of Physics and Technology, National Research University, Dolgoprudny, Russia; ^3^ Department of Cancer Cell Biology, Engelhardt Institute of Molecular Biology, Russian Academy of Sciences, Moscow, Russia; ^4^ Laboratory of Biochemistry of Ontogenesis Processes, Koltzov Institute of Developmental Biology, Russian Academy of Sciences, Moscow, Russia

**Keywords:** ubiquitin-proteasome system, non-constitutive proteasomes, multikinase inhibitors, regorafenib, sorafenib

## Abstract

**Introduction:** Proteasomes are multi-subunit protein complexes responsible for protein degradation in cells. Immunoproteasomes and intermediate proteasomes (together non-constitutive proteasomes) are specific forms of proteasomes frequently associated with immune response, antigen presentation, inflammation and stress. Expression of non-constitutive proteasome subunits has a prognostic value in several types of cancer. Thus, factors that modulate non-constitutive proteasome expression in tumors are of particular interest. Multikinase inhibitors (MKIs) demonstrate promising results in treatment of cancer. At the same time, their immunomodulatory properties and effects on non-constitutive proteasome expression in colorectal cancer cells are poorly investigated.

**Methods:** Proteasome subunit expression in colorectal cancer was evaluated by bioinformatic analysis of available datasets. Two colorectal cancer cell lines, expressing fluorescent non-constitutive proteasomes were treated with multikinase inhibitors: regorafenib and sorafenib. The proteasome subunit expression was assessed by real-time PCR, Western blotting and flow cytometry. The proteasome activity was studied using proteasome activity-based probe and fluorescent substrates. Intracellular proteasome localization was revealed by confocal microscopy. Reactive oxygen species levels following treatment were determined in cells. Combined effect of proteasome inhibition and treatment with MKIs on viability of cells was estimated.

**Results:** Expression of non-constitutive proteasomes is increased in BRAF-mutant colorectal tumors. Regorafenib and sorafenib stimulated the activity and synthesis of non-constitutive proteasomes in examined cell lines. MKIs induced oxidative stress and redistribution of proteasomes within cells. Sorafenib stimulated formation of cytoplasmic aggregates, containing proteolyticaly active non-constitutive proteasomes, while regorafenib had no such effect. MKIs caused no synergistic action when were combined with the proteasome inhibitor.

**Discussion:** Obtained results indicate that MKIs might affect the crosstalk between cancer cells and immune cells via modulation of intracellular proteasome pool. Observed phenomenon should be considered when MKI-based therapy is applied.

## 1 Introduction

Cancer cells are highly dependent on the proper functioning of the ubiquitin-proteasome system (UPS) which supports homeostasis via degradation of most cellular proteins (Tsvetkov et al., 2018). The UPS provide a cascade of reactions leading to the post-translational modification of the substrate protein with a small protein–ubiquitin. This tag is recognized by the multisubunit protein complex, known as the 26S proteasome, where the protein degradation takes place (Ciechanover and Scwartz, 1998). The 26S proteasome consists of the 19S regulator/s which specifically recognizes the ubiquitinated substrates and the 20S proteasome that contains proteolytic centers (reviewed in (Morozov and Karpov, 2019)). With no attached 19S regulator/s the 20S proteasome is incapable to selectively degrade ubiqitinated proteins, but still can break down certain substrates including oxidized and damaged proteins (Kumar Deshmukh et al., 2019). Within the constitutive 20S proteasome, three subunits perform proteolysis and cleave peptide bonds after acidic (subunit β1), basic (subunit β2) and hydrophobic (subunit β5) amino acids. These subunits can be substituted by analogs (β1i, β2i and β5i), known as the immune subunits during the proteasome assembly, leading to the formation of the immunoproteasome or an intermediate proteasome, if not all constitutive catalytic subunits are replaced (Guillaume et al., 2010). These proteasomes, together non-constitutive proteasomes, demonstrate altered activity profile and thus, generate altered sets of peptides, which are further presented on the cell surface by the MHCI molecules (Winter et al., 2017). Consequently, along with other functions, these proteasomes facilitate antigen presentation; mice lacking immunoproteasomes display 50% different repertoire of presented peptides and altered response to viral infection (Kincaid et al., 2011). Non-constitutive proteasomes are abundant in the immune cells. In somatic cells the quantity of these proteasomes may rise drastically in conditions of stress, inflammation or following stimulation with pro-inflammatory cytokines including IFN-γ and TNF-α (Aki et al., 1994).

UPS-directed approach for cancer treatment includes mostly utilization of proteasome inhibitors or their combinations with other drugs. Indeed, bortezomib (Velcade), carfilzomib (Kyprolis) and ixazomib (Ninlaro) targeting both constitutive and non-constitutive proteasomes are effective against multiple myeloma and mantle cell lymphoma, but against solid tumors their efficacy is limited (Astakhova et al., 2018; Roeten et al., 2018). Interestingly, increased expression of immunoproteasome subunits in cancer cells has a prognostic value for several different types of solid tumors (Rouette et al., 2016; Tripathi et al., 2016; Lee et al., 2019). Though increased levels of immunoproteasome subunits in cancers could be associated with immune cells infiltration, modulation of non-constitutive proteasome subunit expression and activity in cancer cells might affect the tumor-immune system interactions and consequently the outcome of the disease. Recently, inhibitors directed specifically to immune proteasome subunits were developed and their derivatives are now being evaluated in clinical trials (Huber and Groll, 2021). Except use of pro-inflammatory cytokines with pleiotropic effects, currently, no specific drugs that facilitate immunoproteasome synthesis are known. At the same time, several reports indicate altered immunoproteasome subunit expression following treatment of cancer cells with other types of anti-cancer drugs - protein kinase inhibitors that are currently widely used in clinical practice (Burov et al., 2021; Takahashi et al., 2021).

Here, we specifically addressed the effect of two multikinase inhibitors (MKIs) on the proteasome activity, expression and intracellular localization of non-constitutive proteasomes using two genetically modified colorectal cancer cell lines, engineered to express fluorescently labeled non-constitutive proteasome subunit β5i.

## 2 Materials and methods

### 2.1 Bioinformatics analysis of publicly available datasets

The dataset E-MTAB-10089 (Rohr et al., 2021) was used to compare gene expression in normal tissue, adenoma and colorectal cancer. One-way ANOVA test was used to determine significant differences with Benjamini-Hochberg correction for multiple comparisons. The GSE39582 dataset (Marisa et al., 2013) was used for Kaplan-Meier analysis, comparison of gene expression in molecular subtypes of colon cancer and calculation of Spearman correlation between *CD274* and *PSMB1-10* genes expression. For each molecular subtype samples from GSE39582 were divided into mutant/deficient and wild type/proficient categories and compared using Mann-Whitney non-parametric test with Benjamini-Hochberg correction for comparisons of multiple genes. Proteomic dataset for HCT-116 cells treated with small molecule inhibitors (Mitchell et al., 2023) was used to identify drugs which can affect proteasome levels in colorectal cancer cells (Supplementary Table S1). All data was downloaded from R2: Genomics Analysis and Visualization Platform.^1^ Kaplan-Meier analysis was performed using online tool from R2: Genomics Analysis and Visualization Platform. Correlations analyses, ANOVA tests and Benjamini-Hochberg corrections were performed using custom Python codes available in (Lebedev et al., 2022; Lebedev et al., 2022).

### 2.2 Correlation of dependency data with drug sensitivity

Average dependency scores were calculated using shRNA and CRISPR gene scores from DepMap data^2^ described in (Lebedev et al., 2022). For each cell line we also calculated mean dependency score across all *PSMB1-10* genes. Drug sensitivity data was downloaded from CTRP database.^3^ Briefly, for each colorectal cell line present in DepMap we calculated averaged dependency scores for each of the *PSMB1-10* genes and extracted AUC values from the dataset (Basu et al., 2013). AUC values were converted into sensitivity values as the reverse values and then we calculated Spearman correlation for each drug-gene pair with Benjamini-Hochberg correction. Drugs for the analysis were selected using the ChEMBL database^4^ via searching for compounds that at least entered the phase-2 clinical trials and have indications for colorectal neoplasms. Heatmaps were constructed using ComplexHeatmap package for R (Gu et al., 2016).

### 2.3 Cell culture

Human colorectal adenocarcinoma cell line SW480 and embryonic kidney HEK 293T cell line were kindly provided by Dr. Vladimir Prassolov. The SW620B8-mCherry cell line was obtained previously (Burov et al., 2021). The SW480, SW480B8-mCherry and SW620B8-mCherry and HEK 293T cells were maintained in DMEM (Thermo Fisher Scientific, Paisley, Renfrewshire, Scotland, UK). Cell culture media contained 10% of fetal calf serum (FCS) (Hyclone, Logan, UT, USA), 100 U/mL of penicillin and 100 μg/mL of streptomycin. Cells were incubated at 37°C and 5% CO_2_.

### 2.4 Cell viability assay and treatment of cells with MKIs

The SW480B8-mCherry, SW620B8-mCherry and HEK 293T cells were treated with 0.1–250 µM of regorafenib (MedChemExpress, Monmouth Junction, NJ, USA) and 0.1–500 µM of sorafenib (Sigma-Aldrich, Burlington, MA, USA). Cellular viability was assessed 72 h post-drug-treatment using trypan blue exclusion in Neubauer chamber. To evaluate the effects of MKIs, SW480B8-mCherry were incubated with 1, 5, or 10 µM of regorafenib or sorafenib, while the SW620B8-mCherry cells were treated with 0.5, 2.5, 5 µM of the drugs.

### 2.5 Transfection and cell sorting

The plasmid encoding Cas9D10A, GFP and gRNAs, the donor plasmid used for recombination, as well as the protocol for validation of the obtained cell line were generated previously (Burov et al., 2021). SW480 cells were co-transfected with the plasmids using Mirus TransIT-LT1 reagent (Mirus Bio LLC, Madison, WI, USA) according to manufacturer’s instructions. Forty-eight hours post transfection cells were washed with PBS, detached from the plates using trypsin-EDTA solution (Pan-Eko, Moscow, Russia), centrifuged and resuspended in 1 mL of PBS. Then the FACSAria III cell sorter (BD Biosciences, Franklin Lakes, NJ, USA) was used to obtain the population of cells with considerable GFP fluorescence. After that, cells were cultured for 2 weeks and then stimulated with 1000 U/mL of recombinant human IFN-γ and 500 U/mL of recombinant human TNF-α (both from R&D systems, Minneapolis, MN, USA) for 72 h. Finally, cells with mCherry fluorescence were collected using the FACSAria III cell sorter (BD Biosciences, Franklin Lakes, NJ, USA) and propagated as described above.

### 2.6 Isolation of genomic DNA, total RNA; PCR and real-time PCR

Genomic DNA and total RNA were isolated using GeneJET Genomic DNA Purification Kit (Thermo Scientific, Waltham, MA, USA) and RNA Solo Kit (Evrogen, Moscow, Russia), according to the manufacturer’s recommendations. The concentration and purity of nucleic acids were determined using NanoDrop spectrophotometer (Thermo Scientific, Waltham, MA, USA).

To verify the presence of specific insert in the genome of SW480B8-mCherry cells we used two sets of primers that were obtained previously (Burov et al., 2021) (Supplementary Table S2). The fragments of genomic DNA were amplified with primer pairs A-B and C-D (Burov et al., 2021) using Q5 High-Fidelity DNA polymerase (New England Biolabs, Ipswich, MA, USA). The nucleotide sequence integrity was confirmed by bi-directional sequencing.

The cDNA was obtained from two microgram of total RNA using oligo(dT)20 primer and Magnus Reverse Transcriptase (Evrogen, Moscow, Russia). To estimate chimeric gene (*PSMB8-mCherry*) expression levels we used the G-H pair of primers (Burov et al., 2021) (Supplementary Table S2). For the amplification of the wild-type *PSMB8*, *PSMB5*, *PSMB9* and *PSMB10* genes fragments, primer pairs reported in (Morozov et al., 2019) were utilized (Supplementary Table S2). qPCR reactions were performed as described in (Morozov et al., 2019). The β-Actin (*ACTB*) gene expression was used for normalization. The calculation of the relative expression levels of studied genes was performed using the ΔΔCt method.

### 2.7 Preparation of lysates and Western blotting

Cells were washed two times with PBS, collected and lysed for 10 min on ice in the NP-40 cell lysis buffer (50 mM Tris-Cl (pH 8.0), 150 mM NaCl, 1.0% NP-40), followed by centrifugation for 10 min at 13000×g. Cell supernatants were collected and stored at −80°C before use. Alternatively, cells were lysed directly in the SDS-PAGE Sample buffer (Invitrogen, Waltham, MA, USA). Proteins were separated in 12% Tris-glycine polyacrylamide gels and transferred onto 0.45 µm nitrocellulose membranes (Bio-Rad, Hercules, CA, USA). The membranes were incubated with primary rabbit anti-β1i (Abcam, Cambridge, UK, RRID:AB_303707), or rabbit anti-β2i (Abcam, Cambridge, UK, AB_2895211) or rabbit anti-β5i (Abcam, Cambridge, UK, RRID:AB_303708), or rabbit anti-mCherry (Cell Signaling, Danvers, MA, USA, RRID:AB_2799246), or mouse anti-20S proteasome alpha1,2,3,5,6,7 (Enzo, Farmingdale, NY, USA, RRID:AB_10541045) antibodies and secondary HRP-labeled anti-rabbit or anti-mouse conjugates (Abcam, Cam-bridge, UK, RRID:AB_10679899 or Enzo, Farmingdale, NY, USA, RRID:AB_10540652, respectively). Blots were developed using SuperSignal West Femto Maximum Sensitivity Substrate (Thermo Scientific, Waltham, MA, USA). For signal normalization membranes were striped and treated with mouse anti-β-actin antibodies (Abcam, Cambridge, UK, RRID:AB_306371) and HRP-labeled anti-mouse conjugates. Blots were developed as described.

### 2.8 Immunoprecipitation of proteasomes

For the immunoprecipitation of proteasomes the Proteasome purification kit (Enzo, Farmingdale, NY, USA) was used according to the manufacturer’s instructions. The homogenization of cells was performed in a binding buffer (25 mM HEPES, pH 7.4, 10% glycerol, 5 mM MgCl_2_, 1 mM ATP, 1 mM DTT) via consecutive freezing/thawing. Cells were centrifuged for 30 min at 13,000×g and the supernatants were collected. Obtained samples were incubated with the proteasome purification matrix at 4°C overnight. After brief centrifugation at 5,000×g the supernatants (unbound fraction) were collected. The pellet was washed three times in binding buffer and the proteasomes were eluted using the SDS-PAGE Sample buffer (Invitrogen, Waltham, MA, USA).

### 2.9 Detection of catalytically active proteasome subunits

To detect catalytically active proteasome subunits we used proteasome activity probe—Me4BodipyFL-Ahx3Leu3VS (UbiQbio, Amsterdam, Netherlands) according to the protocol described in (De Jong et al., 2012). Obtained lysates (app. 20 μg of total protein) were mixed with 0.5 µL of probe and incubated for 1 h at 37°C. SDS-PAGE was performed and catalytically active proteasome subunits were revealed by using ChemiDoc XRS+ imaging system (Bio-Rad, Hercules, CA, USA) (excitation wavelength 480 nm and emission wavelength 530 nm). To ensure an equal protein load the gel was incubated with the ROTI®Blue quick protein stain (Carl Roth, Karlsruhe, Germany).

### 2.10 Determination of proteasome activity

Overall proteasome activity was determined using a Me4BodipyFL-Ahx3Leu3VS (UbiQbio, Amsterdam, The Netherlands) proteasome activity probe according to the published protocol (De Jong et al., 2012). Briefly, cells were cultivated in 12 well plates, treated with MKIs and following 72 h the probe was added into the culture media to achieve final concentration of 200 nM. Cells were incubated for 2 hours. After that, cells were washed with PBS and detached from the culture plate using a trypsin solution (PanEco, Moscow, Russia). Then, cells were fixed by continuous shaking in buffer containing 1% of FBS and 0.5% formaldehyde. Detection of fluorescence intensity was performed using LSRFortessa flow cytometer (BD Biosciences, Franklin Lakes, NJ, USA). For the determination of proteasome activity using fluorogenic substrates 72 h after treatment with the MKIs cells were washed with PBS, detached from the surface of the plate using the rubber scrapper, centrifuged and washed again. Cells were homogenized by consecutive freezing/thawing. Chymotrypsyn-like and β5i-specific proteasome activities were measured as described elsewhere (Vagapova et al., 2021) using Suc-LLVY-AMC (Sigma, St. Louis, MO, USA) and Ac-ANW-AMC (Boston Biochem, Cambridge, MA, USA) fluorogenic substrates, correspondingly. Control reactions with 100 nM of the proteasome inhibitor Bortezomib (Selleckchem, Houston, TX, USA) or 1 mM of another proteasome inhibitor MG132 (Santa Cruz Biotechnology Inc., Dallas, Texas, USA) were performed to test nonspecific degradation of substrates. The activities were estimated at the excitation wavelength 380 nm and emission wavelength 440 nm using VersaFluor Fluorometer (Bio-Rad, Hercules, CA, USA). Relative activity levels were obtained following subtraction of the activity levels in samples with proteasome inhibitors from the values detected in lysates.

### 2.11 Confocal microscopy

The SW480, SW480B8-mCherry and SW620B8-mCherry cells were grown on Clip-max culture flasks (TPP, Trasadingen, Switzerland). Twenty-four hours after seeding cells were stimulated with 1000 U/mL of recombinant human IFN-γ (R&D systems, Minneapolis, MN, USA) and 500 U/mL of recombinant human TNF-α (R&D systems, Minneapolis, MN, USA), or regorafenib (MedChemExpress, Monmouth Junction, NJ, USA), or sorafenib (Sigma-Aldrich, Burlington, MA, USA) and incubated for additional 72 h. Prior analysis, cells were incubated for 2 h with 200 nM of the probe Me4BodipyFL-Ahx3Leu3VS (UbiQbio, Amsterdam, The Netherlands). The cells were washed and fixed with 4% PFA (BosterBio, Pleasanton, CA, USA), washed again with PBS and incubated with NucBlue Fixed Cell ReadyProbe (Thermo Scientific, Waltham, MA, USA) for 15 min to stain the nuclei. After that, slides were covered with a SlowFade™ Gold Antifade Mountant (Thermo Scientific, Waltham, MA, USA) and cover slips (Thermo Scientific, Waltham, MA, USA). Samples were analyzed using Leica DMI 6000 CS microscope equipped with a Leica TCS SP5 laser scan unit (Leica, Wetzlar, Germany). All images were acquired in “sequential scan mode” to completely avoid the “bleed-through” effect. A quantitative comparison of label intensities was made by measuring the mean intensity value of pixels (0-255 for 8-bit images) within cytoplasm and nucleus regions using FIJI (ImageJ) software.^5^

### 2.12 Detection of oxidative stress

The SW480B8-mCherry and SW620B8-mCherry cells were grown on 12-well culture plates (TPP, Trasadingen, Switzerland). Twenty-four hours after seeding cells were stimulated with regorafenib (MedChemExpress, Monmouth Junction, NJ, USA) or sorafenib (Sigma-Aldrich, Burlington, MA, USA) and incubated for additional 72 h. Five micromoles of the drugs were used to stimulate SW620B8-mCherry cells and 10 μmol were used in the case of SW480B8-mCherry cell line. The oxidative stress was measured using ROS-ID Hypoxia/Oxidative stress detection kit (Enzo, Farmingdale, NY, USA) according to the manufacturer’s instructions. Briefly, cells were detached from the plate, washed with PBS and incubated for 30 min at 37°C and 5% CO_2_ with the Oxidative stress detection mix. After that, cells were washed with PBS and cellular fluorescence was analyzed by flow cytometry. Control reactions with no oxidative stress detection mix, or 200 µM of the ROS inducer (pyocyanin) were performed. Alternatively, following incubation with MKIs the Oxidative stress detection mix was added directly to the wells, cells were incubated for 30 min at 37°C and 5% CO_2_. Then cells were washed and analyzed using Leica DMi 8 fluorescent microscope (Leica, Wetzlar, Germany).

### 2.13 Flow cytometry

To estimate the sensitivity of the obtained SW480B8-mCherry cell line, the cells were treated with 50, 100, 200, 500 or 1000 U/mL of IFN-γ (R&D systems, Minneapolis, MN, USA), or a combination of 500 U/mL of IFN-γ and 500 U/mL of TNF-α, or 1000 U/mL of IFN-γ and 500 U/mL of TNF-α (R&D systems, Minneapolis, MN, USA). To evaluate the effects of MKIs, SW480B8-mCherry and SW620B8-mCherry cells were treated with the above mentioned concentrations of regorafenib and sorafenib. Following 72 h of incubation, mCherry fluorescence was detected using LSRFortessa flow cytometer (BD Biosciences, Franklin Lakes, NJ, USA). Flow cytometry results were analyzed using FlowJo version 10.0.7 (FlowJo LLC, Ashland, OR, USA; RRID:SCR_008520) and GraphPad Prism version 8.4.3. (GraphPad Software, San Diego, CA, USA; RRID:SCR_002798) software.

### 2.14 Drug combination analysis

To determine the drug combination responses, SW480B8-mCherry and SW620B8-mCherry cells were seeded in 96-well plates in concentrations of 2500 and 5000 cells per well, respectively. After 24 h cells were treated with bortezomib in combination with sorafenib or regorafenib and incubated for additional 72 h. AbiCell Resazurin Cytotoxicity Assay Kit (Abisense, Sirius, Russia) was used for measurement of cell viability. Supernatant was removed and Resazurin in a ratio of 1:100 in DMEM was added to cells. Resazurin assay was measured by 570 nm absorbance and 620 nm reference using Multiskan FC (Thermo Scientific, Waltham, MA, USA) after 4 h incubation at 37°C and 5% CO_2_, reference signal for each well and mean signal for wells containing only growth medium and Resazurin were subtracted before normalization. ZIP synergy scores of drug combinations were calculated using SynergyFinder 3.0 software.^6^

### 2.15 Statistical analyses

The experiments were performed at least in triplicates. Bar carts depicts mean values ±standard deviation for experimental replicates. If other is not indicated, the unpaired two-tailed t-test was used to evaluate the statistical significance of differences between the experimental groups. For all the experiments, *p* values less than 0.05 were regarded as statistically significant. Asterisks indicate: *p < 0.05; **p < 0.01; ***p < 0.001; ****p < 0.0001.

## 3 Results

### 3.1 The expression of non-constitutive proteasomes is increased in BRAF-mutant colorectal tumors

**FIGURE 1:**
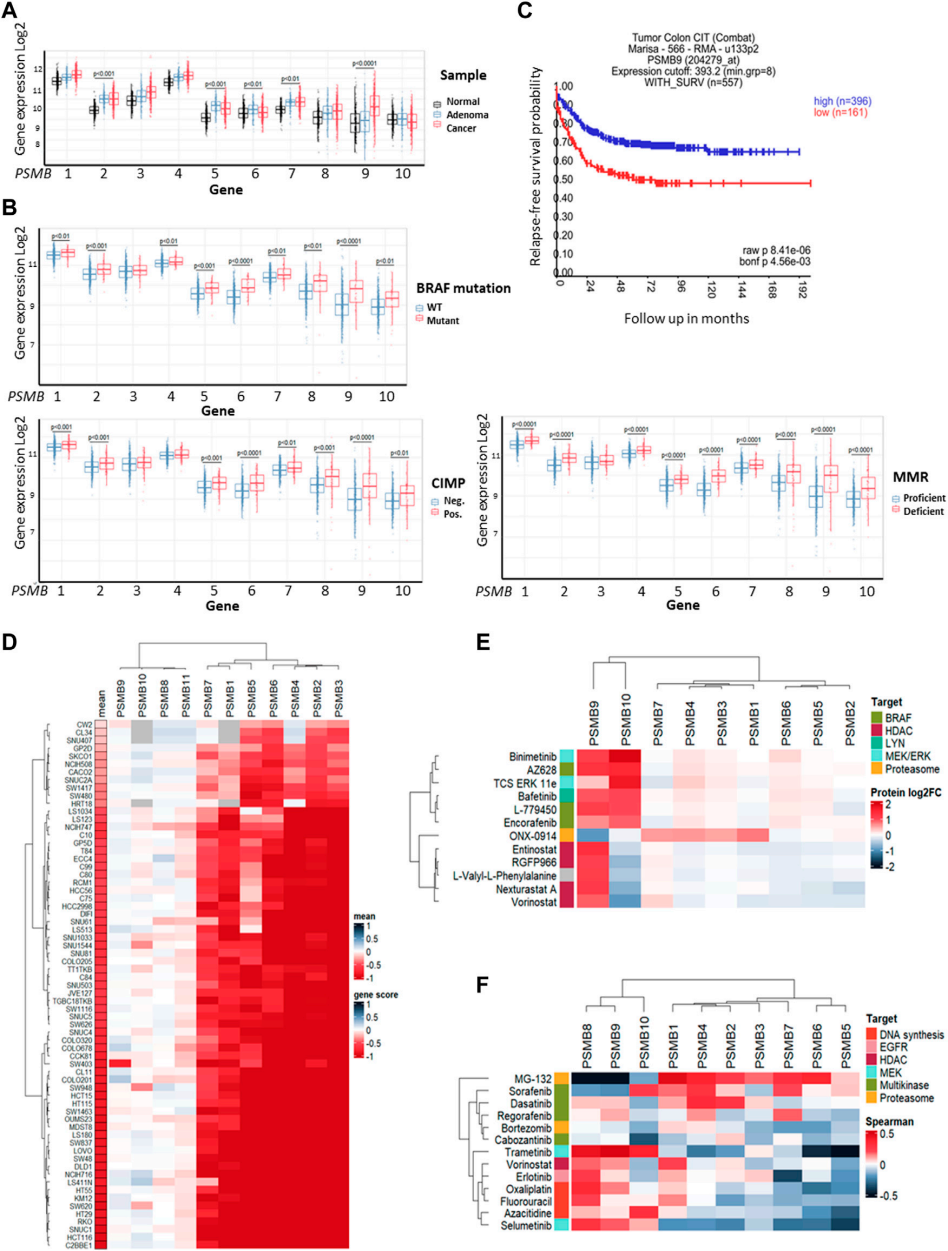
Proteasome genes expression in colorectal cancer and cell lines. **(A)** The *PSMB1-10* expression in normal colon tissue, non-malignant adenoma and colorectal carcinoma. Gene expression data was taken from E-MTAB-10089 dataset and statistical significance was determined using one-way ANOVA test with Benjamini-Hochberg correction. **(B)** Comparison of *PSMB1-10* expression in molecular subtypes of colorectal cancer from GSE39582 dataset. Statistical significance was determined using Mann-Whitney non-parametric test with Benjamini-Hochberg correction. CIMP- CpG island methylator phenotype, MMR—DNA mismatch repair. **(C)** Kaplan-Meier analysis of relapse-free survival for patients with high and low *PSMB9* expression in colorectal tumors. Survival data was taken from GSE39582 dataset and analyzed in R2: Genomics Analysis and Visualization Platform. **(D)** Heatmap of gene dependency scores for colorectal cell lines and *PSMB1-10* genes. Dependency score was calculated based on combined results from DepMap shRNA and CRISPR gene fitness screens. Negative values indicate reduced cell proliferation/survival after gene depletion. For each cell line the mean gene scores across all *PSMB1-10* genes was calculated. **(E)** Heatmap showing protein levels change in HCT-116 cells treated with selected drugs. The data was acquired from proteomic dataset (Mitchell et al., 2023). **(F)** Heatmap showing Sperman’s correlation coefficients for each pair of *PSMB1-10* expression and sensitivity to selected drugs. Cell lines, drugs and genes were clustered using Ward D2 method and heatmaps were generated using ComplexHeatmap package.

First, we investigated colorectal carcinoma-associated patterns of expression of genes that encode 20S proteasome beta subunits (*PSMB1-10*). Using the dataset E-MTAB-10089 (Rohr et al., 2021) the expression levels of relevant genes in normal tissues (*n* = 231), non-malignant colon adenomas (*n* = 132) and colorectal cancer (*n* = 342) were compared. Most of the *PSMB1-10* genes were upregulated in adenomas and cancer compared to normal tissue, however only expression of the *PSMB9* (encodes immune subunit β1i) was significantly higher in colorectal cancer compared to non-malignant adenomas (Figure 1A). To further elucidate the link between *PSMB1-10* genes expression and colorectal cancer we compared gene expression in different molecular subtypes of colorectal cancer from GSE39582 dataset (Marisa et al., 2013): tumors with BRAF, KRAS, or TP53 mutations, CpG island methylator phenotype (CIMP), DNA mismatch repair deficiency (MMR), or chromosomal instability (CIN). Most of the *PSMB1-10* genes, including immune subunit genes *PSMB8* (encodes immune subunit β5i), *PSMB9*, and *PSMB10* (encodes immune subunit β2i) were upregulated in BRAF mutant, CIMP positive and MMR deficient tumors, with *PSMB6* (encodes constitutive subunit β1) and *PSMB9* having the highest difference (Figure 1B). For TP53 and KRAS mutant tumors we found no statistical difference in expression of any of the *PSMB1-10* genes. Tumors with chromosomal instability had lower expression of *PSMB5* (encodes constitutive subunit β5), *PSMB6* and *PSMB10* genes (Supplementary Figure S1). Interestingly, upregulation of *PSMB9* was associated with more favorable relapse-free survival as was revealed by Kaplan-Meier analysis (Figure 1C).

Next, we used gene fitness data from DepMap database (Tsherniak et al., 2017; McFarland et al., 2018; Behan et al., 2019) to investigate the role of proteasome genes in colorectal cancer cell survival. For each of 67 colorectal cell lines and *PSMB1-10* genes we calculated an averaged gene dependency score (Lebedev et al., 2022) that represents how gene expression reduction by shRNA or CRISPR/Cas-9 system affected cell proliferation and survival. The *PSMB1-7* were essential for most colorectal cancer cell lines, while only few cell lines dependent on expression of immune subunits *PSMB8-10* (Figure 1D). Since non-constitutive proteasome subunits expression has the most pronounced changes in BRAF-mutant tumors, we sought to identify which drugs can affect immune proteasome subunits. We used proteome data for HCT-116 cells treated with 875 drugs (Mitchell et al., 2023) and selected drugs which changed β1i or β2i (the β5i levels were not included in the dataset) levels at least two-fold (Supplementary Table S1). Among 815 drugs we found 12 drugs which affect non-constitutive proteasome protein levels: ONX-0914, HDAC inhibitors entinostat, nexturastat-a, RGFP-996, and vorinostat, RAF inhibitors encorafenib, AZ-628, and L-779450, MEK inhibitor MEK-162, ERK1/2 inhibitor TCS-ERK-11e and LYN inhibitor bafetinib. Notably, only RAF, MEK and LYN inhibitors increased both β1i and β2i and did not affect protein levels of other subunits (Figure 1E). These findings suggest that although non-constitutive proteasomes are not essential for colorectal cell survival their protein levels change specifically in response to BRAF inhibitors, pointing on their role in drug response.

As the next step we analyzed the correlation of *PSMB1-10* gene expression in colorectal cell lines with their sensitivity to selected 11 drugs from ChEMBL database (https://www.ebi.ac.uk/chembl) that are approved for colorectal cancer treatment or undergo clinical trials from CTRP dataset (Basu et al., 2013). We also included two proteasome inhibitors MG-132 and bortezomib, and as expected, the sensitivity to proteasome inhibitor MG-132 positively correlated with expression of constitutive subunits. Multikinase inhibitors (MKIs) sorafenib, dasatinib, regorafenib and cabozantinib were clustered with proteasome inhibitors bortezomib and MG-132 (Figure 1F). Sensitivity to sorafenib, which also inhibits BRAF kinase, negatively correlated with *PSMB8* and *PSMB9* expression, suggesting that BRAF-mutant tumors with high *PSMB9* expression might be less sensitive to BRAF inhibitors and BRAF inhibitors may modulate the expression of non-constitutive proteasomes.

### 3.2 Generation of SW480B8-mCherry cell line

**FIGURE 2:**
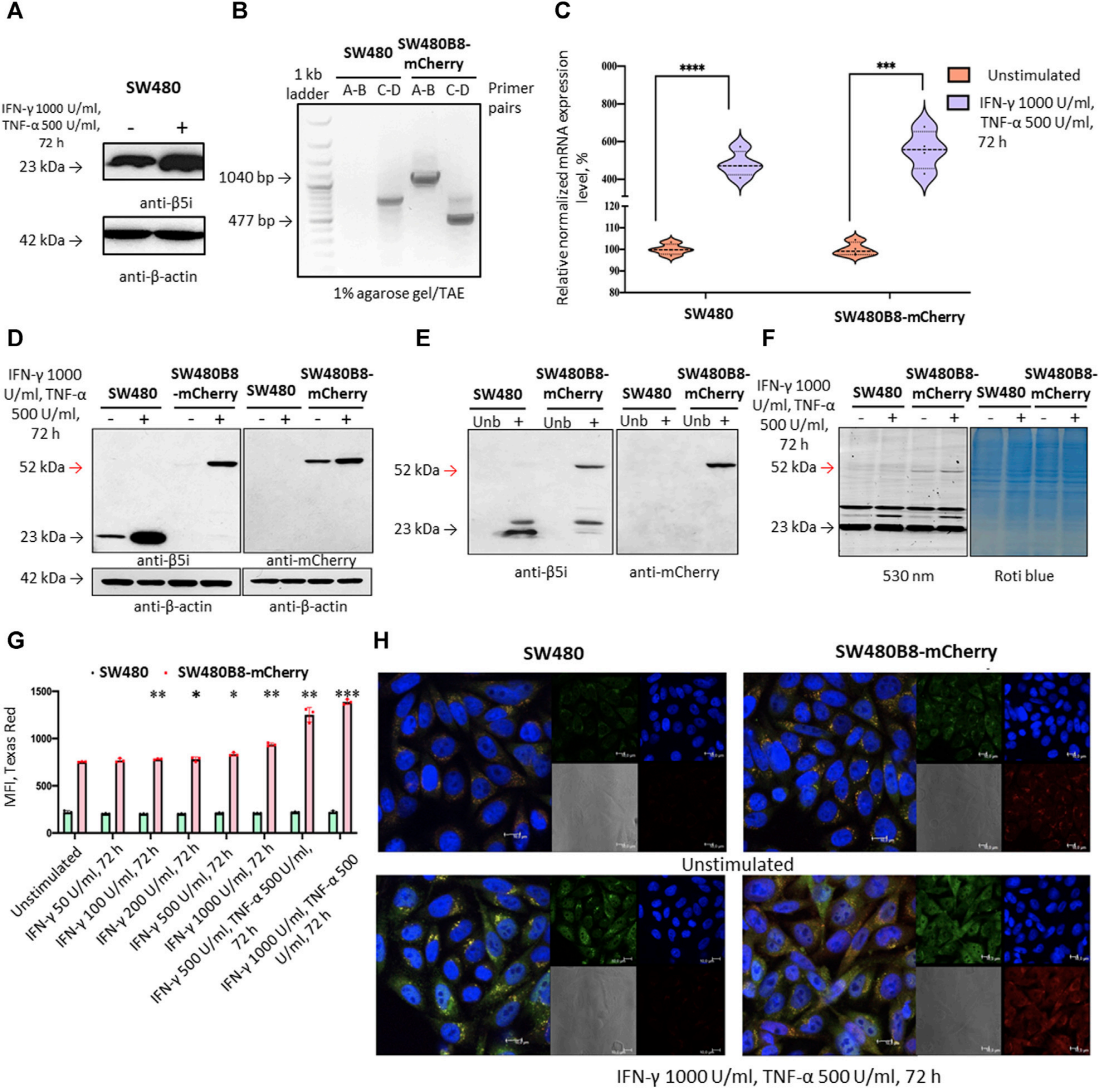
The mCherry gene is integrated into the genome and expressed in modified cells. The β5i-mCherry chimera is integrated into the proteasome and is an active proteasome subunit in SW480B8-mCherry cells. **(A)** Western blotting of lysates obtained from SW480 cells and SW480 cells treated with 1000 U/mL IFN-γ and 500 U/mL of TNF-α for 72 h. Membrane was incubated with anti- β5i, stripped and incubated with anti-β-actin antibodies. **(B)** Modification of the genomic DNA in SW480B8-mCherry cells was confirmed by PCR with two sets of primers (**(A–D)** (Supplementary Table S2) (Burov et al., 2021)). The amplicons of the anticipated size (1040 and 477 bp) were observed in samples from SW480B8-mCherry cells, but not control SW480 cells. **(C)** The relative expression levels of *PSMB8* and *PSMB8-mCherry* mRNA in unstimulated SW480 and SW480B8-mCherry cells and cells treated with IFN-γ (1000 U/mL) and TNF-α (500 U/mL) for 72 h, correspondingly. **(D)** Western blot of lysates obtained from unstimulated SW480 and SW480B8-mCherry, and cells treated with 1000 U/mL IFN-γ and 500 U/mL of TNF-α for 72 h. The membranes were incubated with either anti-β5i or anti-mCherry antibodies. **(E)** Immunoprecipitation of proteasomes from lysates of IFN-γ and TNF-α- stimulated SW480 and SW480B8-mCherry cells. Proteasomes were precipitated using agarose immobilized anti-α4 antibodies (Enzo, Farmingdale, NY, USA). Western blot of immunoprecipitated proteasomes. The membranes were incubated with either anti-β5i or anti-mCherry antibodies. **(F)** The β5i-mCherry is catalytically active subunit within the proteasomes of SW480B8-mCherry cells. Homogenates of unstimulated control and cytokine-stimulated (1000 U/mL of IFN-γ and 500 U/mL of TNF-α for 72 h) modified cells were incubated for 1 h at 37°C with the Me4BodipyFL-Ahx3Leu3VS probe. The fluorescence of proteasome subunits was analyzed in 13% Tris-Glycine polyacrylamide gel. The analysis was performed at the excitation wavelength 480 nm and emission wavelength 530 nm (left panel). The same gel stained with Roti blue quick protein stain is shown on the right panel. **(G)** The fluorescence of SW480 and SW480B8-mCherry cells treated with different concentrations of IFN-γ or combinations of IFN-γ and TNF-α for 72 h. Tests were performed in triplicates. Median fluorescence intensity (MFI) for 10000 of cells is shown. **(H)** Proteasomes with β5i-mCherry subunit were revealed in the nucleus and cytoplasm of SW480B8-mCherry cells. Confocal microscopy of unstimulated and cytokine-treated (1000 U/mL IFN-γ and 500 U/mL TNF-α) SW480 and SW480B8-mCherry cells. Proteasomes containing subunits with fused mCherry could be detected by red fluorescence. To reveal the proteasome activity within cells, cells were incubated for 2 h with Me4BodipyFL-Ahx3Leu3VS (green fluorescence). In addition fixed cell nuclei were stained with NucBlue Fixed Cell ReadyProbe (seen as the blue fluorescence). Ns- not significant; *p < 0.05; **p < 0.01; ***p < 0.001; t-test.

To investigate the effect of MKIs on the expression of non-constitutive proteasomes we used previously obtained cell line SW620B8-mCherry (Burov et al., 2021). The cells synthesize proteasomes containing the immune subunit β5i (a component of immunoproteasomes and most types of intermediate proteasomes (Guillaume et al., 2010)) fused with a red fluorescent protein mCherry. The unique feature of SW620 cell line is that it was derived from metastasis of patient with colorectal carcinoma and there is a cell line (SW480) that was obtained from the primary tumor of the same patient. Combination of these two cell lines allows studying late phases of colon cancer progression (Hewitt et al., 2000). Although these cell lines belong to the same patient they displayed different dependencies on proteasome expression: SW480 had one of the lowest dependencies on proteasome expression, while SW620 had one of the highest dependencies (Figure 1D). Therefore, we sought to investigate and compare the effect of MKIs on both cell lines. In this regard, we demonstrated that SW480 cells express β5i (Figure 2A) and performed the same genetic modifications with SW480 cells, as we did with SW620 cell line to label non-constitutive proteasomes (Burov et al., 2021). Using CRISPR/Cas9 system we introduced gene encoding the mCherry in the same open reading frame to the 3’ end of the last exon of the *PSMB8* gene which encodes the β5i subunit. The SW480 cells were transfected with previously obtained plasmids and treated as described in (Burov et al., 2021). The presence of the insert in genomic DNA of SW480B8-mCherry cells was confirmed by PCR with two sets of primers (Figure 2B). Then, using real-time PCR it has been shown that combination of IFN-γ and TNF-α (cytokines that activate immunoproteasome subunit expression (Aki et al., 1994)) induce comparable increase in the level of *PSMB8* transcripts in wild-type SW480 cells and *PSMB8-mCherry* transcripts in SW480B8-mCherry cells, indicating conservation of endogenous regulatory mechanisms of *PSMB8* expression in modified cells (Figure 2C). The protein with molecular mass (∼52 kDa) corresponding to a β5i–mCherry chimera was revealed in lysates of SW480B8-mCherry, but not control SW480 cells using antibodies to both β5i and mCherry (Figure 2D). Importantly, no free mCherry was detected in lysates of modified cells. To verify that the chimeric subunit is integrated into proteasomes we performed immunoprecipitation with antibodies to the non-catalytic proteasome subunit α4. We demonstrated presence of the chimeric protein in the precipitate obtained from IFN-γ and TNF-α-stimulated SW480B8-mCherry cells (Figure 2E). In order to verify that the chimeric subunit is catalytically active, cytokine-stimulated control and modified cells were incubated with Me4BodipyFL-Ahx3Leu3VS proteasome activity probe. The probe allows visualization of proteolytic proteasome subunits via binding to the N-terminal catalytic threonine residue (Berkers et al., 2007; De Jong et al., 2012). The interaction of the probe with the 52 kDa protein was observed in lysates of SW480B8-mCherry cells (Figure 2F). To estimate the sensitivity of the cell line to modulators that affect expression of the immune subunits, we incubated SW480 and SW480B8-mCherry cells for 72 h with different concentrations of IFN-γ and combinations of IFN-γ with TNF-α. Cell fluorescence was analyzed by flow cytometry. The significant difference (*p* < 0.01, t-test) in mCherry fluorescence between control and cytokine-stimulated SW480B8-mCherry cells was observed when 100 U/mL of IFN-γ was used. It should be mentioned that after incubation of cells with 50 U/mL the difference in fluorescence was close to significant (*p* = 0.067) indicating high sensitivity of the obtained cell line to modulation of the immune subunit expression (Figure 2G). No difference in fluorescence was observed when control SW480 cells were incubated with different concentrations of cytokines. The localization of non-constitutive proteasomes in SW480B8-mCherry was studied by confocal microscopy (Figure 2H). Within the unstimulated cells, proteasomes with mCherry-labelled β5i were mostly localized in the cytoplasm where they were either equally dispersed or formed small optically dense aggregates (Figure 2H). Stimulation of cells with IFN-γ and TNF-α lead to a significant increase of fluorescence and relocation of certain amount of non-constitutive proteasomes into cell nuclei. Proteasome-containing aggregates were still observed in the cytoplasm of treated cells (Figure 2H). Taken together, a new genetically-modified cell line was obtained, validated and could be used to address the effect of MKIs on non-constitutive proteasome expression and localization.

### 3.3 Regorafenib and sorafenib modulate non-constitutive proteasome subunit expression in colorectal cancer cells

Kinase inhibitors were used in cancer treatment since approval of imatinib in 2001 (Lee et al., 2023). Currently there are 77 FDA-approved small molecule protein kinase inhibitors (as of 19 September 2023, www.brimr.org/PKI/PKIs.htm) and new inhibitors are on the way. Based on the analysis of published datasets, BRAF and multikinase inhibitors were top candidates that could affect non-constitutive proteasome levels (Figure 1E), thus we selected multikinase inhibitors sorafenib and regorafenib which can target BRAF for further experiments.

**FIGURE 3:**
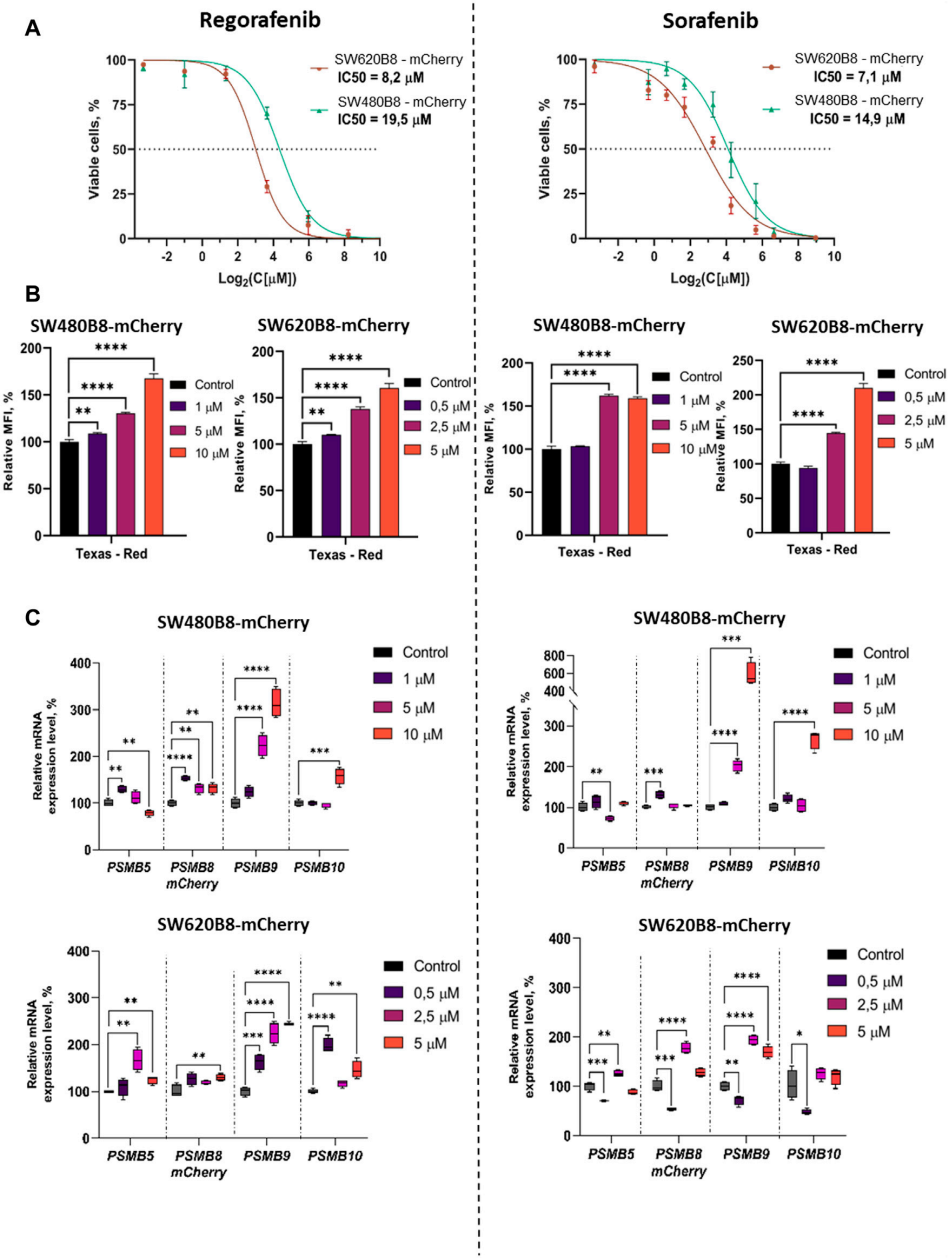
Regorafenib and sorafenib modulate expression of proteasome subunits genes in SW620B8-mCherry and SW480B8-mCherry cells. **(A)** Viability of cells treated with regorafenib or sorafenib. SW620B8-mCherry and SW480B8-mCherry cells were treated with 0.1–250 µM of regorafenib (left panel) and 0.1–500 µM of sorafenib (right panel). Cellular viability was evaluated 72 h post drug-treatment using trypan-blue exclusion. Data represents mean ± SEM of three independent experiments. **(B)** Effects of regorafenib and sorafenib on mCherry fluorescence in SW620B8-mCherry and SW480B8-mCherry cells evaluated by flow cytometry. The SW480B8-mCherry cells were treated with 1, 5 10 µM of regorafenib (left panel) or sorafenib (right panel) and SW620B8-mCherry cells were treated with 0.5, 2.5, 5 µM of regorafenib (left panel) or sorafenib (right panel) for 72 h. The mCherry fluorescence was measured using LSRFortessa flow cytometer (BD Biosciences, Franklin Lakes, NJ, United States). Yellow-green laser and Texas Red filter were used. Normalized median fluorescence intensity (MFI) of 10000 cells is shown. **(C)** Proteasome gene expression levels in cells treated with the MKIs. The relative expression levels of *PSMB5*, *PSMB8-mCherry*, *PSMB9* and *PSMB10* mRNA were determined by qPCR after 72 h-long incubation with the MKIs. *—p < 0.05; **—p < 0.01; ***—p < 0.001; ***—p < 0.001; ****—p < 0.0001, t-test.

We initially studied the viability of SW480B8-mCherry and SW620B8-mCherry cells after incubation with different concentrations of regorafenib and sorafenib. Comparing with the SW480B8-mCherry cells, SW620B8-mCherry cells were considerably more sensitive to regorafenib (IC50: 19.5 vs. 8.2 µM) (Figure 3A). When cell lines were incubated with sorafenib, SW620B8-mCherry cells were also found to be more sensitive with the IC50 of 7.1 µM (Figure 3A). The IC50 value for SW480B8-mCherry cells after sorafenib treatment was 14.9 µM (Figure 3A). Therefore, for the subsequent experiments we used 1, 5, 10 µM of drugs for SW480B8-mCherry cells and 0.5, 2.5, 5 µM of drugs–for SW620B8-mCherry cells. Importantly, used concentrations match the concentrations observed in plasma of patients under treatment with the inhibitors (Fucile et al., 2015; Kobayashi et al., 2021). We also tested the viability of non-cancerous HEK 293T cells following treatment with the inhibitors. It was demonstrated that when cells were incubated with different concentrations of sorafenib the IC50 was estimated as 32.7µM, while incubation with regorafenib yielded IC50 value of 44.6 µM (Supplementary Figure S2).

To investigate the effect of MKIs on the expression of non-constitutive proteasomes SW480B8-mCherry and SW620B8-mCherry cells treated for 72 h with selected concentrations of the drugs were firstly analyzed by flow cytometry. It was demonstrated that 1 μM and 0.5 µM of regorafenib induced increased fluorescence of mCherry in SW480B8-mCherry and SW620B8-mCherry cells, respectively (t-test, **p < 0.01) (Figure 3B). At the same time, these concentrations of sorafenib had no statistically significant effect on the cell lines (Figure 3B). Higher concentrations of both drugs induced statistically significant increase (up to 2.1 fold) of the median fluorescence intensity (MFI) of cells (t-test, ****p < 0.0001) (Figure 3B). Obtained data indicated activated expression of the β5i subunit in studied cell lines upon treatment with MKIs.

To verify the results and to explore how the expression of other proteasome subunits is modulated, we performed qPCR to estimate the mRNA levels of *PSMB8-mCherry*, *PSMB5*, *PSMB9*, *PSMB10* genes encoding chimeric subunit (β5i-mCherry), constitutive proteasome subunit β5 and immune proteasome subunits β1i and β2i, respectively. Increased levels of immunoproteasome subunit mRNAs were revealed in cells following the 72 h incubation with both regorafenib and sorafenib. The expression of *PSMB9* was activated the most and increased up to 6 folds (t-test, ****p < 0.0001), *PSMB10* transcripts levels were increased by 2.9 folds (t-test, ****p < 0.0001) and *PSMB8-mCherry*—by 1.8 folds (t-test, ****p < 0.0001) (Figure 3C). The changes of the *PSMB5* expression were less pronounced reaching maximum 1.6 folds (t-test, **p < 0.01) in SW620B8-mCherry cells incubated with regorafenib (Figure 3C). In SW480B8-mCherry cells a modest decrease of the *PSMB5* expression level was observed following the incubation with 10 µM of regorafenib and 5 µM of sorafenib (t-test, **p < 0.01) (Figure 3C).

**FIGURE 4:**
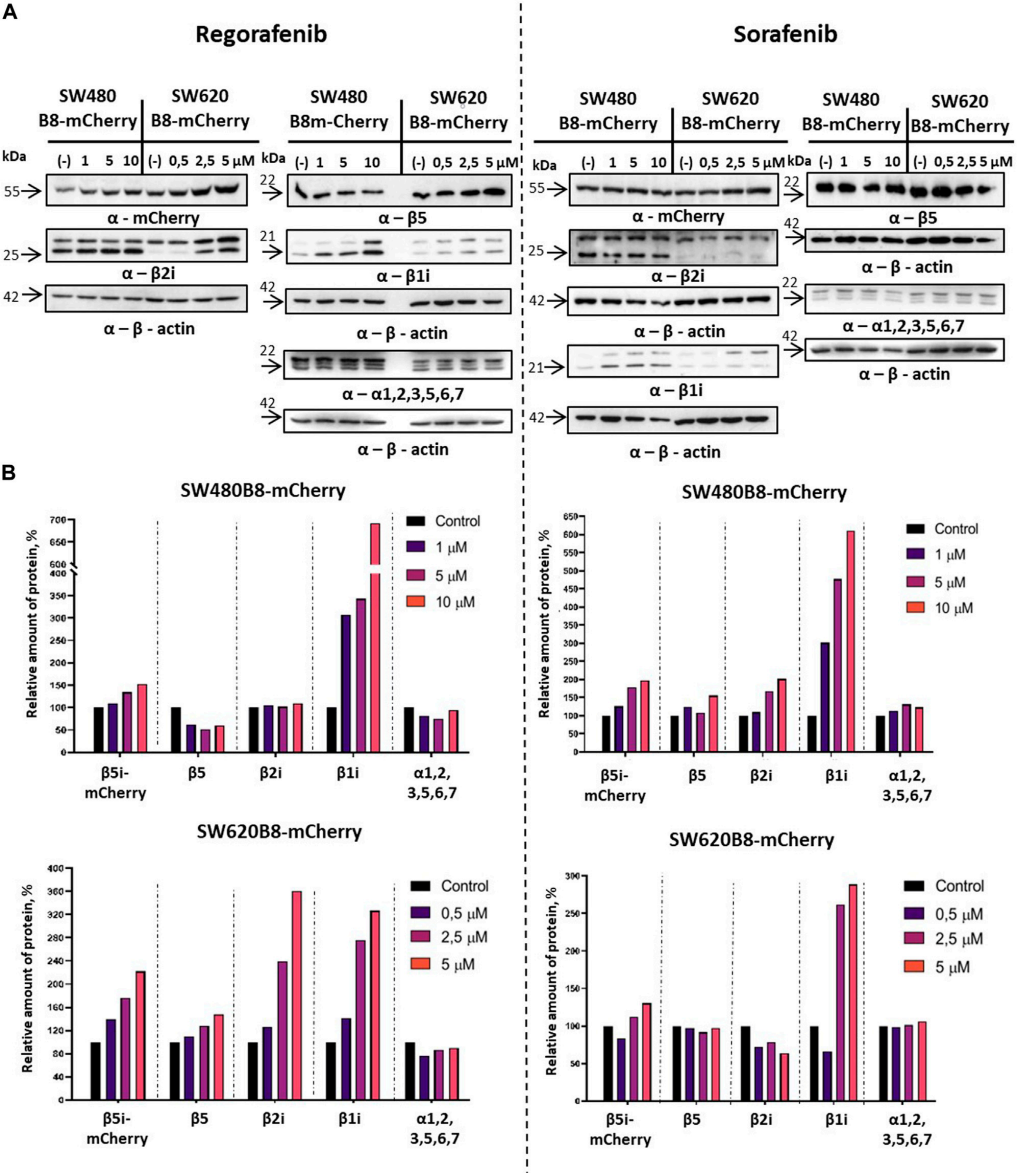
Regorafenib and sorafenib activate synthesis of non-constitutive proteasomes in SW620B8mCherry and SW480B8-mCherry cells. Western blotting of lysates obtained from SW480B8-mCherry and SW620B8-mCherry cells treated with different concentrations of regorafenib (left panel), or sorafenib (right panel) for 72 h **(A)**. Lysates were obtained using the NP-40 lysis buffer. Membranes were incubated with either anti-β1i, or anti-β2i, or anti-β5, or anti-mCherry, or anti-20S proteasome α1,2,3,5,6,7 antibodies, stripped and incubated with anti-β-actin antibodies. **(B)** Evaluation of **(A)** data using the ImageJ software.

Revealed alterations of immune subunits expression were further confirmed by Western blotting of cytoplasmic lysates, as we observed drastic increase in the amount of β1i, β2i and β5i-mCherry subunits in SW620B8-mCherry, as well as the β1i and β5i-mCherry, in SW480B8-mCherry cells treated with regorafenib (Figure 4A). The proteasome subunits are synthesized as precursor molecules and undergo autocatalytic cleavage of propeptides during the late stages of proteasome assembly. Interestingly, we detected lower amounts of processed β1i and β2i subunits in unstimulated SW620B8-mCherry cells comparing to SW480B8-mCherry cells, highlighting differing proteasome pools and prevalence of intermediate proteasomes in SW620B8-mCherry cells. After treatment with regorafenib, however, the quantity of these two subunits rose significantly, favoring increase in the amount of “classical” immunoproteasomes in SW620B8-mCherry cells (Figures 4A, B). Of note, the quantity of precursor protein and processed β1i also increased considerably in SW480B8-mCherry cells indicating rearrangement of the proteasome pool in these cells as well (Figures 4A, B). The sorafenib stimulated accumulation of β1i and β5i-mCherry subunits in both cell lines and the β2i in SW480B8-mCherry cells. Importantly, we detected no differences in the amount of structural alpha proteasome subunits in cytoplasmic lysates, as well as the modest alterations (except decrease following treatment with regorafenib of SW480B8-mCherry cells) in the amount of constitutive β5 subunit indicating that overall quantity of proteasomes changes insignificantly following the exposure to MKIs and rather the rearrangement of the proteasome pool takes place (Figures 4A, B).

### 3.4 Regorafenib and sorafenib modulate proteasome activity and localization in colorectal cancer cells

**FIGURE 5:**
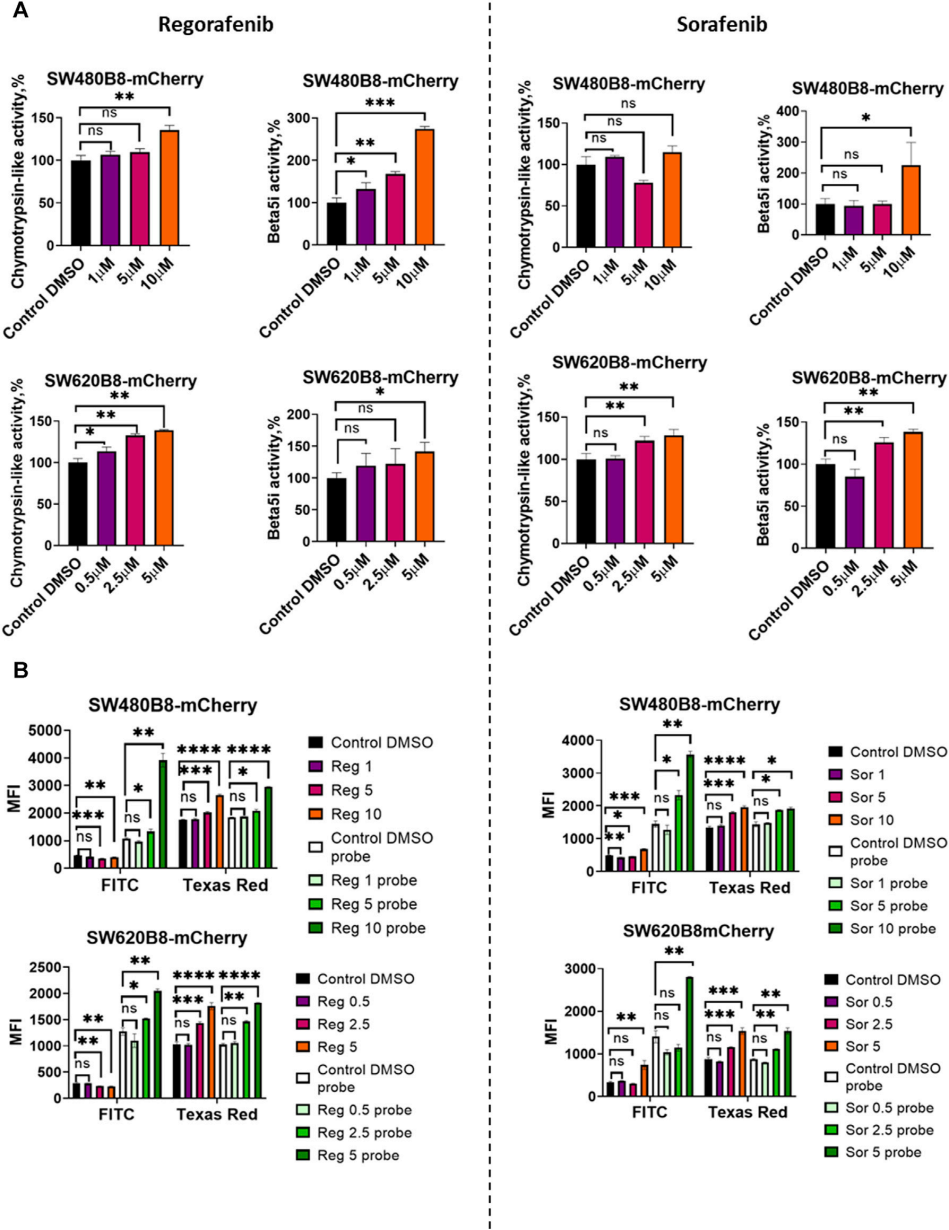
Regorafenib and sorafenib modulate activity of proteasomes in SW620B8mCherry and SW480B8-mCherry cells. **(A)** Chymotrypsin-like and β5i-specific proteasome activity in homogenates of SW480B8-mCherry and SW620B8-mCherry cells treated with different concentrations of regorafenib (left panel), or sorafenib (right panel) for 72 h. The activity was determined using Suc-LLVY-AMC and Ac-ANW-AMC fluorogenic substrates, correspondingly. **(B)** Analysis of BodipyFL and β5i-mCherry fluorescence and proteasome activity in modified cells following incubation with different concentrations of regorafenib (left) and sorafenib (right) by flow cytometry. Treated cells were incubated for 2 h with Me4BodipyFL-Ahx3Leu3VS probe before being analyzed. The BodipyFL and mCherry fluorescence was measured using LSRFortessa flow cytometer (BD Biosciences, Franklin Lakes, NJ, United States). Blue, yellow-green lasers, FITC and Texas Red filters were used, correspondingly. Tests were performed in triplicates. Ns - not significant; *—*p* < 0.05; **—*p* < 0.01; ***—*p* < 0.001; ***—*p* < 0.001; ****—*p* < 0.0001, t-test.

**FIGURE 6:**
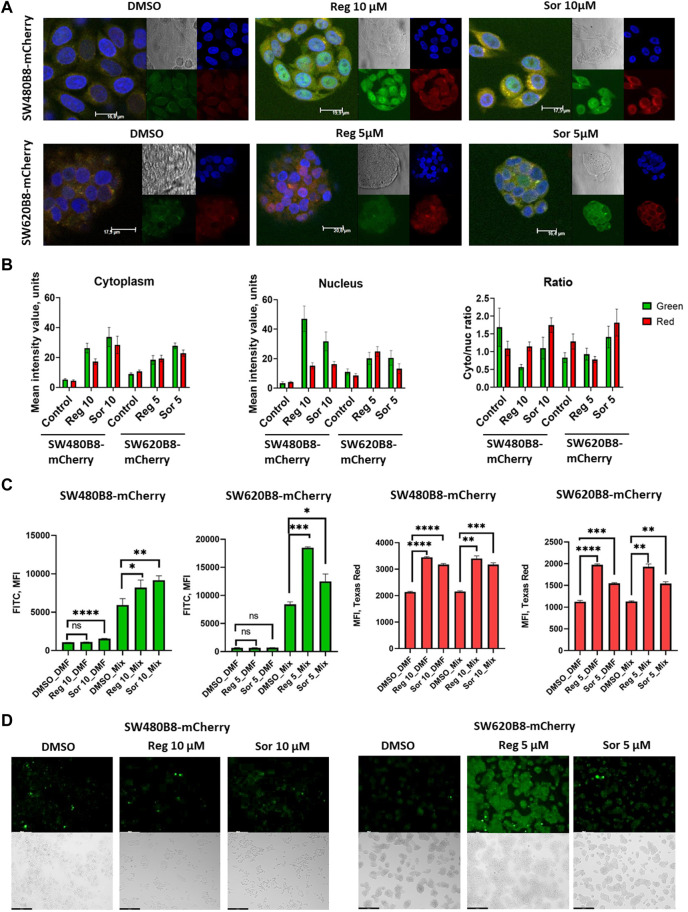
Regorafenib and sorafenib modulate localization of proteasomes and induce oxidative stress in SW620B8mCherry and SW480B8-mCherry cells. **(A)** Activity and localization of proteasomes is altered in modified cells incubated with regorafenib and sorafenib. Confocal microscopy of unstimulated or treated with 10 µM of regorafenib or sorafenib SW480B8-mCherry cells (upper panel). Confocal microscopy of unstimulated or treated with 5 µM of regorafenib or sorafenib SW620B8-mCherry cells (lower panel). Prior microscopy cells were additionally incubated for 2 h with proteasome activity probe Me4BodipyFL-Ahx3Leu3VS (green fluorescence). The mCherry fluorescence is shown in red, while cell nuclei were visualized using NucBlue Fixed Cell ReadyProbe (blue fluorescence). **(B)** Quantification of mean fluorescence intensity of the probe Me4BodipyFL-Ahx3Leu3VS (green) and the mCherry (red) within cytoplasm and nucleus of the cells shown in **(A)**. Additionally Cytoplasm/nucleus fluorescence ratio was calculated (right panel). Calculations were performed using at least 6 representative cells using the ImageJ software. Analysis of oxidative stress in sorafenib and regorafenib treated cells **(C, D)**. Twenty-four hours after seeding the SW480B8-mCherry and SW620B8-mCherry cells were stimulated with regorafenib or sorafenib and incubated for additional 72 h. The SW480B8-mCherry were incubated with 10 µM of regorafenib or sorafenib, while the SW620B8-mCherry cells were treated with 5 µM of the drugs. Control cells were treated with DMSO. The oxidative stress was measured using ROS-ID Hypoxia/Oxidative stress detection kit (Enzo, Farmingdale, NY, USA) according to the manufacturer’s instructions. Cellular fluorescence was analyzed by flow cytometry **(C)** or fluorescent microscopy **(D)**. Reactive oxygen species production can be deduced from changes in green fluorescence, while β5i-mCherry expression is revealed by mCheery fluorescence. For the evaluation of cellular fluorescence by flow cytometry the LSRFortessa flow cytometer (BD Biosciences, Franklin Lakes, NJ, United States) equipped with blue, yellow-green lasers, FITC and Texas Red filters was used. Median fluorescence intensity (MFI) for 10000 of cells is shown. Tests were performed in triplicates. Ns - not significant; *—*p* < 0.05; **—*p* < 0.01; ***—*p* < 0.001; ***—*p* < 0.001; ****—*p* < 0.0001, t-test. Scale bar—250 µm.

Obtained data indicated possible modulation of proteasome activity by selected MKIs. Thus, we sought to evaluate chymotrypsin-like and β5i-specific proteasome activities in homogenates of treated cells. It was demonstrated that following treatment with regorafenib both activities were increased in SW620B8-mCherry and SW480B8-mCherry (Figure 5A). Interestingly, the β5i-specific activity in SW480B8-mCherry cells was increased by 2.8 folds (t-test, ***p < 0.001) and in SW620B8-mCherry–by 1.4 folds (t-test, *p < 0.05) following incubation with the highest concentration of the MKI. Sorafenib treatment of SW480B8-mCherry cells resulted in increased β5i-specific activity (by 2.1 folds, t-test, *p < 0.05) but minimally affected chymotrypsin-like activity in cellular homogenates (Figure 5A). At the same time, both activities were increased in SW620B8-mCherry cells following the incubation with sorafenib (t-test, **p < 0.01) (Figure 5A). We further evaluated overall proteasome activity using the proteasome activity probe - Me4BodipyFL-Ahx3Leu3VS and flow cytometry. It was demonstrated that 5 µM of regorafenib induced significant elevation (t-test, *p < 0.05) of proteasome activity in SW480B8-mCherry cells, 2.5 µM of regorafenib increased the activity in SW620B8-mCherry cells (t-test, *p < 0.05), while maximal concentrations of regorafenib or sorafenib induced up to 3.5 folds (t-test, **p < 0.01) elevation of proteasome activity in both cell lines (Figure 5B). Confocal microscopy of cells treated with Me4BodipyFL-Ahx3Leu3VS and 5 and 10 µM of regorafenib or sorafenib, respectively revealed significant increase of BodipyFL and mCherry fluorescence (Figures 6A, B). Interestingly, following the treatment with regorafenib the ratio of cytoplasmic/nuclear mCherry fluorescence (comparing to control) did not change (SW480B8-mCherry cells) or was decreased (SW620B8-mCherry cells). At the same time, SW480B8-mCherry cells demonstrated decreased ratio of cytoplasmic/nuclear BodipyFL fluorescence following treatment with regorafenib. Accumulation of nuclear BodipyFL fluorescence in SW480B8-mCherry cells together with decreased amount of constitutive β5 subunit in the cytoplasmic lysates might indicate accumulation of large amount of constitutive proteasomes in the nuclei of the cells following treatment with regorafenib. In contrast to regorafenib effects, after incubation with sorafenib the cytoplasmic/nuclear mCherry fluorescence was increased in both cell lines, indicating different localization of non-constitutive proteasomes following treatment with different MKIs (Figure 6B). In SW480B8-mCherry cells, as after the treatment with regorafenib, a decreased ratio of cytoplasmic/nuclear Bodipy fluorescence was observed following incubation with sorafenib. Thus, at least in the case of sorafenib treatment of SW480B8-mCherry cells a translocation of constitutive proteasomes into the nuclei might take place, but more prominent (comparing to regorafenib) retention of non-constitutive proteasomes in the cytoplasm is observed, likely indicating separation of proteasome pool (Figures 6A, B). Interestingly, active non-constitutive proteasomes were revealed in aggregate-like structures near the nuclei and within the cytoplasm of sorafenib-treated cells (Figure 6A). Obtained results indicate putative specific role of non-constitutive proteasomes following the treatment with MKIs and especially sorafenib.

### 3.5 MKIs stimulate production of reactive oxygen species in colorectal cancer cells

Formation of proteasome-containing intracellular aggregates is frequently observed in stress conditions (Enenkel et al., 2022). Thus, we investigated if regorafenib and sorafenib induce the oxidative stress in SW620B8-mCherry and SW480B8-mCherry cells. Cells were treated with 5 μM, or 10 µM of the drugs, respectively for 72 h. It has been shown that both sorafenib and regorafenib stimulated production of reactive oxygen species (ROS). Depending on the cell line, the elevation of ROS concentration was from 1.4 folds (t-test, *p < 0.05; SW480B8-mCherry cells treated with 10 µM of regorafenib) to 2.2 folds (t-test, ***p < 0.001; SW620B8-mCherry cells treated with 5 µM of regorafenib) (Figures 6C, D). Thus, our results indicate that MKIs induce oxidative stress in studied cell lines.

### 3.6 Sorafenib or regorafenib cause no synergistic or additive action when combined with bortezomib

**FIGURE 7:**
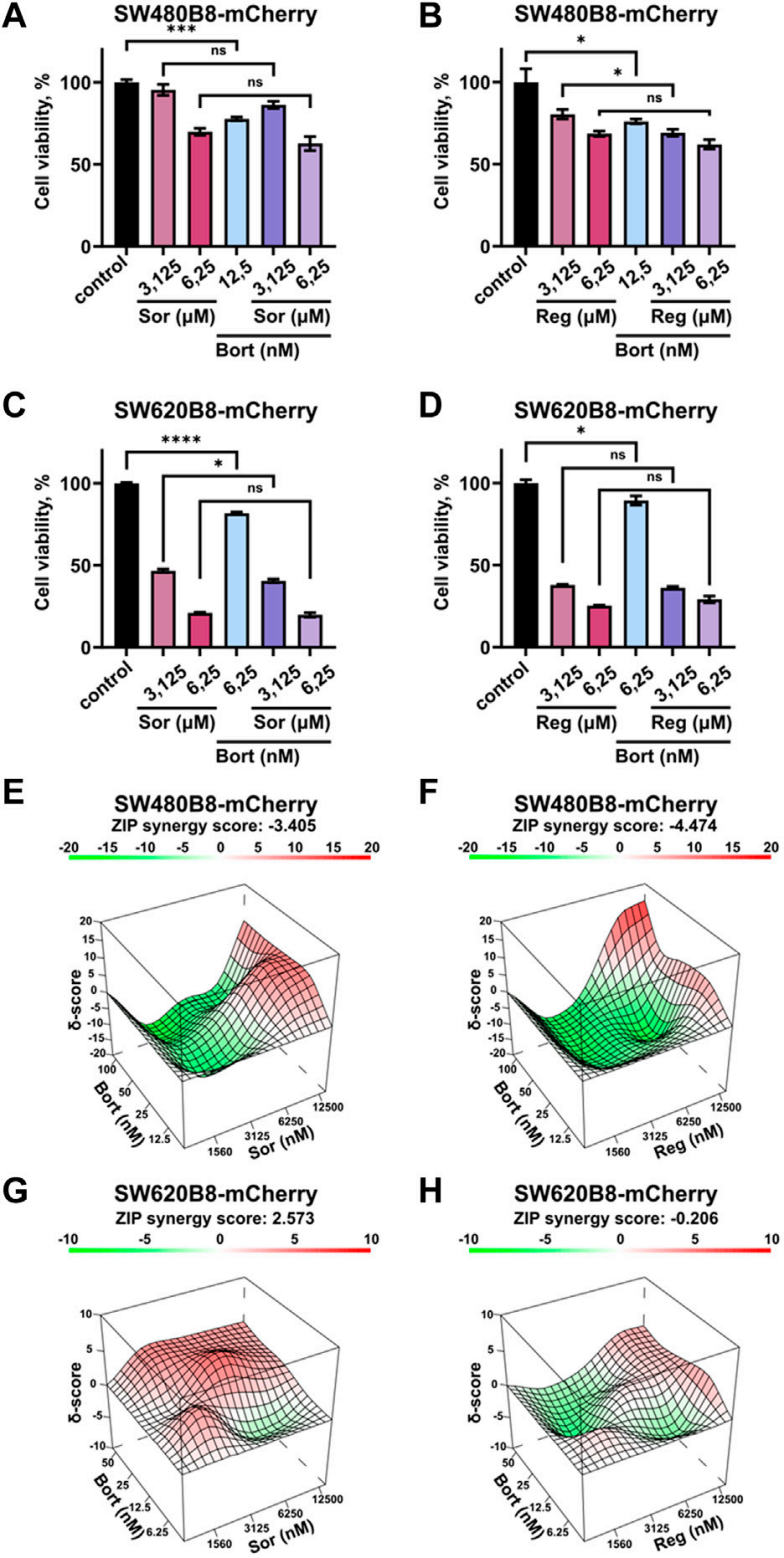
Sorafenib or regorafenib cause no synergistic or additive action when combined with bortezomib. The SW480B8-mCherry and SW620B8-mCherry cells were treated with bortezomib (Bort) in combination with sorafenib (Sor) or regorafenib (Reg). Viability was measured 72 h after treatment. **(A–D)** The bar-charts represent the viability of SW480B8-mCherry **(A, B)** and SW620B8-mCherry **(C, D)** cells treated with sorafenib/regorafenib in 2 concentrations and bortezomib alone or in combinations with the MKIs. The experiment was performed in three replicates. SEM is shown for each bar. p -value was determined by Unpaired t-test. Asterisks: ns -p>0.05, *- *p* < 0.05, **- *p* < 0.01, ***- *p* < 0.001, ****- *p* < 0.0001. **(E–H)** Synergy 3D plots represent the effect of drug combinations (synergism–red area; additive effect–white area; antagonism–green area) which was calculated and visualized using SynergyFinder 3.0 software.

Rearrangements within cellular proteasome pool can potentially affect the responsiveness of cancer cells to proteasome inhibitors. Thus, we studied the viability of cells treated with bortezomib in combination with sorafenib or regorafenib. Although all the inhibitors significantly affected the survival of cells when added separately, we did not found increase or even addition of cytotoxic effects when introduced sorafenib or regorafenib in combination with bortezomib (Figures 7A–D). As an example, treatment of SW480B8-mCherry cells with sorafenib added in low toxic concentration of 6.25 µM lead to 30% decrease of cell survival. Treatment of cells with low toxic concentration of bortezomib (12.5 nM) lead to approximately 20% decrease of cell survival. When used in combination no synergistic or even statistically significant additive actions on cell viability were detected (Figure 7A). Next, we studied effects of drug combinations taken in a broad range of concentrations on the viability of both cell lines. The dose-response matrixes were obtained and analyzed using Synergy Finder 3.0 software. The ZIP scores were calculated for combination of sorafenib with bortezomib (ZIP score: −3.405) and regorafenib with bortezomib (ZIP score: −4.474) when added to SW480B8-mCherry cells (Figures 7E, F). When sorafenib and regorafenib were added to SW620B8-mCherry cells in combination with bortezomib the ZIP scores were 2.573 and −0.206, respectively (Figures 7G, H). When ZIP score is more than 10 it may be interpreted as synergistic action, when less 10 and more than 0 as additive action, ZIP scores less than 0 – antagonistic action. Based on the obtained scores, it may be concluded that sorafenib or regorafenib cause most likely antagonistic action when combined with bortezomib.

## 4 Discussion

Multikinase inhibitors (MKIs) modulate signaling pathways that control survival and proliferation of cells and, therefore, widely used in the therapy of cancer. Regorafenib and sorafenib are among the most effective MKIs, utilized to treat solid tumors. Both inhibitors target different kinases including VEGFRs, PDGFRs, BRAF, RET and c-kit (Kannaiyan and Mahadevan, 2018). Structurally regorafenib and sorafenib are almost identical with the only difference–the presence of a fluorine atom in the central phenyl ring of regorafenib, which leads to certain differences in properties between the two compounds (Wilhelm et al., 2011). The regorafenib is approved for the treatment of metastatic colorectal carcinoma (Grothey et al., 2013), while sorafenib is mostly used for the treatment of hepatocellular carcinoma, renal cell carcinoma and differentiated thyroid cancer (Lee et al., 2023), however several publications indicate its applicability for the treatment of colorectal cancer (Kacan et al., 2016; Martchenko et al., 2016; Jeong et al., 2020). Importantly, MKIs were shown to induce immunomodulatory effects. For instance, previous studies demonstrated that exposure to MKIs including sorafenib and regorafenib can stimulate MHCI synthesis and expression of other components of the antigen presentation pathway in cancer cells and, consequently, stimulate their elimination by cytotoxic lymphocytes (Kwilas et al., 2014; Tsai et al., 2017; Takahashi et al., 2021). At the same time, little is known regarding the effect of MKIs on specifically the expression of non-constitutive proteasomes in cancer in general and in colorectal cancer in particular. Since immunoproteasomes and intermediate proteasomes substantially broad the repertoire of MHCI-presented peptides including those derived from cancer antigens (Vigneron et al., 2017), their expression is an essential parameter that can affect the outcome of the disease. Here, we found strong relation between BRAF mutations in colorectal cancer and expression of non-constitutive proteasomes. We demonstrated that regorafenib and sorafenib stimulate reactive oxygen species production, increase proteasome activity, upregulate expression and modulate non-constitutive proteasome localization in two colorectal cancer cell lines.

The clinical implication of the observed MKIs effects might be dichotomous. Indeed, upregulation of constitutive and non-constitutive proteasomes, which is frequently observed in cancer, could be both beneficial and detrimental for the tumor (Rouette et al., 2016; Leister et al., 2021; Chen et al., 2023).

From one side, as indicated above the expression of non-constitutive proteasomes may facilitate presentation of certain cancer antigens and stimulate elimination of tumor cells by the immune system (Vigneron et al., 2017). Concordantly, overexpression of immune proteasome subunits is a favorable prognostic marker for breast, endometrial and urothelial cancer, as well as non-small cell lung carcinoma (Rouette et al., 2016; Tripathi et al., 2016; Lee et al., 2019,^7^,^8^). Increased levels of immunoproteasomes in tumors might be associated with activation of their expression in cancer cells, or infiltration of the immune cells, or both. Importantly, non-constitutive proteasome expression is enhanced through secretion of IFN-γ by tumor-infiltrating lymphocytes. Indeed, considerably higher expression of immuno-proteasome subunits was shown in melanoma tissue infiltrated with CD3^+^ T-cells (Woods et al., 2016). IFN-γ-induced stimulation of MHC I and immunoproteasome subunit expression was in turn augmented by MKIs in hepatocellular carcinoma cells (Takahashi et al., 2021). Thus, treatment with such inhibitors affect generation and presentation of cancer antigens acting in a synergic manner with endogenous immune molecules and by this mean stimulate recognition of cancer cells by the immune system.

On the other hand, non-constitutive proteasomes are involved in degradation of tumor suppressor proteins (Chen et al., 2023). Along these lines, upregulation of *PSMB9* is an unfavorable prognostic marker in renal cancer.^9^ Moreover, immunoproteasomes promote production of pro-inflammatory cytokines (Basler et al., 2010) and their inhibition with non-constitutive proteasome inhibitor ONX-0914 was sufficient to suppress the growth of inflammation-induced colorectal cancer (Koerner et al., 2017; Vachharajani et al., 2017). Furthermore, in PSMB8-10KO mice no colitis-associated cancer development was observed (Leister et al., 2021), highlighting that non-constitutive proteasome gene expression may stimulate tumor growth via involvement in inflammatory response and likely in other more complex interactions of cancer cells with the immune system. Interestingly, one of the mechanisms of tumor cell adaptation to immune system pressure is associated with increased expression of programmed death-ligand 1 (PD-L1), a transmembrane protein known to suppress the activation of T-cells (Wang et al., 2016). Colorectal carcinomas with BRAF mutations show higher expression of PD-L1 (Srivastava et al., 2021), which can contribute to immune evasion of cancer cells and in turn nuclear PD-L1 promotes cell cycle progression of BRAF mutant cells (Ma et al., 2022). PD-L1 inhibition by sparatlizumab improved effectivity of combined inhibition of BRAF and MEK with dabrafenib and trametinib in phase 2 clinical trial for colorectal cancer (Tian et al., 2023). Along these lines, we calculated the correlation of proteasome gene expression with expression of PD-L1 in colorectal tumors. We found that *PSMB1-10* genes had significant correlation with PD-L1, however non-constitutive subunit genes *PSMB9*, *PSMB10* and *PSMB8* demonstrated the strongest Spearman correlations: R = 0.57, 0.43, 0.38, respectively and *p* < 0.0001 (Supplementary Figure S3). Moreover, we revealed modest upregulation of the *CD274* (encodes PD-L1) gene expression in cells treated with sorafenib (Supplementary Figure S4). Thus, activation of PD-L1 gene expression coincided with the activation of immunoproteasome subunit expression following treatment with the inhibitor. Here it should be mentioned that upregulated *PSMB8/9* expression correlated with increased efficacy of immune checkpoint inhibitors in melanoma (Kalaora et al., 2020) and lower-grade glioma (Liu et al., 2022). Interestingly, it has been shown that mutated forms of BRAF, NRAS, and KRAS can upregulate non-constitutive proteasome expression and reduce endoplasmic reticulum stress in multiple myeloma (Shirazi et al., 2020).

Moreover, cancer cells are constantly exposed to different stresses (Chen and Xie, 2018). Increased expression of non-constitutive proteasomes might help to deal with the consequences of stress-induced build-up of potentially toxic protein aggregates and facilitate their adaptation (Grune et al., 2011; Pickering et al., 2012; Johnston-Carey et al., 2015). Though we did not specifically address the mechanism that stands behind activation of immunoproteasome subunit expression following the incubation with the MKIs, it is well established that non-constitutive proteasome expression is increased in various stress conditions including oxidative stress (Johnston-Carey et al., 2015; Petersen and Zetterberg, 2016; Raynes et al., 2016; Wang et al., 2023). MKIs were demonstrated to induce various types of stress (Wang et al., 2023). Specifically, sorafenib was shown to induce oxidative stress, endoplasmic reticulum stress and inflammation (Rahmani et al., 2007; Wei et al., 2021; Wang et al., 2023). Regorafenib was also shown to induce endoplasmic reticulum stress (Sui et al., 2023), as well as the oxidative stress in colon cancer cells (Yu et al., 2023). Our results indicate that both drugs stimulate production of reactive oxygen species and hence, most likely—oxidative stress in studied colorectal cancer cell lines (Figures 6C, D). Thus, it could not be ruled out that stimulation of immunoproteasome subunit expression following treatment with the MKIs might involve stress-induced activation of relevant signaling pathways.

Another interesting consequence of different stresses is formation of intracellular membraneless inclusions containing proteasomes (Enenkel et al., 2022). Current findings indicate that most of these structures serve to sequester damaged proteins, facilitate their proteasomal degradation in order to cope with the consequence of stress and disappear when stress is relieved. Following treatment with MKIs, we observed re-localization of proteasomes. Upon exposure to regorafenib the distribution of non-constitutive proteasomes was not significantly altered in SW480B8-mCherry cells, but decreased cytoplasmic/nuclear ratio was observed in SW620B8-mCherry cells. However, especially in SW480B8-mCherry cells we revealed considerably higher proteasome activity in the nucleus comparing to the cytoplasm (Figure 6A). It has been shown that following different stresses proteasome-containing foci accumulate in the nucleus where proteolysis of unassembled orphan RPs and various ubiquitinated defective proteins that accumulate in stress conditions is performed (Yasuda et al., 2020; Fu et al., 2021; Uriarte et al., 2021). At least some of these structures were shown to contain p62 and heat-shock proteins and to stimulate degradation of defective proteins that accumulate following heat or oxidative stress (Fu et al., 2021). Thus, these assemblies seemingly perform protein quality control in the nucleus and their accumulation in the nucleus of regorafenib-treated cells might be a consequence of oxidative stress induced by this MKI. At the same time, following treatment with sorafenib, we revealed upregulation of proteasome activity in the nuclei but, surprisingly, non-constitutive proteasomes accumulated in the cytoplasm of both cell lines (Figure 6A). This indicates that in that case proteasome-containing foci and protein quality control mechanisms in the nuclei are likely mostly associated with constitutive proteasomes. We revealed optically dense structures containing active non-constitutive proteasomes in the area close to the nuclei of treated cells. Previously, sorafenib was shown to induce formation of stress granules (Adjibade et al., 2015; Dai et al., 2023). Formation of stress granules induced by sorafenib was suggested to promote cyclooxygenase-2 expression and survival of renal cancer cells (Dai et al., 2023). Though stress granules mainly contain RNA, ribosomal components and RNA-binding proteins, they were recently shown to participate in sequestration of misfolded proteins in the cytoplasm preventing their accumulation in the nucleus and protecting from perturbations in the nuclear proteostasis (Xu et al., 2023). Another study indicates that 26S proteasomes concentrate in perinuclear aggresomes that contain defective protein aggregates and facilitate autophagic clearance of these structures (Hao et al., 2013). Moreover, upon stress induced by proteasome inhibition, proteasomes and soluble ubiquitinated misfolded proteins aggregated in juxtanuclear compartment (JUNQ) (Kaganovich et al. 2008)

Recently, a Bcl2-associated athanogene 2 (BAG2) –containing phase-separated membraneless organelles were identified (Carrettiero et al., 2022). These structures were formed in response to by hyperosmotic, proteasome inhibition, temperature or oxidative stresses and except BAG2 were shown to contain heat-shock protein Hsp-70 and 20S proteasomes capped with 11S regulators (Carrettiero et al., 2022). These granules were rather small and demonstrated more or less equal distribution within the cytoplasm. Proteasome containing structures that we observed in sorafenib-treated cells resembled BAG2 granules, but also formed larger aggregates at the proximity to the nuclei. Since we did not specify other components of the aggregates we suppose that sorafenib may stimulate formation of various proteasome-containing assemblies including BAG2 granules, JUNQ-associated aggregates or perinuclear aggresomes. Moreover, these structures contain large amount of non-constitutive proteasomes. Different localization of constitutive and non-constitutive proteasomes in these cells indicates specialized role of non-constitutive proteasomes in adaptation to stress induced by sorafenib. Concordantly, a role of β5i-containing proteasomes in degradation of α-synuclein was proposed (Ugras et al., 2018). Induction of another immunoproteasome subunit β1i was recently shown to facilitate adaptation to mitochondrial stress and prevent formation of intracellular aggregates (Kim et al., 2023). In β5iKO mice, impaired proteostasis with accumulation of ubiquitinated proteins was revealed in microglia (Cetin et al., 2022). These findings are in line with the role of immunoproteasome in prevention of aggregate formation after stress (Seifert et al., 2010). Together, induction of non-constitutive proteasomes and proteasome-containing structures may represent an adaptation to oxidative stress induced by MKIs. At the same time, different localization of proteasomes following treatment with regorafenib or sorafenib indicates differences in response to the inhibitors.

It should be mentioned that the proteasome activity was significantly elevated in colorectal cells treated with both MKIs. Importantly, this was not associated with the increased amount of proteasomes within cells at least within the cytoplasm (Figure 4A). Except increase in number, the activity of proteasomes might be modulated by several factors: association with regulators, interactions with several proteins (other than classical regulators), capable to affect proteasome activity and post-translational modifications of proteasome/regulator subunits (Kors et al., 2019). It has been shown that tyrosine-kinase inhibitors prevent Src-dependent phosphorylation of the Rpt2 (19S regulator subunit) at Y439, affecting the association of the regulator with the 20S proteasome and hence, its activity (Chen et al., 2021). Although regorafenib and sorafenib do not directly target Src, the activation of the latter is induced by receptor tyrosine kinases (e.g., PDGFR, VEGFR), which are targets of selected MKIs. Another kinase that could be influenced by Src and can affect proteasome activity is the c-Abl. Thus, a c-Abl and Arg-kinase dependent phosphorylation of the structural proteasome subunit α4 at Y153 was shown to reduce the proteasome activity (Liu et al., 2006). Moreover, regorafenib and sorafenib target several kinases of the MAPK pathway. It has been shown that phosphorylation of Rpt5 subunit of the 19S proteasome regulator by the apoptosis-regulating kinase ASK1 a member of the MAPK family inhibited the ATPase and overall activity of the 26S proteasome (Um et al., 2010). Importantly, ASK1 is activated and negatively regulates the 26S proteasome in oxidative stress (Um et al., 2010). In stress conditions the phosphorylation of Y273 of another 19S regulator subunit Rpn2 was induced by p38 MAPK. This in turn reduced the proteasome activity and facilitated accumulation of polyubiquitinated proteins in cells (Lee et al., 2010). Accordingly, MAPK inhibitors were shown to stimulate proteasome activity (Lee et al., 2010) and interestingly, no increased abundance of proteasome subunits was detected. Moreover, silencing of other components of MAPK pathway: ASK1, MKK6 (MAP kinase kinase 6), as well as the p38 MAPK target protein MK2 also stimulated proteasome activity (Leestemaker et al., 2017). Thus, one cannot exclude that MKIs can stimulate the activity of proteasomes via modulation of post-translational modification pattern of 19S regulator and 20S proteasome subunits, facilitating the adaptation of colorectal cells to stress conditions induced by the inhibitors. Although this issue should be specifically addressed, it indicates that MKIs can affect the efficacy of proteasome inhibitors if used in combination. In previous publications sorafenib demonstrated a synergistic effect in combination with proteasome inhibitors against multiply myeloma (Ramakrishnan et al., 2010) and hepatocellular carcinoma (Honma et al., 2014). Therefore we tested whether the combinations of proteasome inhibitor (bortezomib) with either sorafenib or regorafenib might be effective against colorectal cancer cell lines. Using both cell lines it has been shown that sorafenib and regorafenib do not demonstrate synergy or additive effect with bortezomib. Moreover, certain combinations of MKIs and proteasome inhibitor revealed antagonistic effect (Figure 7). Obtained results indicate that stimulation of proteasome activity and rearrangement of proteasome pool induced by MKIs can affect cellular responsiveness to proteasome inhibition which might in turn affect the outcome of combined therapy.

Taken together, our results demonstrate that regorafenib and sorafenib modulate the activity and localization of proteasomes, as well as the expression of non-constitutive proteasomes in colorectal cancer cells. This might affect presentation of tumor antigens and could be associated with the adaptation of cancer cells to the oxidative stress induced by the inhibitors. Revealed phenomenon contributes to the understanding of immunomodulatory action of MKIs and mechanisms of the crosstalk between tumor and the immune system. At the same time, our results indicate that stimulation of proteasome activity with the MKIs can reduce the efficacy of proteasome inhibitors showing that specific tests are needed to determine the applicability of such combination for the therapy of a particular tumor. Finally, the non-constitutive proteasome expression and activity can be considered as potential markers for such therapy effectiveness.

## Data Availability

The original contributions presented in the study are included in the article/Supplementary Material, further inquiries can be directed to the corresponding author.

## Footnotes

1
https://r2.amc.nl/

2
https://depmap.org/portal/

3
https://portals.broadinstitute.org/ctrp.v2.1/

4
https://www.ebi.ac.uk/chembl/

5
https://imagej.nih.gov/ij/

6
https://synergyfinder.fimm.fi

7
https://www.proteinatlas.org/ENSG00000204264-PSMB8/pathology

8
https://www.proteinatlas.org/ENSG00000205220-PSMB10/pathology

9
https://www.proteinatlas.org/ENSG00000240065-PSMB9/pathology
