# Supplementary material for: Multikinase inhibitors modulate non-constitutive proteasome expression in colorectal cancer cells
Source: Front Mol Biosci. 2024 May 7;11:1351641. doi: 10.3389/fmolb.2024.1351641 (PMC11106389; doi:10.3389/fmolb.2024.1351641)
Supplement: Supplementary file 1 [file DataSheet1.docx]

Supplementary Material

**
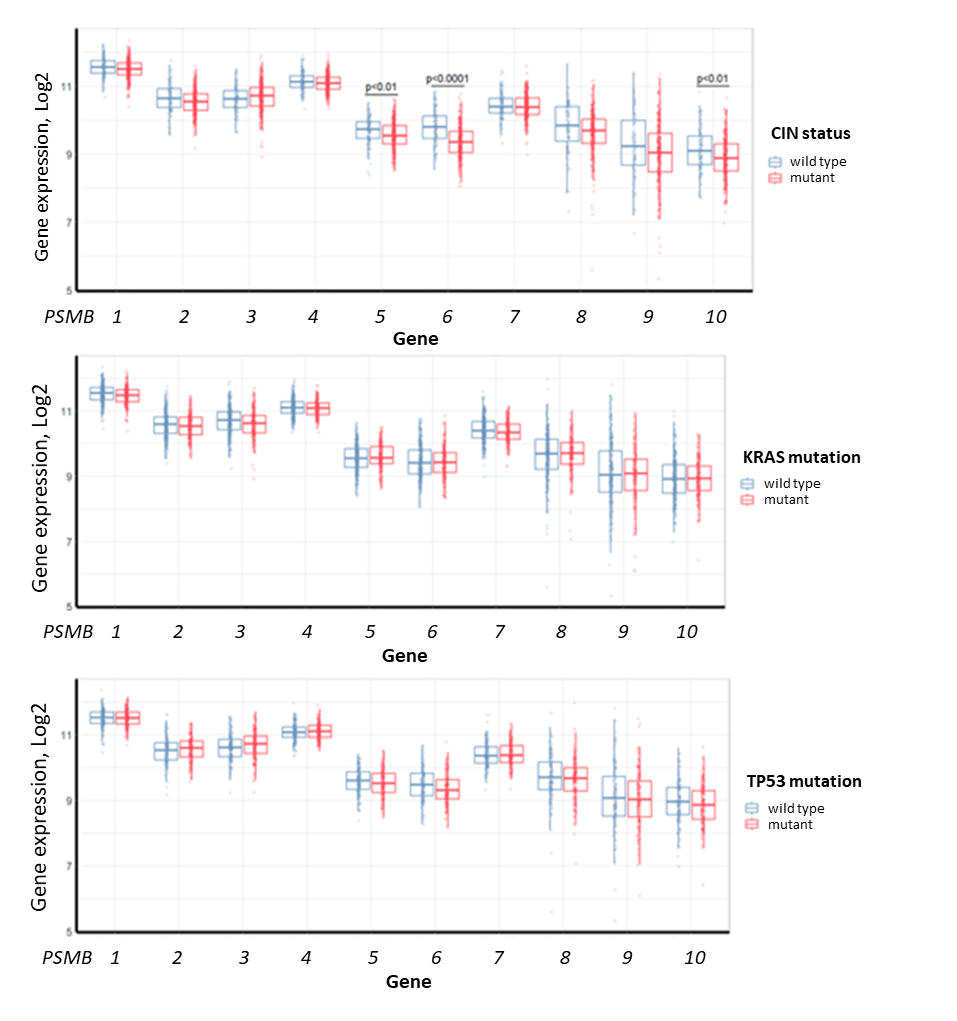
**

**Supplementary Figure 1.** Comparison of *PSMB1-10* genes expression in KRAS mutant, TP53 mutant tumors, and tumors with chromosomal instability (CIN) from GSE39582 dataset. Statistical significance was determined using Mann-Whitney non-parametric test with Benjamini-Hochberg correction.


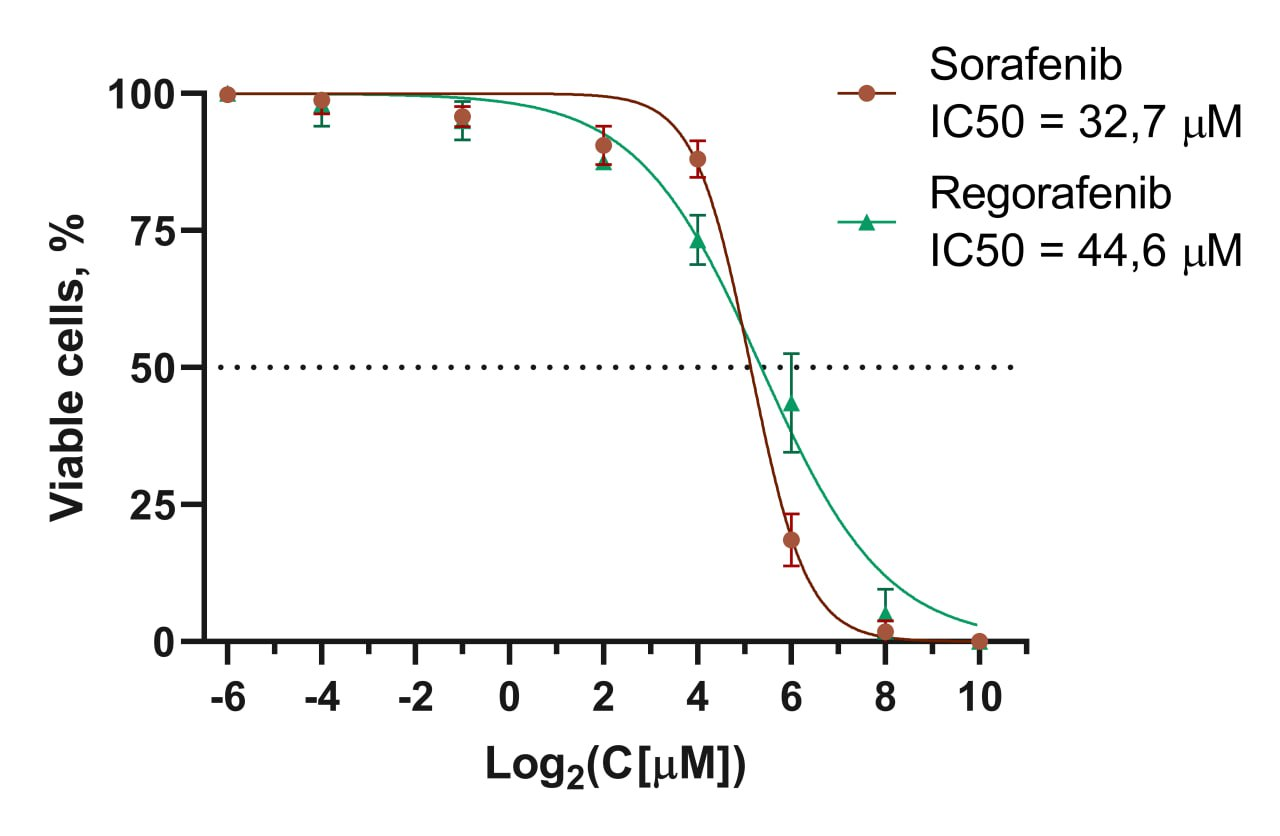


**Supplementary Figure 2.** Viability of HEK 293T cells treated with regorafenib or sorafenib. Сells were treated with 0.015–250 µM of regorafenib and 0.015–250 µM of sorafenib. Cellular viability was evaluated 72 h post drug-treatment using trypan-blue exclusion. Data represents average of SEM of three experiments.

**
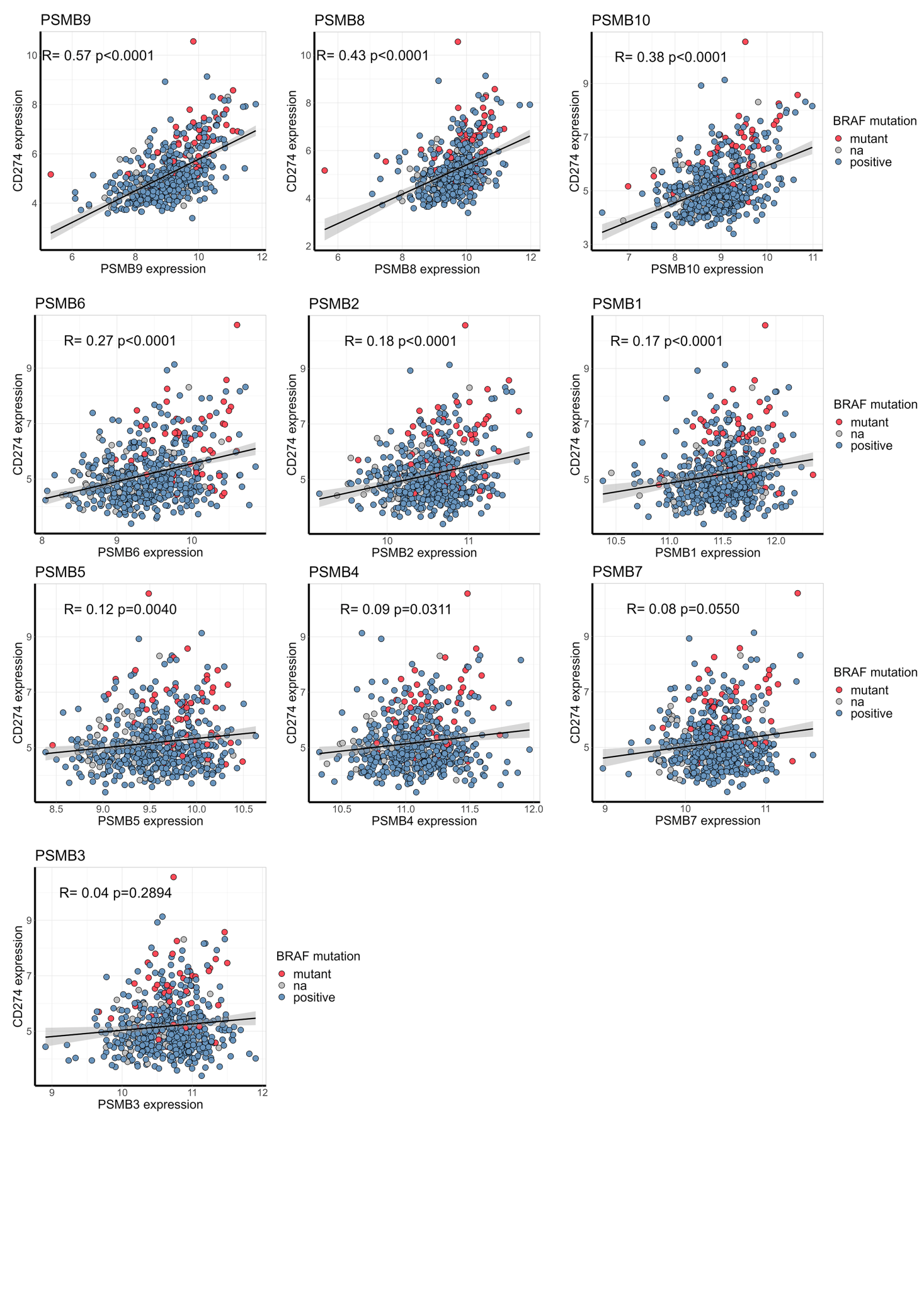
**

**Supplementary Figure 3**. Correlation of *CD274* and *PSMB9* gene expression in colorectal cancer tumors from GSE39582 dataset. Correlation was calculated using Spearman’s test.

**
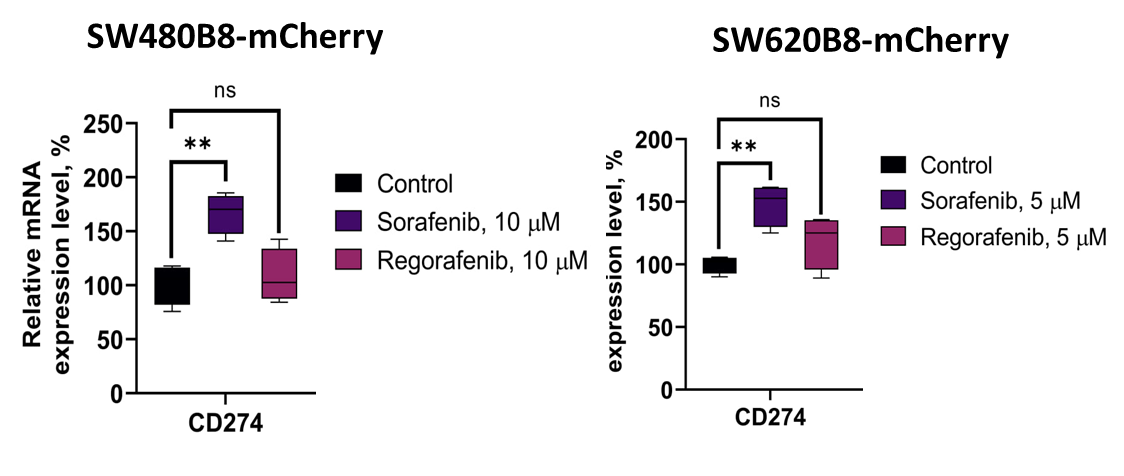
**

**Supplementary Figure 4.** The *CD274* gene expression levels in cells treated with the MKIs. The relative expression levels of *CD274* mRNA were determined by qPCR with primers shown in (Supplementary Table 2) after 72 h-long incubation with sorafenib or regorafenib. Ns- not significant; **—p < 0.01;***—p < 0.001;***—p < 0.001; ****—p < 0.0001, t-test.

**Supplementary Table 1.** Log2 protein level change of 20S proteasome subunits in HCT-116 cells treated with small molecule inhibitors. Data obtained from (Mitchell et al. 2023).

| **Compound** | **PSMB10** | **PSMB9** | **PSMB1** | **PSMB6** | **PSMB7** | **PSMB3** | **PSMB5** | **PSMB2** | **PSMB4** |
| --- | --- | --- | --- | --- | --- | --- | --- | --- | --- |
| **mek162** | 2,03 | 1,17 | 0 | 0,24 | 0,01 | 0,07 | 0,03 | 0 | 0,17 |
| **tcs_erk_11e** | 1,75 | 0,35 | -0,1 | 0,13 | -0,25 | 0,13 | 0,06 | -0,01 | 0,3 |
| **az_628** | 1,52 | 1,34 | 0,05 | 0,11 | -0,09 | 0,1 | 0,03 | 0,04 | 0,19 |
| **bafetinib** | 1,02 | 0,94 | 0,01 | 0,16 | 0,04 | 0,08 | 0,13 | -0,08 | 0,16 |
| **l-779450** | 0,99 | 1,17 | -0,08 | 0,12 | 0,02 | 0,05 | 0,11 | -0,21 | 0,1 |
| **encorafenib** | 0,89 | 0,67 | -0,1 | 0,16 | -0,15 | 0,05 | 0,05 | 0,06 | 0,15 |
| **bms-536924** | 0,88 | 0,55 | -0,06 | 0,09 | -0,09 | 0,07 | 0,13 | 0,01 | 0,1 |
| **mycophenolic_acid** | 0,82 | 0,6 | -0,02 | 0,06 | -0,01 | 0,09 | 0 | -0,07 | 0,1 |
| **nvp-bhg712** | 0,78 | 0,75 | -0,06 | 0,08 | -0,02 | -0,03 | -0,07 | 0,06 | -0,06 |
| **celastrol** | 0,74 |  | 0,14 | 0,1 | 0,03 | 0,2 | 0,1 | 0,06 | 0,28 |
| **cct128930** | 0,63 | 0,44 | 0 | 0,16 | -0,03 | 0 | 0,09 | 0,08 | 0,03 |
| **sunitinib** | 0,62 | 0,48 | 0,02 | 0,3 | 0,09 | 0,16 | 0,03 | -0,25 | 0,18 |
| **azd7762** | 0,62 | 0,25 | 0,03 | 0,18 | -0,07 | 0,12 | 0,09 | 0,08 | 0,06 |
| **epoxomicin** | 0,62 | -0,9 | 0,45 | -0,24 | 0,13 | 0,38 | -0,03 | 0,24 | 0,74 |
| **tppb** | 0,61 | 0,55 | 0,02 | 0,05 | 0,05 | 0,11 | 0,14 | 0,11 | 0,08 |
| **azd4547** | 0,59 | 0,08 | 0,14 | 0,1 | 0,1 | 0,04 | -0,05 | -0,07 | -0,04 |
| **unc2250** | 0,57 | 0,31 | 0,04 | 0,07 | -0,01 | 0,11 | 0,08 | 0 | 0,04 |
| **pp2** | 0,57 |  | -0,02 | 0,03 | -0,08 | 0,04 | -0,08 | -0,02 | 0,01 |
| **bosutinib** | 0,56 | 0,61 | -0,01 | 0,09 | 0,19 | 0,25 | 0,08 | -0,07 | 0,17 |
| **gsk2334470** | 0,56 | 0,08 | -0,04 | -0,04 | 0,06 | 0,05 | 0,02 | -0,07 | 0,09 |
| **nsc19883** | 0,56 | -0,02 | 0,02 | -0,07 | 0 | 0,01 | 0 | 0,03 | -0,01 |
| **reversine** | 0,53 |  | 0,04 | 0,15 | -0,01 | 0,08 | 0,1 | 0 | 0,08 |
| **radicicol** | 0,48 | 0,28 | 0,16 | 0,41 | 0,13 | 0,2 | 0,17 | 0,29 | 0,34 |
| **p005091** | 0,47 | 0,03 | 0,05 | -0,01 | 0 | 0,01 | -0,05 | -0,05 | -0,01 |
| **telotristat** | 0,46 | 0,24 | -0,01 | 0,18 | 0,01 | -0,01 | 0,01 | 0 | 0,01 |
| **midostaurin** | 0,45 |  | 0,06 | 0,09 | 0,03 | 0,13 | -0,01 | 0,02 | 0,13 |
| **azd8055** | 0,42 | 0,08 | -0,09 | 0,11 | -0,11 | 0,11 | -0,05 | -0,03 | 0,09 |
| **azd_6482** | 0,42 | 0,08 | 0,16 | 0,03 | -0,06 | 0,03 | -0,06 | -0,1 | -0,02 |
| **tae_226** | 0,42 | -0,01 | -0,07 | 0,11 | 0,01 | 0,04 | 0,05 | -0,19 | -0,01 |
| **anamorelin** | 0,42 | -0,08 | 0,05 | 0,03 | 0,03 | -0,08 | 0,05 | 0,04 | -0,04 |
| **nilotinib** | 0,42 |  | -0,05 | -0,03 | 0,03 | -0,03 | 0,04 | 0,02 | 0,06 |
| **dasatinib** | 0,41 | 0,04 | 0,03 | -0,03 | 0,04 | 0,18 | -0,05 | -0,09 | -0,03 |
| **pictilisib** | 0,4 | 0,01 | -0,06 | 0,12 | 0,01 | 0,08 | -0,07 | -0,04 | 0,07 |
| **wh-4-023** | 0,39 | 0,09 | 0,1 | 0,14 | 0,06 | 0,15 | 0,06 | -0,3 | 0,15 |
| **nvp-bep800** | 0,38 | 0,21 | 0,25 | 0,5 | 0,15 | 0,28 | 0,17 | 0,2 | 0,36 |
| **ch5132799** | 0,38 | -0,01 | -0,03 | 0,03 | -0,17 | 0,05 | 0 | -0,02 | 0,02 |
| **kpt-330** | 0,37 | 0,5 | 0,06 | 0,26 | 0,19 | 0,26 | 0,21 | 0,17 | 0,37 |
| **chembl2172374** | 0,37 | 0,02 | -0,02 | -0,05 | -0,04 | -0,02 | 0 | -0,02 | 0,01 |
| **amsacrine** | 0,37 | 0,02 | 0,08 | 0,12 | 0,08 | 0,15 | 0,12 | 0,09 | 0,18 |
| **nutlin3a** | 0,37 | -0,36 | 0,13 | 0,15 | 0,1 | 0,19 | 0,15 | -0,04 | 0,11 |
| **yk-4-279** | 0,36 | 0,08 | 0,17 | 0,18 | 0,05 | 0,2 | 0,1 | -0,01 | 0,28 |
| **vuf11207** | 0,35 | 0,19 | -0,05 | 0 | 0,07 | -0,01 | 0,05 | -0,04 | 0,21 |
| **migalastat** | 0,33 | 0,19 | 0,05 | -0,06 | -0,09 | -0,11 | 0,16 | 0 | 0,07 |
| **ingenol-3-angelate** | 0,33 | 0,16 | 0,06 | 0,02 | 0,08 | 0,07 | 0,03 | -0,01 | 0,06 |
| **simvastatin** | 0,33 | 0,08 | -0,05 | 0,04 | -0,04 | 0,04 | 0,05 | 0,05 | 0 |
| **a-317491** | 0,33 | 0,01 | 0,07 | -0,02 | 0,04 | 0,01 | -0,1 | 0,03 | -0,02 |
| **pd_160170** | 0,33 | -0,01 | 0,02 | 0,12 | -0,02 | 0,8 | 0,4 | 0,18 | 0,34 |
| **ziprasidone** | 0,31 | 0,08 | -0,05 | 0,06 | -0,04 | -0,22 | -0,11 | -0,1 | -0,04 |
| **ceritinib** | 0,29 | 0,51 | -0,13 | 0,04 | -0,08 | -0,07 | -0,08 | -0,14 | -0,09 |
| **bestatin** | 0,29 | 0,32 | -0,01 | -0,03 | 0,01 | 0,06 | -0,01 | -0,01 | 0,03 |
| **tozasertib** | 0,28 | 0,59 | -0,07 | 0,16 | 0 | 0,15 | 0,05 | -0,13 | 0,17 |
| **nu7441** | 0,28 | 0,47 | 0,06 | 0,12 | -0,02 | 0,13 | 0,08 | -0,1 | 0,23 |
| **roflumilast** | 0,28 | 0,2 | -0,01 | 0,01 | -0,07 | 0,01 | 0,03 | -0,03 | 0,01 |
| **fingolimod** | 0,28 | 0,13 | 0,22 | 0,05 | -0,07 | 0,14 | 0,01 | -0,11 | 0,02 |
| **rrd-251** | 0,28 | 0,03 | -0,02 | 0,03 | -0,01 | -0,02 | -0,03 | 0,09 | -0,09 |
| **upf_648** | 0,28 | -0,09 | -0,04 | 0,01 | -0,02 | -0,07 | 0,03 | 0,13 | 0 |
| **pd_176252** | 0,27 | 0,06 | 0,01 | 0,05 | 0,04 | 0,08 | 0,03 | -0,13 | 0,06 |
| **torcetrapib** | 0,26 | 0,35 | -0,12 | 0,01 | 0 | 0,19 | -0,1 | -0,2 | -0,08 |
| **bs-181** | 0,26 | 0,1 | 0 | -0,14 | 0,1 | 0,14 | 0,05 | -0,02 | 0,04 |
| **betamethasone** | 0,26 | 0 | 0,19 | -0,02 | -0,08 | -0,16 | -0,02 | -0,03 | -0,01 |
| **am_630** | 0,26 | -0,01 | -0,01 | 0 | 0,01 | 0,01 | -0,05 | 0,02 | 0,03 |
| **3-hydroxyflavone** | 0,26 | -0,06 | 0,01 | -0,05 | 0,04 | 0,08 | 0,05 | -0,05 | 0,05 |
| **timapiprant** | 0,26 | -0,07 | 0 | 0,02 | 0 | -0,04 | 0,02 | 0,03 | -0,05 |
| **stk066844** | 0,26 | -0,13 | 0,07 | 0,06 | 0,02 | 0,08 | 0,09 | 0 | 0,09 |
| **pac-1** | 0,26 | -0,19 | -0,06 | 0,06 | 0,06 | 0,01 | 0,07 | 0,02 | 0,01 |
| **cc-401** | 0,25 | 0,36 | 0,01 | 0,08 | 0,02 | 0,04 | 0,06 | -0,01 | 0,02 |
| **fumagillin** | 0,25 | 0,34 | 0,12 | 0,08 | 0,12 | 0,04 | 0,07 | 0,14 | 0,1 |
| **ku_55933** | 0,25 | 0,27 | 0,04 | -0,03 | -0,04 | -0,03 | -0,02 | -0,03 | 0,04 |
| **vidofludimus** | 0,25 | 0,08 | 0,02 | 0,13 | -0,04 | 0,06 | 0,1 | 0,04 | 0,14 |
| **osu-03012** | 0,25 |  | 0,05 | 0,15 | -0,04 | 0,07 | 0,03 | 0,11 | 0,08 |
| **stl336112** | 0,23 | 0,12 | 0 | 0,03 | 0,09 | 0,07 | 0,04 | -0,02 | 0,1 |
| **1_4-dhna** | 0,23 | 0,04 | 0,13 | 0,16 | 0,04 | 0,02 | 0,14 | 0,02 | 0,11 |
| **sc_560** | 0,23 | 0,01 | -0,01 | 0,08 | 0,02 | 0,08 | 0,03 | 0,05 | 0,04 |
| **way_316606** | 0,23 | 0,01 | -0,05 | -0,02 | -0,1 | 0,02 | 0,04 | 0,1 | -0,05 |
| **mg-132** | 0,23 | -0,08 | 0,55 | -0,15 | 0,55 | 0,38 | -0,07 | 0,07 | 0,48 |
| **duvelisib** | 0,22 | 0,49 | -0,01 | 0,01 | -0,03 | 0,04 | 0,01 | 0 | 0,04 |
| **forodesine** | 0,22 | 0,09 | -0,09 | 0,02 | 0,09 | -0,02 | -0,01 | 0 | 0 |
| **icotinib** | 0,22 | 0,03 | 0,06 | -0,04 | -0,01 | -0,04 | 0,04 | -0,21 | 0,03 |
| **am966** | 0,22 | -0,22 | 0,07 | -0,06 | 0,02 | 0,11 | 0,05 | 0,07 | 0,06 |
| **baricitinib** | 0,21 | 0,11 | -0,03 | 0,08 | 0,01 | 0 | 0,01 | -0,05 | 0,04 |
| **stl321288** | 0,21 | 0,02 | 0,06 | 0,07 | 0,1 | 0,08 | 0,04 | -0,05 | 0,03 |
| **momelotinib** | 0,21 | -0,45 | -0,04 | 0,12 | 0,17 | 0,21 | 0,05 | 0,06 | 0,15 |
| **tivozanib** | 0,2 | 0,24 | -0,04 | 0,08 | 0,08 | 0,02 | -0,01 | 0 | 0,02 |
| **km03430** | 0,2 | 0,15 | -0,01 | -0,03 | -0,03 | -0,17 | -0,05 | -0,04 | 0,04 |
| **turofexorate** | 0,2 | 0,12 | 0,05 | 0 | -0,04 | 0 | 0,04 | 0 | 0,03 |
| **cal-101** | 0,2 | 0,1 | 0,11 | -0,01 | -0,08 | 0 | -0,04 | -0,03 | -0,02 |
| **flavopiridol** | 0,2 | 0,02 | 0,17 | 0,26 | 0,05 | 0,36 | 0,18 | 0,24 | 0,26 |
| **tamibarotene** | 0,2 | 0,01 | 0,05 | -0,04 | -0,03 | -0,11 | -0,02 | -0,04 | -0,05 |
| **ribociclib** | 0,2 | -0,02 | 0,05 | 0,09 | 0,08 | 0,03 | 0,09 | 0,02 | 0,06 |
| **vu_0364739** | 0,19 | 0,25 | 0,01 | 0,12 | 0,17 | 0,1 | 0,01 | -0,17 | 0,15 |
| **cbio** | 0,19 | 0,13 | -0,09 | 0 | -0,07 | 0,03 | 0 | 0,02 | 0,07 |
| **sgi-1776** | 0,19 | -0,12 | -0,04 | -0,08 | 0,05 | 0,01 | -0,01 | -0,09 | 0 |
| **3-deazaneplanocin_a** | 0,18 | 0,15 | -0,11 | -0,1 | 0,16 | -0,01 | -0,01 | -0,07 | -0,08 |
| **4-benzylpyridine** | 0,18 | 0,03 | 0,01 | 0,03 | -0,08 | 0,04 | -0,02 | 0,02 | 0,04 |
| **l-menthol** | 0,18 | 0,02 | -0,04 | -0,04 | 0,01 | 0,04 | -0,01 | -0,01 | 0,03 |
| **mn-64** | 0,18 | 0,02 | -0,03 | 0,02 | -0,03 | -0,03 | -0,05 | -0,04 | 0 |
| **gbr_12909** | 0,18 | 0,01 | 0,13 | 0,22 | 0 | 0,11 | 0,06 | -0,11 | 0,1 |
| **xav_939** | 0,18 | 0 | 0,21 | 0,1 | 0,03 | -0,07 | 0,05 | 0,05 | 0,02 |
| **brl_54443** | 0,18 | -0,12 | -0,03 | 0 | -0,04 | 0,03 | -0,01 | -0,02 | 0,03 |
| **rk-24466** | 0,17 | 0,07 | 0,04 | -0,02 | -0,04 | -0,21 | -0,11 | -0,22 | 0,08 |
| **skf_89976a** | 0,17 | 0,03 | -0,05 | 0 | -0,03 | 0,1 | 0,04 | -0,01 | 0,06 |
| **hhantag** | 0,17 | -0,06 | 0,1 | 0,09 | 0,02 | -0,03 | -0,01 | 0,07 | -0,05 |
| **chembl1643306** | 0,17 | -0,28 | 0,04 | -0,02 | 0 | 0 | 0,06 | -0,02 | 0,06 |
| **r121919** | 0,17 |  | 0,03 | 0 | 0,06 | 0,01 | -0,05 | 0,02 | 0,01 |
| **imiquimod** | 0,16 | 0,02 | 0,09 | 0,05 | -0,04 | -0,05 | -0,01 | -0,03 | -0,03 |
| **jnj-38877605** | 0,16 | 0 | 0,04 | -0,06 | -0,1 | -0,08 | -0,06 | -0,12 | -0,04 |
| **(s)-selisistat** | 0,16 | -0,06 | 0,01 | 0,01 | -0,04 | -0,03 | -0,01 | 0 | 0,01 |
| **obatoclax** | 0,16 |  | -0,06 | 0,25 | 0,1 | 0,17 | 0,05 | 0,06 | 0,1 |
| **tc-n_22a** | 0,15 | 0,13 | 0,04 | -0,02 | -0,01 | -0,06 | -0,02 | -0,05 | -0,04 |
| **aq-390_43364023** | 0,15 | 0,11 | 0 | -0,08 | 0,05 | 0,01 | 0,06 | 0,04 | 0,04 |
| **milrinone** | 0,15 | 0,01 | 0,01 | -0,09 | -0,02 | 0,2 | 0,13 | 0,02 | 0,17 |
| **prx-08066** | 0,15 | 0,01 | 0,03 | -0,03 | 0,07 | 0,05 | 0 | 0,03 | -0,03 |
| **pf-431396** | 0,15 | 0,01 | 0,41 | 0,4 | 0,2 | 0,24 | 0,22 | 0,31 | 0,35 |
| **chembl2397099** | 0,15 | 0 | -0,03 | 0,06 | -0,1 | -0,05 | 0,01 | 0,01 | -0,06 |
| **genistein** | 0,15 | -0,01 | -0,05 | 0,04 | 0,01 | -0,03 | -0,05 | 0,02 | 0,02 |
| **pf-3758309** | 0,15 | -0,81 | 0,04 | 0,34 | -0,06 | 0,22 | 0,23 | 0,03 | 0,58 |
| **pf_4800567** | 0,14 | 0,4 | -0,02 | -0,03 | -0,05 | -0,06 | -0,04 | 0,07 | -0,03 |
| **sotrastaurin** | 0,14 | 0,15 | 0,1 | 0,03 | 0,02 | -0,01 | 0,25 | 0,01 | -0,07 |
| **pf-04620110** | 0,14 | 0,11 | -0,02 | -0,01 | 0,02 | -0,07 | 0,03 | 0,08 | 0,04 |
| **bx_912** | 0,14 | 0,07 | 0,03 | 0,06 | 0,33 | 0,18 | 0,02 | 0 | 0,05 |
| **am095** | 0,14 | -0,02 | 0,01 | -0,05 | -0,06 | -0,02 | -0,06 | 0,02 | 0,05 |
| **pdk1-in** | 0,14 | -0,05 | -0,1 | 0,08 | -0,02 | 0,02 | 0,01 | -0,07 | 0 |
| **gedatolisib** | 0,14 | -0,1 | -0,01 | 0,15 | -0,09 | 0,14 | 0,03 | -0,02 | 0,13 |
| **tropicamide** | 0,14 | -0,11 | -0,03 | -0,07 | 0,04 | 0 | 0,01 | -0,09 | 0,04 |
| **palbociclib** | 0,14 | -0,11 | -0,07 | 0 | -0,02 | 0,03 | 0,07 | 0,03 | 0,11 |
| **agn_190299** | 0,14 | -0,27 | -0,07 | 0,07 | -0,02 | -0,05 | 0,02 | 0,08 | -0,09 |
| **osi-027** | 0,13 | 0,23 | -0,13 | -0,02 | -0,06 | 0,06 | -0,02 | -0,1 | -0,04 |
| **hc_067047** | 0,13 | 0,17 | 0,08 | 0,01 | 0,03 | -0,03 | -0,04 | -0,06 | 0,01 |
| **enzalutamide** | 0,13 | 0,17 | -0,01 | 0 | 0,03 | 0,01 | 0,02 | 0,32 | 0 |
| **rimonabant** | 0,13 | 0,08 | -0,19 | 0 | 0,03 | 0,05 | 0,03 | -0,04 | -0,02 |
| **bms_303141** | 0,13 | 0,04 | -0,09 | -0,07 | -0,02 | 0,07 | -0,03 | -0,05 | 0,08 |
| **darifenacin** | 0,13 | 0,01 | -0,08 | -0,03 | 0 | -0,03 | 0 | 0,02 | -0,04 |
| **abt-199** | 0,13 | -0,02 | -0,04 | 0,04 | -0,1 | -0,03 | -0,06 | 0,05 | -0,04 |
| **abt-737** | 0,13 | -0,02 | 0,03 | -0,02 | -0,01 | 0 | 0,01 | -0,14 | 0,03 |
| **gk01981** | 0,13 | -0,09 | 0,03 | 0,01 | 0,09 | 0,09 | 0,03 | 0,05 | 0,07 |
| **zonisamide** | 0,13 | -0,14 | -0,11 | -0,03 | -0,07 | -0,03 | 0 | -0,08 | -0,02 |
| **prinomastat** | 0,13 | -0,35 | -0,02 | -0,04 | 0 | 0,04 | 0,02 | -0,04 | 0,02 |
| **bay_61-3606** | 0,13 |  | 0,2 | 0,18 | 0,1 | 0,13 | 0,05 | 0,15 | 0,14 |
| **fr_180204** | 0,12 | 0,2 | 0,06 | 0 | -0,05 | -0,09 | 0 | -0,06 | 0,02 |
| **zafirlukast** | 0,12 | 0,14 | 0 | 0,06 | 0 | 0,02 | -0,02 | 0,01 | -0,01 |
| **2_6-dichlorophenethylamine** | 0,12 | 0,14 | -0,04 | -0,03 | -0,03 | -0,07 | 0 | 0,04 | -0,03 |
| **dopamine** | 0,12 | 0,09 | -0,03 | -0,05 | -0,08 | -0,09 | -0,06 | -0,05 | -0,07 |
| **ispinesib** | 0,12 | 0,08 | 0,04 | 0,24 | 0,03 | 0,13 | 0,12 | 0,21 | 0,04 |
| **aprepitant** | 0,12 | 0,05 | -0,07 | -0,11 | 0,01 | -0,06 | -0,04 | -0,06 | -0,05 |
| **purvalanol** | 0,12 | 0,03 | 0,05 | -0,03 | 0,02 | 0 | -0,06 | 0 | 0,01 |
| **pepstatin_a** | 0,12 | 0 | 0,07 | 0,04 | 0,09 | -0,01 | 0,04 | 0,06 | 0,13 |
| **ml221** | 0,12 | -0,02 | -0,06 | -0,04 | -0,09 | -0,32 | -0,1 | -0,1 | 0,06 |
| **formestane** | 0,12 | -0,07 | -0,01 | -0,02 | -0,01 | -0,17 | 0 | 0,06 | -0,07 |
| **iwr-1** | 0,12 | -0,07 | 0,02 | -0,04 | 0,02 | 0,01 | -0,01 | 0,01 | -0,01 |
| **a-naphthoflavone** | 0,12 | -0,1 | -0,03 | -0,08 | 0,02 | 0,01 | -0,01 | 0 | -0,04 |
| **ivabradine** | 0,12 | -0,1 | -0,02 | 0,01 | 0,01 | -0,01 | 0 | 0,09 | -0,01 |
| **fmk** | 0,12 | -0,11 | 0,03 | 0,16 | 0,08 | 0,03 | 0,04 | 0,06 | 0,14 |
| **zosuquidar** | 0,12 | -0,42 | 0,07 | -0,02 | -0,02 | 0,07 | 0 | 0,02 | 0,03 |
| **nibr189** | 0,11 | 0,3 | -0,01 | 0,11 | -0,02 | -0,03 | 0,03 | 0,01 | 0,01 |
| **taranabant** | 0,11 | 0,21 | 0,01 | 0,06 | 0,01 | 0,07 | 0,02 | 0,04 | 0,07 |
| **ticagrelor** | 0,11 | 0,2 | 0,01 | -0,02 | 0,03 | -0,01 | 0,01 | 0 | 0,02 |
| **ns309** | 0,11 | 0,16 | 0 | 0,03 | -0,01 | -0,05 | -0,03 | -0,08 | 0 |
| **(s)-(-)-pindolol** | 0,11 | 0,11 | 0 | 0,08 | 0,01 | 0,04 | 0,1 | 0,04 | 0,02 |
| **quiflapon** | 0,11 | 0,11 | 0,02 | -0,06 | -0,07 | -0,02 | 0 | 0,01 | 0,04 |
| **ndt_9513727** | 0,11 | 0,09 | 0,03 | -0,04 | -0,03 | 0,08 | 0,04 | 0,07 | 0,04 |
| **org25543** | 0,11 | 0,07 | -0,04 | 0,03 | 0,02 | -0,01 | 0,05 | -0,06 | 0,04 |
| **sb-674042** | 0,11 | 0,06 | 0,03 | -0,06 | -0,06 | -0,07 | 0 | -0,15 | -0,06 |
| **amn_082** | 0,11 | 0,01 | 0,06 | 0,06 | 0,16 | 0,03 | -0,01 | 0,02 | 0,04 |
| **fluconazole** | 0,11 | 0,01 | 0,01 | 0,02 | -0,07 | 0,02 | -0,01 | 0,03 | 0,02 |
| **tak-285** | 0,11 | 0 | -0,14 | 0 | 0,01 | -0,04 | -0,05 | 0,11 | 0,04 |
| **pf-543** | 0,11 | -0,08 | -0,17 | 0,02 | 0,02 | -0,17 | -0,01 | -0,15 | 0,04 |
| **lalistat1** | 0,11 | -0,11 | -0,02 | -0,02 | 0,04 | 0,01 | 0,05 | -0,01 | -0,04 |
| **chembl1902935** | 0,11 | -0,11 | 0,02 | 0,04 | 0,03 | -0,02 | 0,08 | 0,06 | 0,03 |
| **spb06189** | 0,11 | -0,12 | -0,06 | -0,11 | 0,02 | 0,02 | 0 | 0 | 0,1 |
| **dichlorphenamide** | 0,11 | -0,14 | 0,01 | 0,07 | -0,01 | 0,02 | -0,04 | -0,08 | 0 |
| **silmitasertib** | 0,11 | -0,22 | 0,04 | 0,03 | 0,01 | 0,15 | 0,02 | -0,01 | 0,09 |
| **sar7334** | 0,11 |  | -0,07 | 0,06 | -0,06 | -0,03 | 0,04 | -0,04 | 0,03 |
| **cediranib** | 0,1 | 0,1 | -0,01 | 0,07 | -0,01 | 0,04 | 0,07 | 0,01 | 0,04 |
| **as-605240** | 0,1 | 0,07 | 0,01 | 0,01 | -0,06 | -0,07 | 0,04 | 0,04 | -0,01 |
| **rhodanine_derivative38** | 0,1 | 0,04 | 0,02 | 0,04 | -0,04 | 0,04 | 0,02 | -0,06 | 0,02 |
| **tazarotene2** | 0,1 | 0,04 | 0,02 | -0,04 | -0,03 | -0,03 | -0,05 | 0,02 | 0,01 |
| **benzo[a]pyrene** | 0,1 | 0,03 | 0,01 | 0,07 | -0,05 | 0,07 | -0,04 | -0,15 | 0,05 |
| **dorzolamide** | 0,1 | 0 | 0,05 | 0,05 | 0,02 | 0,04 | -0,02 | 0,03 | 0 |
| **glufosinate** | 0,1 | -0,01 | 0,12 | -0,08 | -0,01 | 0,07 | -0,02 | 0,05 | -0,05 |
| **entospletinib** | 0,1 | -0,03 | 0,24 | 0,05 | -0,02 | 0,02 | 0,03 | -0,16 | 0 |
| **merck544** | 0,1 | -0,04 | -0,07 | 0 | 0,02 | -0,1 | 0,03 | 0 | -0,04 |
| **abemaciclib** | 0,1 | -0,04 | 0 | 0,18 | 0,04 | 0,14 | 0,11 | 0,04 | 0,14 |
| **alvimopan** | 0,1 | -0,12 | 0,04 | 0,01 | -0,08 | -0,05 | -0,01 | -0,05 | -0,06 |
| **olaparib** | 0,09 | 0,24 | -0,01 | 0,03 | 0,04 | 0,03 | 0,07 | -0,03 | -0,02 |
| **zotarolimus** | 0,09 | 0,19 | 0,06 | 0,13 | 0,05 | 0,03 | 0,05 | 0,03 | 0,11 |
| **sultiame** | 0,09 | 0,18 | -0,03 | -0,04 | -0,01 | -0,06 | 0,01 | -0,09 | -0,01 |
| **ps-1145** | 0,09 | 0,15 | -0,08 | 0 | 0 | 0,04 | -0,01 | -0,01 | -0,08 |
| **odanacatib** | 0,09 | 0,13 | -0,01 | 0,06 | 0 | -0,07 | 0,03 | 0 | 0,01 |
| **sgc0946** | 0,09 | 0,09 | -0,03 | 0,02 | -0,01 | -0,1 | 0 | 0,11 | 0,05 |
| **ramelteon** | 0,09 | 0,06 | -0,04 | 0,01 | -0,02 | -0,02 | 0,04 | -0,03 | -0,02 |
| **bms-911543** | 0,09 | 0,05 | 0,04 | -0,02 | -0,02 | -0,03 | -0,03 | 0,03 | -0,02 |
| **xen445** | 0,09 | 0,05 | 0,03 | 0,04 | -0,04 | -0,02 | 0,04 | 0 | -0,04 |
| **sr_27897** | 0,09 | -0,02 | -0,01 | -0,01 | -0,02 | -0,08 | -0,03 | -0,01 | -0,04 |
| **mildronate** | 0,09 | -0,05 | -0,01 | 0 | 0 | -0,01 | 0,01 | -0,08 | 0,01 |
| **nfps** | 0,09 | -0,05 | 0,03 | 0,03 | -0,04 | -0,01 | -0,02 | 0,02 | 0 |
| **minocycline** | 0,09 |  | -0,03 | -0,03 | 0 | 0,02 | 0,03 | -0,01 | -0,05 |
| **jte_013** | 0,08 | 0,31 | -0,02 | 0,01 | -0,02 | -0,03 | -0,04 | -0,05 | 0 |
| **toceranib** | 0,08 | 0,29 | -0,03 | 0 | 0,01 | 0,02 | -0,01 | -0,06 | 0,01 |
| **az505** | 0,08 | 0,19 | -0,05 | 0,02 | -0,08 | 0 | 0 | 0,03 | -0,07 |
| **pd173212** | 0,08 | 0,13 | -0,09 | 0,06 | 0,01 | -0,05 | -0,07 | -0,06 | 0 |
| **sulfanilamide** | 0,08 | -0,02 | 0,05 | 0,08 | 0,04 | 0,02 | 0,03 | 0,06 | -0,01 |
| **sumatriptan** | 0,08 | -0,05 | 0,07 | -0,07 | -0,06 | 0 | 0,01 | -0,05 | -0,09 |
| **alogliptin** | 0,08 | -0,07 | 0,06 | -0,01 | -0,05 | -0,02 | -0,06 | -0,06 | -0,08 |
| **ospemifene** | 0,08 | -0,08 | 0,01 | 0,01 | 0,01 | -0,03 | -0,04 | -0,08 | -0,05 |
| **leupeptin** | 0,08 | -0,12 | -0,07 | -0,05 | 0,03 | -0,05 | -0,09 | -0,01 | -0,02 |
| **gliclazide** | 0,08 | -0,13 | -0,04 | 0,04 | 0,01 | -0,03 | -0,03 | 0,01 | 0,03 |
| **pf429242** | 0,08 |  | 0,13 | 0 | -0,09 | -0,05 | 0,17 | 0 | -0,07 |
| **thiamet_g** | 0,08 |  | 0,04 | 0,03 | -0,05 | -0,02 | -0,03 | -0,01 | -0,04 |
| **fti-277** | 0,08 |  | 0,05 | 0 | 0,07 | 0,03 | 0,05 | 0,03 | 0,06 |
| **perhexiline** | 0,08 |  | -0,01 | 0,07 | -0,08 | 0,16 | -0,05 | 0,1 | 0,05 |
| **gs-9620** | 0,07 | 0,16 | -0,01 | 0,01 | -0,02 | 0,03 | -0,02 | -0,11 | 0 |
| **succinobucol** | 0,07 | 0,14 | -0,03 | -0,02 | 0,02 | 0,08 | -0,04 | 0,03 | 0,03 |
| **r-848** | 0,07 | 0,13 | 0,02 | -0,05 | -0,06 | -0,07 | -0,01 | -0,04 | -0,09 |
| **treprostinil** | 0,07 | 0,1 | 0,03 | -0,06 | -0,06 | 0,03 | -0,02 | 0,04 | -0,01 |
| **thymidine** | 0,07 | 0,08 | 0,03 | 0,02 | 0,04 | -0,06 | 0,05 | -0,01 | 0,01 |
| **asarone** | 0,07 | 0 | 0,02 | -0,02 | 0,02 | 0,05 | -0,03 | 0 | 0,03 |
| **stk416816** | 0,07 | -0,03 | -0,05 | -0,03 | -0,05 | 0,01 | -0,02 | 0,01 | -0,04 |
| **tc-o_9311** | 0,07 | -0,05 | -0,02 | -0,05 | 0,09 | 0,01 | -0,1 | 0 | 0 |
| **ro-3306** | 0,07 | -0,07 | -0,01 | 0,11 | 0,12 | 0,04 | 0,09 | -0,03 | 0,07 |
| **umi-77** | 0,07 | -0,08 | 0,22 | 0,03 | -0,01 | 0,05 | 0,02 | -0,04 | 0 |
| **filanesib** | 0,07 | -0,09 | 0,09 | 0,13 | 0,18 | 0,29 | 0,01 | -0,04 | 0,15 |
| **topiramate** | 0,07 | -0,11 | 0,01 | -0,02 | 0,02 | 0,05 | 0 | -0,04 | 0,02 |
| **dmb** | 0,07 | -0,28 | -0,04 | -0,04 | -0,03 | -0,09 | -0,02 | -0,07 | -0,08 |
| **boc-4mesyloxypiperidine** | 0,07 | -0,31 | -0,09 | -0,08 | 0 | -0,1 | -0,08 | -0,06 | -0,02 |
| **sp600125** | 0,07 |  | 0,03 | -0,01 | 0,01 | 0,03 | 0,01 | 0,02 | -0,05 |
| **iso-1** | 0,06 | 0,28 | -0,07 | -0,03 | -0,05 | 0,03 | -0,05 | 0,01 | 0,06 |
| **pomalidomide** | 0,06 | 0,09 | 0,09 | 0 | -0,03 | 0,01 | 0 | -0,1 | 0,01 |
| **meloxicam** | 0,06 | 0 | 0,04 | -0,06 | -0,06 | -0,06 | -0,02 | -0,02 | -0,06 |
| **ginkgolide_a** | 0,06 | -0,03 | -0,01 | -0,05 | -0,06 | 0 | 0 | -0,02 | 0,02 |
| **carvedilol** | 0,06 | -0,04 | -0,02 | -0,03 | 0,02 | 0,03 | -0,04 | 0,05 | -0,07 |
| **4-aminobenzohydrazide** | 0,06 | -0,27 | -0,16 | -0,15 | 0,04 | 0,01 | 0,01 | 0,07 | 0,11 |
| **9-aminocamptothecin** | 0,06 | -0,4 | 0,11 | 0,38 | -0,1 | 0,18 | 0,18 | 0,16 | 0,29 |
| **crizotinib** | 0,05 | 0,32 | -0,12 | 0,11 | -0,01 | 0 | -0,03 | 0,15 | 0 |
| **talazoparib** | 0,05 | 0,17 | -0,01 | 0,04 | 0,03 | -0,04 | 0,02 | 0 | 0 |
| **rn486** | 0,05 | 0,1 | -0,01 | 0,06 | 0,04 | 0,06 | 0,1 | -0,02 | 0,1 |
| **uamc00039** | 0,05 | 0,01 | 0,02 | 0,04 | 0,02 | -0,03 | -0,01 | 0 | 0,03 |
| **azilsartan** | 0,05 | 0,01 | 0 | -0,06 | -0,04 | -0,01 | -0,04 | 0,02 | -0,02 |
| **lasmiditan** | 0,05 | -0,02 | 0 | -0,03 | -0,04 | -0,01 | -0,05 | 0,03 | -0,05 |
| **ar-c155858** | 0,05 | -0,07 | 0,01 | -0,03 | -0,02 | 0,01 | -0,04 | 0,01 | 0 |
| **teijin_compound_1** | 0,05 | -0,08 | 0 | -0,08 | -0,04 | 0,01 | 0,05 | 0,07 | -0,06 |
| **lp-533401** | 0,05 | -0,08 | 0,13 | -0,02 | -0,03 | 0,06 | 0,13 | -0,08 | -0,02 |
| **ozagrel** | 0,05 | -0,09 | 0,29 | -0,02 | -0,11 | -0,05 | 0 | 0,05 | 0,03 |
| **spironolactone** | 0,05 | -0,1 | -0,01 | -0,05 | -0,09 | -0,04 | 0,01 | -0,03 | -0,04 |
| **t-00127** | 0,05 | -0,11 | -0,02 | -0,02 | -0,02 | 0,03 | -0,02 | 0,01 | 0 |
| **lixivaptan** | 0,05 | -0,11 | -0,03 | -0,01 | 0 | -0,02 | -0,03 | 0,09 | -0,01 |
| **arl_17477** | 0,05 | -0,13 | 0,1 | -0,08 | 0,02 | -0,12 | -0,04 | 0,01 | -0,14 |
| **l755507** | 0,05 | -0,15 | -0,01 | 0,02 | 0,05 | -0,05 | -0,02 | -0,05 | 0 |
| **vu0152100** | 0,05 | -0,16 | 0,08 | 0 | 0,01 | 0 | -0,03 | -0,04 | -0,06 |
| **silodosin** | 0,05 | -0,19 | -0,03 | 0,05 | 0,04 | -0,02 | 0,02 | -0,06 | -0,01 |
| **asimadoline** | 0,04 | 0,21 | -0,07 | -0,01 | 0,06 | -0,16 | -0,02 | -0,09 | 0,03 |
| **paroxetine** | 0,04 | 0,18 | -0,02 | -0,03 | -0,03 | -0,07 | 0,07 | -0,03 | -0,06 |
| **sar020106** | 0,04 | 0,11 | -0,02 | -0,13 | 0,01 | 0,02 | 0,01 | -0,03 | 0,01 |
| **binospirone** | 0,04 | 0,11 | -0,03 | 0,02 | -0,09 | -0,1 | -0,07 | 0,12 | -0,1 |
| **climbazole** | 0,04 | 0,1 | -0,05 | -0,03 | -0,01 | -0,01 | -0,02 | 0,04 | -0,05 |
| **tiagabine** | 0,04 | 0,07 | 0,18 | 0,01 | -0,09 | 0,06 | -0,01 | -0,09 | -0,02 |
| **ly2584702** | 0,04 | 0,06 | 0,04 | -0,01 | 0,03 | 0,01 | 0,03 | 0,08 | 0,05 |
| **pf-514273** | 0,04 | 0,05 | -0,03 | 0 | 0,01 | 0,01 | -0,03 | 0,05 | -0,02 |
| **rf9** | 0,04 | 0,05 | 0,06 | -0,02 | -0,04 | 0,04 | -0,03 | 0 | 0 |
| **conivaptan** | 0,04 | 0,03 | -0,04 | 0,07 | -0,05 | -0,02 | -0,01 | 0,05 | 0,01 |
| **dicyclohexylamine** | 0,04 | 0,02 | 0,05 | 0 | 0,01 | -0,03 | -0,01 | 0,04 | 0,01 |
| **actonel** | 0,04 | 0,01 | -0,01 | -0,01 | -0,03 | 0,03 | -0,1 | 0,03 | -0,03 |
| **torasemide** | 0,04 | 0,01 | 0,02 | -0,02 | -0,01 | -0,03 | -0,06 | 0 | -0,01 |
| **dicumarol** | 0,04 | -0,02 | 0 | -0,02 | -0,02 | -0,12 | -0,03 | 0,09 | 0,02 |
| **iloprost** | 0,04 | -0,02 | -0,03 | -0,01 | -0,05 | 0 | -0,06 | -0,12 | -0,04 |
| **sr_11237** | 0,04 | -0,08 | 0,02 | 0 | -0,08 | -0,01 | 0,03 | -0,03 | 0 |
| **chembl570596** | 0,04 | -0,1 | -0,03 | -0,01 | 0,09 | 0 | 0 | 0,04 | 0 |
| **bay_57-1293** | 0,04 | -0,11 | 0,19 | 0,03 | -0,12 | 0,01 | -0,02 | -0,06 | -0,07 |
| **stk155799** | 0,04 | -0,11 | 0 | -0,03 | -0,04 | 0,05 | -0,04 | 0,04 | -0,01 |
| **spautin-1** | 0,04 | -0,13 | -0,01 | -0,02 | 0,03 | -0,03 | 0 | 0,12 | -0,09 |
| **zebularine** | 0,04 | -0,14 | 0,09 | -0,06 | 0,01 | 0,02 | 0 | -0,09 | -0,04 |
| **tetrindole** | 0,04 | -0,16 | 0 | 0,1 | 0,04 | 0,1 | 0,04 | -0,13 | 0,07 |
| **olcegepant** | 0,04 | -0,22 | -0,04 | -0,08 | 0 | 0 | 0,02 | 0,03 | -0,04 |
| **4-methoxybenzoic_acid** | 0,04 |  | -0,03 | -0,05 | 0,04 | 0,01 | -0,08 | -0,06 | 0,04 |
| **tipifarnib** | 0,03 | 0,32 | -0,04 | 0,01 | 0,01 | 0,05 | -0,01 | -0,12 | 0,04 |
| **(4-chlorobenzyl)pyridine** | 0,03 | 0,12 | -0,16 | 0,06 | -0,1 | 0,04 | -0,02 | -0,16 | -0,1 |
| **saracatinib** | 0,03 | 0,11 | -0,01 | 0,1 | -0,02 | 0,02 | 0,05 | -0,02 | 0,1 |
| **phenylboronic_acid** | 0,03 | 0,09 | 0,02 | 0,02 | -0,03 | -0,04 | -0,07 | 0,05 | 0 |
| **avosentan** | 0,03 | 0,08 | 0,02 | -0,01 | 0,03 | 0 | 0,04 | -0,03 | 0,03 |
| **ml-323** | 0,03 | 0,08 | -0,1 | 0,1 | 0,04 | -0,14 | 0,01 | -0,19 | 0,18 |
| **ko143** | 0,03 | 0,07 | -0,03 | -0,01 | -0,03 | -0,07 | 0,02 | 0,14 | -0,07 |
| **actinonin** | 0,03 | 0,04 | -0,04 | -0,01 | -0,04 | 0,03 | 0,03 | -0,02 | -0,07 |
| **evacetrapib** | 0,03 | 0,04 | -0,07 | -0,06 | 0,01 | -0,02 | -0,03 | -0,04 | -0,05 |
| **levosimendan** | 0,03 | 0,02 | -0,01 | 0,04 | 0,03 | -0,01 | -0,01 | 0,15 | -0,02 |
| **alosetron** | 0,03 | 0,01 | 0,06 | 0 | 0,02 | 0,03 | -0,01 | 0,07 | 0,02 |
| **nateglinide** | 0,03 | 0,01 | 0,15 | -0,06 | -0,07 | -0,05 | 0,03 | -0,18 | -0,12 |
| **lorcaserin** | 0,03 | 0,01 | 0,14 | -0,08 | -0,14 | -0,04 | 0,03 | -0,05 | -0,08 |
| **nsc_87877** | 0,03 | -0,03 | 0,01 | -0,02 | -0,02 | -0,03 | -0,03 | -0,02 | -0,01 |
| **pj34** | 0,03 | -0,03 | 0,01 | 0,02 | -0,06 | -0,08 | 0,06 | -0,04 | -0,06 |
| **moxonidine** | 0,03 | -0,09 | 0,02 | 0,01 | 0,04 | -0,03 | 0 | 0,02 | -0,01 |
| **tolvaptan** | 0,03 | -0,15 | 0,1 | -0,03 | -0,01 | 0,01 | 0,03 | -0,05 | -0,02 |
| **6603-0104** | 0,03 | -0,18 | 0 | -0,05 | 0 | -0,01 | -0,03 | 0,08 | 0,04 |
| **dexrazoxane** | 0,03 | -0,24 | 0,06 | 0 | 0,02 | -0,02 | 0,08 | -0,01 | 0,01 |
| **vu_0364439** | 0,03 | -0,26 | 0,02 | -0,04 | 0,03 | 0,03 | 0,04 | -0,03 | 0,02 |
| **ldn-192960** | 0,03 | -0,36 | -0,1 | 0,07 | 0,02 | 0,05 | 0,09 | 0,08 | 0,08 |
| **sulforaphane** | 0,03 |  | -0,05 | -0,07 | -0,05 | -0,03 | -0,01 | -0,04 | 0,05 |
| **sb_699551** | 0,02 | 0,19 | -0,02 | 0,04 | 0,1 | -0,03 | 0,01 | -0,03 | 0 |
| **unc_0638** | 0,02 | 0,09 | 0,04 | 0,11 | 0,1 | 0,01 | 0,08 | 0,07 | 0,07 |
| **unc1215** | 0,02 | 0,09 | 0,03 | 0,06 | -0,02 | -0,1 | -0,01 | -0,01 | 0,09 |
| **gsk-lsd1** | 0,02 | 0,06 | 0 | -0,03 | 0,08 | 0,05 | -0,03 | -0,05 | -0,01 |
| **sulcotrione** | 0,02 | 0,05 | 0 | 0,01 | 0 | 0,01 | -0,01 | 0,02 | 0 |
| **doramapimod** | 0,02 | 0,03 | -0,09 | -0,1 | 0,02 | 0,02 | -0,06 | 0,02 | -0,01 |
| **gw7647** | 0,02 | 0,02 | 0,01 | -0,1 | -0,01 | -0,06 | -0,01 | -0,04 | -0,11 |
| **czc-25146** | 0,02 | 0,02 | 0,14 | 0 | -0,07 | -0,03 | 0 | -0,01 | -0,03 |
| **vpc23019** | 0,02 | 0,01 | -0,17 | 0,07 | -0,09 | 0 | -0,03 | -0,13 | -0,22 |
| **cay10650** | 0,02 | 0 | 0,09 | 0,02 | 0,02 | -0,07 | -0,01 | -0,03 | 0 |
| **ibutamoren** | 0,02 | 0 | -0,15 | 0,05 | 0,09 | 0 | -0,02 | 0,06 | 0,03 |
| **stk832240** | 0,02 | -0,01 | -0,01 | 0,01 | -0,12 | -0,09 | 0,05 | -0,01 | -0,1 |
| **rupatadine** | 0,02 | -0,01 | 0,14 | -0,03 | 0,01 | -0,1 | 0 | -0,05 | -0,05 |
| **d399-0002** | 0,02 | -0,02 | 0,05 | 0 | -0,07 | 0,02 | 0 | -0,02 | 0,06 |
| **rivastigmine** | 0,02 | -0,02 | 0,12 | -0,04 | 0,02 | -0,04 | 0,01 | -0,05 | -0,04 |
| **carbamazepine** | 0,02 | -0,07 | -0,01 | 0,01 | 0 | -0,04 | 0,01 | 0,02 | -0,02 |
| **pramipexole** | 0,02 | -0,08 | 0,03 | -0,04 | -0,02 | -0,07 | -0,06 | 0,03 | -0,01 |
| **mifepristone** | 0,02 | -0,09 | 0 | 0 | -0,02 | 0 | -0,06 | -0,11 | -0,04 |
| **sb-224289** | 0,02 | -0,1 | 0,06 | -0,03 | 0,05 | 0 | 0,09 | -0,07 | 0,1 |
| **pap-1** | 0,02 | -0,15 | 0,01 | -0,09 | -0,12 | -0,06 | -0,02 | -0,09 | 0,09 |
| **tirofiban** | 0,02 | -0,27 | 0,04 | 0,01 | 0,01 | 0,05 | 0,03 | 0,13 | 0,01 |
| **ssr_69071** | 0,02 | -0,36 | 0,04 | 0,06 | 0,1 | 0,02 | 0,08 | 0,17 | 0,13 |
| **tafluprost** | 0,02 |  | 0,11 | -0,03 | -0,07 | 0,02 | -0,02 | -0,03 | -0,06 |
| **tcs_1102** | 0,01 | 0,28 | -0,12 | 0,08 | -0,02 | 0,05 | -0,02 | 0,07 | 0,02 |
| **tranexamic_acid** | 0,01 | 0,07 | 0,09 | 0 | -0,05 | 0,04 | 0,04 | -0,06 | -0,06 |
| **vernakalant** | 0,01 | 0,05 | -0,11 | 0,04 | -0,02 | 0 | -0,07 | 0,01 | -0,02 |
| **rolapitant** | 0,01 | 0,05 | -0,02 | -0,02 | -0,03 | -0,02 | 0 | 0,03 | 0,03 |
| **tideglusib** | 0,01 | 0,04 | -0,08 | 0 | 0,01 | -0,03 | 0,03 | -0,03 | 0 |
| **4mu8c** | 0,01 | 0,02 | -0,02 | -0,05 | -0,04 | -0,02 | -0,04 | -0,02 | -0,06 |
| **necrostatin_2** | 0,01 | -0,02 | 0,01 | -0,06 | -0,03 | -0,02 | -0,09 | 0,02 | 0 |
| **ly_487379** | 0,01 | -0,02 | -0,01 | -0,05 | -0,07 | -0,15 | -0,13 | 0,08 | -0,05 |
| **ssr128129e** | 0,01 | -0,02 | -0,05 | -0,02 | 0,02 | 0,01 | -0,01 | -0,02 | -0,02 |
| **macitentan** | 0,01 | -0,02 | -0,01 | -0,04 | -0,01 | -0,04 | -0,02 | -0,02 | -0,03 |
| **linagliptin** | 0,01 | -0,03 | 0,1 | 0 | 0,02 | -0,01 | -0,07 | -0,02 | -0,02 |
| **fevipiprant** | 0,01 | -0,04 | -0,05 | -0,13 | -0,03 | -0,02 | -0,04 | -0,02 | -0,02 |
| **darunavir** | 0,01 | -0,06 | -0,01 | -0,03 | 0,04 | 0,04 | -0,02 | -0,04 | 0 |
| **i3c** | 0,01 | -0,1 | 0,06 | 0,08 | -0,02 | 0,12 | 0,04 | 0 | -0,01 |
| **gw9508** | 0,01 | -0,16 | 0,05 | 0 | 0,02 | 0,11 | 0 | 0,06 | 0 |
| **hjc0350** | 0,01 | -0,17 | 0,01 | -0,04 | 0 | 0,03 | -0,04 | 0,02 | -0,04 |
| **stk869906** | 0,01 | -0,19 | 0,21 | 0,06 | -0,06 | -0,01 | 0,01 | -0,04 | -0,04 |
| **chembl1348080** | 0,01 | -0,19 | -0,04 | -0,12 | -0,04 | 0,02 | -0,02 | -0,01 | 0,04 |
| **stf-31** | 0,01 | -0,19 | -0,02 | 0,06 | 0,14 | 0,05 | -0,01 | 0,03 | 0 |
| **bafilomycin_a1** | 0,01 | -0,46 | 0,01 | -0,04 | -0,05 | 0,15 | 0,13 | -0,08 | 0,06 |
| **(s)-zopiclone** | 0,01 |  | 0,03 | 0,03 | 0,03 | 0,03 | -0,05 | 0,07 | 0 |
| **ruboxistaurin** | 0,01 |  | 0,17 | 0,09 | 0,07 | 0,09 | 0,17 | 0,04 | 0,05 |
| **sh-4-54** | 0,01 |  | 0,02 | 0 | 0,05 | -0,02 | 0,07 | 0,02 | 0 |
| **pyrimethamine** | 0,01 |  | -0,01 | -0,02 | 0,03 | 0,01 | 0,06 | -0,04 | 0,06 |
| **n6022** | 0 | 0,29 | -0,01 | -0,02 | -0,07 | 0,04 | 0,05 | -0,03 | -0,02 |
| **ml167** | 0 | 0,26 | 0,11 | 0,01 | -0,07 | 0,03 | 0 | -0,09 | 0,01 |
| **np118809** | 0 | 0,12 | -0,06 | -0,04 | -0,05 | 0,02 | -0,08 | -0,09 | -0,05 |
| **atosiban** | 0 | 0,11 | 0,01 | -0,02 | -0,08 | -0,03 | 0,01 | -0,1 | -0,05 |
| **mls0315771** | 0 | 0,1 | -0,05 | 0,17 | -0,03 | 0,02 | -0,12 | 0,01 | -0,04 |
| **quetiapine** | 0 | 0,09 | -0,09 | 0,03 | 0,01 | -0,01 | 0,01 | 0,05 | 0 |
| **(z)-pugnac** | 0 | 0,08 | 0,02 | 0,01 | 0 | -0,07 | -0,01 | 0,06 | 0,01 |
| **3-benzyloxyaniline** | 0 | 0,05 | 0,01 | 0,04 | 0,01 | -0,05 | -0,03 | 0,02 | 0,01 |
| **pazopanib** | 0 | 0,05 | -0,02 | 0,01 | 0,02 | 0 | 0 | -0,04 | -0,04 |
| **qx_314** | 0 | 0,03 | 0,03 | -0,01 | 0,04 | 0,02 | -0,03 | -0,1 | -0,03 |
| **ro_28-1675** | 0 | 0,03 | -0,02 | 0 | 0,02 | 0,01 | 0,04 | -0,01 | -0,01 |
| **cyppa** | 0 | 0,02 | 0,02 | 0,1 | -0,02 | -0,02 | 0,04 | 0,06 | -0,05 |
| **zinc2093649** | 0 | 0,02 | -0,05 | 0,01 | -0,06 | -0,02 | 0 | -0,02 | 0,07 |
| **l-165041** | 0 | 0,02 | -0,16 | -0,05 | -0,02 | -0,01 | 0,12 | 0,03 | 0 |
| **eltrombopag** | 0 | 0,01 | -0,14 | -0,06 | 0,09 | -0,06 | -0,02 | -0,01 | -0,03 |
| **atglistatin** | 0 | 0 | -0,02 | 0,05 | -0,03 | -0,04 | 0,03 | 0,14 | 0,07 |
| **chembl1436068** | 0 | 0 | -0,01 | -0,11 | 0,02 | -0,01 | -0,03 | -0,02 | 0,04 |
| **diethylstilbestrol** | 0 | 0 | -0,04 | -0,02 | -0,03 | 0,02 | 0,01 | 0,03 | -0,03 |
| **indomethacin** | 0 | 0 | -0,05 | -0,04 | 0,04 | 0,03 | -0,02 | -0,06 | -0,04 |
| **a_438079** | 0 | 0 | 0,05 | 0,03 | 0,01 | 0,05 | 0,03 | 0,05 | 0,04 |
| **eletriptan** | 0 | -0,01 | -0,02 | -0,13 | 0,06 | -0,04 | -0,03 | -0,03 | -0,04 |
| **3_5-dichlorosalicylic_acid** | 0 | -0,02 | 0,06 | 0,01 | 0,02 | -0,17 | 0,13 | -0,04 | 0,05 |
| **mirabegron** | 0 | -0,02 | -0,03 | -0,07 | 0 | -0,03 | 0 | -0,05 | -0,01 |
| **bd_1008** | 0 | -0,03 | -0,07 | -0,01 | -0,09 | -0,18 | -0,08 | -0,03 | -0,07 |
| **pci-34051** | 0 | -0,04 | 0,01 | 0 | 0,08 | 0 | 0,08 | 0 | 0,04 |
| **chlorsulfuron** | 0 | -0,05 | -0,05 | -0,07 | -0,02 | 0,01 | -0,02 | -0,02 | -0,05 |
| **nbtgr** | 0 | -0,05 | -0,01 | -0,02 | -0,03 | -0,05 | -0,03 | 0 | -0,05 |
| **gw311616** | 0 | -0,06 | -0,01 | -0,02 | -0,01 | -0,01 | -0,04 | 0,01 | -0,04 |
| **dp-agonist13** | 0 | -0,07 | -0,02 | 0 | -0,03 | 0,03 | 0,07 | 0,07 | 0,06 |
| **mk2206** | 0 | -0,07 | -0,06 | 0,07 | -0,03 | 0,02 | -0,05 | 0,13 | 0,01 |
| **cytosporone_b** | 0 | -0,08 | -0,02 | 0 | -0,06 | -0,01 | 0 | -0,06 | -0,02 |
| **pinometostat** | 0 | -0,1 | -0,02 | 0 | 0,02 | 0,01 | 0,04 | 0,01 | 0 |
| **octreotide** | 0 | -0,1 | 0 | 0 | 0 | 0 | 0 | -0,06 | -0,01 |
| **ak_7** | 0 | -0,11 | 0,01 | 0,01 | -0,01 | 0 | 0,02 | 0,02 | -0,04 |
| **ly_344864** | 0 | -0,14 | 0,11 | -0,1 | -0,16 | -0,11 | -0,08 | -0,06 | -0,09 |
| **d-(+)-glucose** | 0 | -0,14 | -0,01 | 0,01 | -0,03 | -0,08 | -0,01 | 0 | -0,02 |
| **selisistat** | 0 |  | -0,02 | -0,04 | 0 | -0,03 | 0,04 | 0,05 | -0,02 |
| **y-33075** | 0 |  | 0,11 | 0,09 | 0,04 | 0,11 | 0,09 | 0,11 | 0,12 |
| **volasertib** | -0,01 | 0,47 | -0,07 | -0,07 | -0,02 | 0 | -0,01 | -0,07 | 0,02 |
| **esmolol** | -0,01 | 0,28 | 0,02 | 0,01 | 0 | -0,06 | 0,01 | 0,05 | -0,04 |
| **nnc_26-9100** | -0,01 | 0,23 | -0,08 | 0,1 | -0,19 | -0,05 | -0,06 | 0,12 | -0,04 |
| **bw_723c86** | -0,01 | 0,04 | -0,05 | -0,03 | 0,01 | -0,03 | 0,02 | 0,06 | -0,04 |
| **progesterone** | -0,01 | 0,04 | 0,02 | 0,01 | -0,03 | -0,02 | 0,02 | -0,01 | 0,01 |
| **isotretinoin** | -0,01 | 0,02 | -0,04 | 0,01 | -0,01 | 0,03 | -0,02 | 0,05 | 0,11 |
| **jzl_184** | -0,01 | 0,01 | -0,03 | -0,02 | -0,01 | -0,02 | -0,01 | 0 | -0,04 |
| **nsc_23766** | -0,01 | -0,01 | -0,01 | -0,05 | -0,02 | 0,03 | -0,01 | -0,06 | 0,01 |
| **eptifibatide** | -0,01 | -0,03 | -0,04 | -0,14 | -0,12 | -0,06 | -0,05 | 0,01 | -0,03 |
| **buspirone** | -0,01 | -0,04 | -0,07 | -0,03 | 0,01 | -0,15 | -0,16 | 0,19 | -0,07 |
| **tcs_pim-1_1** | -0,01 | -0,07 | -0,01 | 0,01 | -0,02 | -0,01 | -0,04 | 0,02 | -0,03 |
| **rwj_50271** | -0,01 | -0,08 | 0 | -0,03 | -0,12 | 0,03 | -0,03 | -0,06 | -0,02 |
| **bisphenol_a** | -0,01 | -0,1 | 0,02 | -0,04 | 0,12 | 0,09 | 0,01 | 0,06 | -0,05 |
| **varespladib** | -0,01 | -0,11 | 0,08 | -0,05 | -0,07 | -0,09 | -0,04 | 0 | -0,05 |
| **mianserin** | -0,01 | -0,13 | 0,01 | -0,02 | -0,06 | -0,06 | -0,05 | -0,01 | -0,01 |
| **t3** | -0,01 | -0,17 | 0 | -0,05 | -0,1 | -0,03 | -0,03 | -0,06 | 0,02 |
| **a_922500** | -0,01 | -0,2 | 0,04 | -0,05 | 0,02 | -0,04 | -0,03 | 0 | 0 |
| **5-ethynyluracil** | -0,01 | -0,21 | 0,04 | -0,02 | -0,02 | -0,04 | -0,05 | 0 | 0,01 |
| **rifaximin** | -0,01 | -0,37 | -0,08 | 0 | -0,05 | 0 | 0,03 | 0,12 | 0,06 |
| **[pyr1]-apelin-13** | -0,01 |  | 0,02 | -0,08 | -0,05 | 0,03 | -0,02 | 0,05 | 0,01 |
| **(rs)-ppg** | -0,02 | 0,37 | 0,05 | -0,07 | 0 | 0,01 | 0,03 | 0,03 | 0 |
| **sb_525334** | -0,02 | 0,18 | 0,07 | 0,03 | 0,04 | 0,04 | -0,01 | -0,2 | 0,01 |
| **amg_487** | -0,02 | 0,14 | -0,1 | -0,07 | -0,04 | -0,03 | 0 | 0,02 | 0,03 |
| **5-r-rivaroxaban** | -0,02 | 0,11 | -0,08 | -0,01 | -0,02 | 0 | -0,04 | 0,03 | -0,07 |
| **tak-779** | -0,02 | 0,07 | -0,02 | -0,02 | 0,03 | 0,05 | -0,01 | -0,06 | -0,05 |
| **sitagliptin** | -0,02 | 0,06 | 0,16 | -0,03 | -0,02 | -0,07 | -0,02 | 0,03 | 0,02 |
| **xen_103** | -0,02 | 0,04 | 0,1 | 0 | -0,07 | -0,01 | 0,07 | -0,03 | -0,01 |
| **tram-34** | -0,02 | 0,04 | -0,04 | -0,04 | 0,01 | -0,03 | 0 | -0,01 | 0 |
| **bmy_7378** | -0,02 | 0,02 | -0,01 | -0,08 | 0 | 0,11 | 0,03 | 0,1 | -0,02 |
| **sb290157** | -0,02 | 0,02 | -0,08 | -0,05 | 0 | -0,19 | -0,08 | -0,01 | -0,01 |
| **mk-6892** | -0,02 | 0,02 | -0,03 | 0 | 0,06 | 0,01 | -0,07 | -0,07 | 0,09 |
| **capsazepine** | -0,02 | 0 | 0,09 | 0,04 | 0,02 | 0 | 0,04 | -0,04 | 0,04 |
| **l-168_049** | -0,02 | -0,01 | 0,02 | 0,06 | -0,05 | -0,02 | -0,05 | 0,02 | -0,01 |
| **dichlorisone** | -0,02 | -0,01 | -0,03 | 0,05 | -0,04 | 0,02 | -0,05 | -0,2 | 0,05 |
| **dronedarone** | -0,02 | -0,02 | -0,01 | -0,17 | 0,05 | -0,02 | 0,01 | -0,03 | 0 |
| **molindone** | -0,02 | -0,05 | -0,02 | -0,06 | -0,05 | -0,06 | -0,09 | -0,04 | -0,12 |
| **gw_501516** | -0,02 | -0,06 | 0,07 | -0,03 | -0,02 | -0,03 | 0,01 | -0,04 | -0,05 |
| **c-fms-in-3** | -0,02 | -0,06 | -0,07 | -0,07 | -0,02 | -0,05 | -0,06 | -0,06 | -0,05 |
| **ro495** | -0,02 | -0,09 | 0,15 | 0,19 | 0,01 | 0,2 | 0,1 | 0,01 | 0,24 |
| **pimavanserin** | -0,02 | -0,09 | -0,02 | -0,05 | 0,02 | 0,12 | 0,14 | 0,09 | 0,13 |
| **bht** | -0,02 | -0,09 | -0,19 | 0,03 | -0,04 | -0,03 | -0,02 | 0,1 | 0,04 |
| **bace1** | -0,02 | -0,1 | -0,05 | -0,01 | -0,08 | -0,08 | -0,06 | -0,05 | -0,05 |
| **zinc8945** | -0,02 | -0,11 | -0,07 | 0,01 | -0,04 | 0,06 | 0,04 | -0,07 | -0,01 |
| **azd1152** | -0,02 | -0,11 | 0 | 0,04 | 0,07 | 0 | 0,13 | -0,07 | 0,07 |
| **aztreonam** | -0,02 | -0,11 | -0,01 | -0,07 | 0 | -0,01 | 0 | -0,02 | -0,02 |
| **bromopyruvic_acid** | -0,02 | -0,13 | 0 | 0,04 | 0,05 | -0,03 | -0,04 | -0,07 | 0 |
| **ethynyl_estradiol** | -0,02 | -0,21 | 0,16 | 0,03 | -0,04 | -0,03 | -0,02 | 0,02 | 0,03 |
| **ethiprole** | -0,02 | -0,22 | -0,06 | -0,02 | -0,01 | -0,02 | -0,03 | -0,03 | -0,02 |
| **rucinol** | -0,02 | -0,22 | -0,03 | 0 | 0,02 | 0,03 | 0,08 | -0,09 | 0,04 |
| **icatibant** | -0,02 |  | -0,02 | -0,04 | 0,01 | -0,08 | 0 | -0,02 | -0,02 |
| **tizanidine** | -0,03 | 0,37 | 0,03 | 0,02 | -0,02 | -0,01 | -0,07 | 0 | 0,01 |
| **mpi-0479605-hcl** | -0,03 | 0,24 | -0,08 | -0,02 | -0,04 | -0,12 | -0,02 | 0,12 | -0,11 |
| **balicatib** | -0,03 | 0,16 | -0,01 | -0,05 | -0,03 | -0,07 | 0,01 | -0,12 | 0 |
| **ku-60019** | -0,03 | 0,16 | 0,02 | 0,1 | -0,02 | 0,11 | 0,03 | -0,02 | 0,1 |
| **nafamostat** | -0,03 | 0,12 | -0,01 | -0,01 | 0 | 0,02 | 0 | -0,01 | 0,04 |
| **3-benzotriazol-propionic_acid** | -0,03 | 0,09 | 0,03 | -0,07 | -0,04 | -0,12 | -0,01 | 0 | -0,07 |
| **stk772817** | -0,03 | 0,07 | -0,02 | -0,04 | -0,13 | -0,07 | 0,02 | 0,01 | -0,03 |
| **gsk-923295** | -0,03 | 0,06 | 0,06 | 0,13 | 0,19 | 0,16 | 0,02 | 0,07 | 0,14 |
| **ripasudil** | -0,03 | 0,05 | 0,02 | -0,01 | 0 | 0,02 | 0,01 | 0 | 0,02 |
| **tmp269** | -0,03 | 0,01 | -0,02 | 0,07 | 0,12 | 0,02 | 0,07 | 0,03 | 0,03 |
| **siponimod** | -0,03 | 0,01 | 0,13 | 0,03 | -0,09 | -0,04 | 0,03 | -0,02 | -0,07 |
| **stk368746** | -0,03 | -0,03 | -0,01 | -0,03 | 0,1 | 0,02 | -0,01 | -0,06 | 0,08 |
| **fluorescein** | -0,03 | -0,04 | -0,04 | 0,02 | -0,03 | -0,11 | -0,03 | 0,21 | 0,08 |
| **zinc6198410** | -0,03 | -0,04 | 0,03 | 0,08 | -0,03 | -0,06 | -0,01 | 0,03 | -0,09 |
| **2_4-dichlorophenol** | -0,03 | -0,05 | -0,02 | 0 | 0,01 | -0,01 | -0,01 | 0 | -0,02 |
| **chca** | -0,03 | -0,05 | -0,07 | -0,2 | 0,02 | 0,03 | -0,07 | 0,07 | -0,13 |
| **zj_43** | -0,03 | -0,05 | 0,05 | 0 | -0,07 | -0,01 | -0,02 | -0,03 | -0,03 |
| **s107** | -0,03 |  | 0,06 | -0,06 | 0,01 | 0 | 0,01 | 0,01 | -0,01 |
| **zofenopril** | -0,03 |  | -0,04 | 0 | -0,07 | -0,01 | -0,02 | -0,05 | -0,02 |
| **cpi-203** | -0,04 | 0,39 | -0,15 | -0,1 | -0,04 | 0,07 | -0,17 | -0,14 | -0,09 |
| **gsk126** | -0,04 | 0,11 | -0,12 | -0,04 | -0,04 | -0,05 | -0,01 | 0,06 | 0,01 |
| **abiraterone1** | -0,04 | 0,09 | 0,08 | 0 | -0,07 | 0,03 | -0,05 | -0,07 | -0,12 |
| **h_89** | -0,04 | 0,08 | -0,01 | 0,01 | 0,04 | 0,05 | 0,01 | 0,09 | 0,06 |
| **acetazolamide** | -0,04 | 0,05 | -0,14 | -0,07 | -0,02 | 0,03 | 0 | 0,16 | 0,04 |
| **stk410281** | -0,04 | 0,03 | 0,04 | 0 | -0,03 | -0,04 | -0,04 | -0,02 | -0,08 |
| **ly411575** | -0,04 | 0,02 | 0,37 | -0,03 | -0,27 | -0,09 | 0,09 | -0,17 | -0,14 |
| **acenaphthoquinone** | -0,04 | 0,02 | 0 | -0,01 | -0,01 | 0,03 | -0,01 | 0,06 | 0 |
| **chembl1597046** | -0,04 | -0,01 | -0,07 | -0,02 | 0,07 | -0,05 | 0 | -0,05 | -0,06 |
| **cj-42794** | -0,04 | -0,05 | 0 | 0,03 | -0,09 | -0,07 | -0,05 | -0,05 | -0,01 |
| **sb-222200** | -0,04 | -0,1 | -0,02 | 0 | -0,01 | 0 | -0,01 | 0,08 | 0,03 |
| **bec** | -0,04 | -0,12 | -0,04 | -0,01 | -0,05 | -0,01 | -0,05 | 0,05 | 0 |
| **cyclopamine** | -0,04 | -0,13 | 0,04 | 0,01 | 0,01 | 0,02 | -0,01 | 0,02 | -0,01 |
| **pirenzepine.1** | -0,04 | -0,2 | 0,02 | -0,08 | 0,04 | 0,07 | 0,08 | -0,02 | 0,02 |
| **rn-1734** | -0,04 | -0,23 | -0,03 | -0,03 | 0,01 | -0,04 | 0 | 0 | -0,02 |
| **axl1717** | -0,04 | -0,26 | 0,05 | 0,06 | 0,14 | 0,17 | 0,01 | -0,09 | 0,1 |
| **fmlf** | -0,04 |  | 0 | -0,04 | -0,04 | 0,01 | 0,01 | 0,01 | 0,01 |
| **ulipristal** | -0,05 | 0,2 | -0,02 | -0,03 | 0,01 | -0,07 | -0,06 | -0,1 | -0,06 |
| **carprofen** | -0,05 | 0,15 | 0 | 0,02 | 0,01 | -0,03 | 0 | 0 | -0,02 |
| **ruxolitinib** | -0,05 | 0,13 | 0,01 | 0,09 | 0 | 0,03 | 0,05 | -0,05 | 0,04 |
| **clozapine** | -0,05 | 0,12 | -0,01 | 0,04 | 0 | -0,06 | -0,01 | 0,02 | 0 |
| **aliskiren** | -0,05 | 0,11 | -0,05 | -0,06 | -0,01 | 0,01 | 0 | 0,07 | -0,02 |
| **pd_123319** | -0,05 | 0,1 | 0,03 | 0,01 | -0,02 | 0 | -0,01 | 0 | -0,03 |
| **ly2365109** | -0,05 | 0,05 | 0 | -0,07 | -0,06 | -0,05 | -0,01 | 0,06 | -0,01 |
| **paliperidone** | -0,05 | -0,02 | -0,1 | -0,16 | -0,04 | -0,06 | -0,05 | 0 | 0,03 |
| **px-12** | -0,05 | -0,06 | 0,04 | 0,01 | 0,05 | 0,14 | 0 | 0 | 0,11 |
| **ibutilide** | -0,05 | -0,11 | -0,16 | 0,04 | 0 | -0,04 | -0,02 | 0,04 | 0,08 |
| **erythromycin** | -0,05 | -0,19 | 0,06 | -0,02 | -0,01 | -0,08 | 0,02 | 0,01 | 0,01 |
| **gne-9605** | -0,05 | -0,27 | 0,06 | 0 | 0,03 | 0,04 | -0,01 | -0,1 | 0 |
| **papaverine** | -0,05 |  | -0,08 | -0,11 | -0,06 | -0,03 | -0,05 | -0,04 | -0,07 |
| **gsk2656157** | -0,05 |  | -0,05 | 0 | 0,03 | 0 | 0,01 | 0,03 | -0,01 |
| **rs_67333** | -0,06 | 0,14 | -0,02 | -0,1 | 0 | 0,01 | 0,11 | 0,05 | 0,04 |
| **gsk343** | -0,06 | 0,09 | -0,05 | -0,01 | -0,06 | 0,07 | -0,07 | 0,02 | -0,06 |
| **azd1152-hqpa** | -0,06 | 0,08 | 0,04 | 0,05 | 0,02 | 0,03 | 0,07 | 0 | 0,02 |
| **eplerenone** | -0,06 | 0,08 | 0,03 | -0,04 | -0,07 | 0 | 0,05 | 0,04 | 0 |
| **acifran** | -0,06 | 0,07 | 0,01 | 0,04 | 0 | 0 | 0,09 | 0,01 | 0,02 |
| **pde-9i** | -0,06 | 0,07 | -0,06 | -0,04 | -0,01 | -0,13 | 0,02 | -0,03 | -0,06 |
| **lcl161** | -0,06 | 0,03 | 0 | 0,02 | -0,14 | 0 | -0,03 | -0,06 | -0,1 |
| **montelukast** | -0,06 | 0 | 0,03 | -0,05 | 0 | -0,03 | -0,05 | -0,02 | -0,06 |
| **mi-3** | -0,06 | -0,01 | -0,09 | 0,28 | 0 | -0,03 | -0,04 | -0,02 | -0,05 |
| **navarixin** | -0,06 | -0,01 | 0,23 | -0,02 | -0,03 | -0,06 | -0,02 | -0,01 | 0 |
| **probenecid** | -0,06 | -0,04 | 0,1 | -0,08 | -0,04 | -0,03 | -0,01 | 0 | 0 |
| **ac-yvad-cho** | -0,06 | -0,05 | -0,02 | -0,01 | -0,02 | -0,05 | -0,04 | 0,05 | -0,03 |
| **laropiprant** | -0,06 | -0,07 | 0,01 | 0,05 | 0 | -0,04 | -0,01 | -0,02 | -0,03 |
| **desvenlafaxine** | -0,06 | -0,11 | 0,02 | 0,08 | -0,01 | 0,03 | 0,01 | -0,06 | -0,02 |
| **incb_3284** | -0,06 | -0,12 | -0,01 | -0,03 | -0,12 | -0,07 | -0,01 | 0,01 | -0,02 |
| **cay10683** | -0,06 | -0,15 | -0,03 | -0,03 | -0,04 | -0,03 | -0,03 | 0,01 | -0,04 |
| **fluticasone** | -0,06 | -0,22 | 0,09 | -0,03 | -0,03 | 0,01 | -0,01 | 0,07 | 0,01 |
| **darapladib** | -0,06 |  | -0,01 | -0,05 | -0,04 | -0,01 | -0,04 | 0 | -0,05 |
| **tpca-1** | -0,07 | 0,45 | 0,1 | 0,15 | -0,04 | 0,09 | -0,04 | -0,04 | -0,07 |
| **zaltoprofen** | -0,07 | 0,29 | -0,06 | -0,05 | 0 | -0,15 | -0,12 | -0,1 | -0,11 |
| **rh00511** | -0,07 | 0,12 | 0 | -0,03 | 0,01 | 0 | -0,01 | -0,04 | 0,03 |
| **bosentan** | -0,07 | 0,07 | 0,17 | 0,05 | -0,07 | -0,1 | -0,01 | 0 | 0,02 |
| **bms-5** | -0,07 | 0,06 | 0 | -0,02 | -0,03 | -0,01 | 0 | 0,01 | -0,01 |
| **5-hydroxyflavone** | -0,07 | 0,05 | 0,24 | -0,02 | 0 | -0,04 | 0,03 | -0,2 | -0,07 |
| **bbl023172** | -0,07 | 0,04 | -0,08 | 0,03 | 0,01 | -0,05 | 0,05 | -0,03 | -0,02 |
| **lapatinib** | -0,07 | 0,04 | 0,06 | 0,11 | 0,06 | 0,15 | 0,05 | 0,04 | 0,06 |
| **sb-269970** | -0,07 | 0,04 | 0,01 | -0,02 | 0,01 | -0,03 | -0,04 | -0,04 | -0,04 |
| **chembl2431803** | -0,07 | 0,04 | -0,01 | -0,05 | -0,02 | 0,02 | -0,08 | 0,02 | 0,02 |
| **methazolamide** | -0,07 | 0,03 | -0,04 | -0,04 | -0,03 | -0,02 | -0,05 | 0,06 | 0,03 |
| **l-cysteine** | -0,07 | 0,02 | 0,07 | -0,12 | 0 | 0,02 | 0,04 | -0,08 | 0,06 |
| **roxatidine** | -0,07 | -0,01 | 0,02 | 0 | 0,05 | -0,04 | -0,01 | -0,06 | 0 |
| **fumitremorgin_c** | -0,07 | -0,02 | -0,05 | -0,03 | 0,07 | -0,14 | -0,03 | -0,01 | 0,03 |
| **vilazodone** | -0,07 | -0,03 | 0,11 | -0,03 | 0,03 | 0,02 | 0,31 | -0,06 | -0,12 |
| **kh7** | -0,07 | -0,04 | 0,03 | 0,06 | 0,08 | 0,17 | 0,03 | -0,06 | 0,11 |
| **oleanolic_acid** | -0,07 | -0,04 | 0,01 | 0,01 | 0,03 | -0,06 | -0,02 | 0 | -0,04 |
| **tezacaftor** | -0,07 | -0,04 | 0 | 0 | -0,01 | -0,09 | -0,01 | -0,01 | -0,02 |
| **gsk2606414** | -0,07 | -0,05 | -0,17 | -0,03 | 0,03 | 0 | -0,05 | -0,04 | 0,04 |
| **yohimbine** | -0,07 | -0,07 | 0,07 | 0,02 | 0,01 | -0,06 | 0,02 | 0,01 | 0 |
| **rosiglitazone** | -0,07 | -0,07 | 0,07 | -0,03 | 0 | -0,08 | 0,01 | -0,02 | -0,02 |
| **a_1120** | -0,07 | -0,08 | 0,08 | 0 | -0,04 | -0,07 | -0,01 | 0 | -0,03 |
| **terbinafine** | -0,07 | -0,09 | 0,07 | 0,02 | -0,01 | -0,02 | 0,01 | 0 | -0,01 |
| **ha130** | -0,07 | -0,09 | 0,02 | 0,03 | 0,03 | -0,06 | -0,03 | 0,05 | 0,01 |
| **stk410283** | -0,07 | -0,1 | -0,01 | -0,09 | -0,02 | -0,08 | -0,04 | -0,03 | -0,03 |
| **olvance** | -0,07 | -0,12 | -0,04 | -0,04 | 0,04 | -0,09 | -0,02 | 0,01 | -0,01 |
| **kh-cb19** | -0,07 | -0,19 | -0,08 | 0,04 | -0,02 | 0 | -0,04 | -0,03 | -0,07 |
| **pitolisant** | -0,07 | -0,47 | 0,19 | 0 | 0,03 | -0,03 | 0,04 | 0,07 | -0,03 |
| **uridine** | -0,07 |  | 0,03 | -0,05 | -0,04 | 0,03 | -0,05 | 0,01 | 0,02 |
| **gw_441756** | -0,07 |  | 0,01 | -0,07 | -0,01 | -0,07 | -0,08 | 0,09 | -0,03 |
| **lomitapide** | -0,07 |  | -0,02 | -0,01 | -0,08 | -0,01 | -0,05 | -0,05 | -0,07 |
| **castanospermine** | -0,08 | 0,08 | -0,06 | -0,03 | -0,02 | -0,02 | -0,1 | -0,02 | -0,05 |
| **epristeride** | -0,08 | 0,02 | 0 | -0,01 | -0,04 | -0,08 | -0,02 | 0,04 | 0 |
| **desipramine** | -0,08 | 0,01 | -0,04 | 0,01 | 0,01 | -0,1 | -0,1 | 0,02 | -0,03 |
| **bergenin** | -0,08 | 0 | -0,03 | -0,09 | 0,02 | 0,01 | -0,06 | -0,05 | -0,09 |
| **mk-3207** | -0,08 | -0,02 | -0,04 | -0,03 | -0,01 | -0,02 | -0,03 | 0,01 | -0,02 |
| **rucaparib** | -0,08 | -0,02 | -0,09 | 0,01 | 0,01 | -0,03 | -0,05 | 0,15 | -0,09 |
| **aa92593** | -0,08 | -0,03 | -0,1 | 0,15 | -0,08 | 0,29 | 0,05 | 0,35 | -0,07 |
| **ac-7954** | -0,08 | -0,03 | -0,02 | 0,06 | -0,05 | -0,1 | -0,05 | -0,11 | -0,05 |
| **schembl5061501** | -0,08 | -0,06 | 0,05 | -0,08 | 0,03 | -0,04 | 0,01 | -0,01 | -0,08 |
| **guanfacine** | -0,08 | -0,07 | -0,06 | 0 | 0 | 0,01 | 0,01 | -0,01 | 0 |
| **tipiracil** | -0,08 | -0,11 | 0,02 | -0,03 | -0,08 | -0,05 | 0 | -0,03 | -0,04 |
| **ewp-815** | -0,08 | -0,14 | 0,01 | 0,03 | -0,07 | 0,05 | 0 | -0,09 | -0,01 |
| **retigabine** | -0,08 | -0,18 | -0,04 | 0 | -0,04 | -0,02 | -0,04 | 0 | -0,02 |
| **benzbromarone** | -0,08 | -0,19 | 0,01 | 0 | -0,01 | -0,12 | 0,01 | 0,01 | 0 |
| **d-3263** | -0,08 |  | -0,01 | -0,09 | -0,07 | 0,07 | -0,06 | -0,06 | -0,1 |
| **unc_0631** | -0,08 |  | -0,04 | -0,13 | -0,02 | 0,02 | -0,01 | -0,13 | -0,11 |
| **ak-54059** | -0,09 | 0,06 | -0,07 | -0,07 | 0,03 | -0,05 | -0,05 | 0,13 | 0 |
| **nsc95682** | -0,09 | 0,06 | 0,01 | -0,04 | 0,02 | 0,17 | 0,01 | 0,06 | 0,03 |
| **nu_7026** | -0,09 | 0,01 | 0,01 | 0,01 | 0,02 | 0,04 | 0 | -0,01 | -0,01 |
| **2-cl-ib-meca** | -0,09 | 0,01 | 0,03 | 0,06 | -0,02 | -0,01 | -0,05 | 0,01 | 0 |
| **telmisartan** | -0,09 | 0 | -0,06 | -0,03 | -0,08 | -0,01 | -0,02 | 0,01 | -0,06 |
| **nbi-74330** | -0,09 | -0,03 | -0,03 | -0,06 | -0,01 | -0,09 | -0,01 | -0,07 | -0,04 |
| **dihydroartemisinin** | -0,09 | -0,13 | 0,04 | 0,1 | -0,01 | 0,07 | 0,09 | -0,01 | 0,05 |
| **zd_7288** | -0,09 | -0,14 | -0,01 | -0,08 | 0 | 0,02 | 0,02 | 0,08 | -0,02 |
| **repaglinide** | -0,09 | -0,17 | 0,05 | 0 | -0,07 | -0,04 | -0,01 | -0,06 | -0,04 |
| **vemurafenib** | -0,09 | -0,36 | 0,02 | -0,04 | 0,02 | -0,03 | -0,03 | -0,02 | 0,04 |
| **stk746869** | -0,1 | 0,18 | -0,02 | 0,06 | 0,05 | 0,06 | 0,01 | 0,04 | 0,03 |
| **gsk429286a** | -0,1 | 0,16 | -0,12 | 0,02 | 0,03 | -0,04 | -0,04 | -0,05 | 0,03 |
| **chembl217125** | -0,1 | 0,07 | 0,05 | -0,09 | 0,05 | -0,06 | 0,02 | 0,04 | 0,03 |
| **ve-821** | -0,1 | 0,06 | -0,09 | -0,02 | 0,04 | -0,01 | 0 | 0 | 0,05 |
| **mln_8237** | -0,1 | 0 | -0,04 | 0,05 | 0,08 | 0,07 | 0,03 | 0,02 | 0,06 |
| **z32036776** | -0,1 | 0 | 0,07 | -0,03 | 0,06 | 0,03 | 0,02 | 0 | -0,04 |
| **tcs-ox2-29** | -0,1 | 0 | 0 | 0,01 | 0,03 | -0,02 | -0,03 | 0,08 | -0,01 |
| **kaempferide** | -0,1 | -0,02 | 0,01 | -0,05 | 0 | 0,02 | -0,01 | 0,01 | -0,04 |
| **vtp-27999** | -0,1 | -0,02 | -0,01 | 0 | 0 | -0,03 | 0,02 | 0 | -0,04 |
| **cimetidine** | -0,1 | -0,03 | 0,04 | 0,04 | 0,03 | -0,07 | 0,02 | -0,01 | 0,05 |
| **marimastat** | -0,1 | -0,05 | -0,03 | 0,02 | -0,03 | -0,02 | 0 | -0,02 | -0,01 |
| **methylnaltrexone** | -0,1 | -0,05 | 0,05 | 0,03 | -0,04 | -0,02 | 0 | 0,01 | -0,02 |
| **10074-g5** | -0,1 | -0,13 | -0,04 | -0,02 | -0,02 | -0,09 | -0,06 | 0,03 | -0,02 |
| **erlotinib** | -0,1 | -0,18 | 0 | 0,02 | 0,01 | 0,01 | -0,02 | 0,08 | 0,01 |
| **sb-408124** | -0,1 | -0,25 | -0,03 | -0,04 | 0,06 | -0,08 | -0,02 | -0,03 | -0,04 |
| **mavorixafor** | -0,1 |  | -0,02 | -0,05 | -0,11 | -0,06 | -0,04 | 0,04 | -0,03 |
| **sd-208** | -0,11 | 0,24 | 0,06 | -0,14 | 0 | -0,03 | -0,03 | 0,03 | -0,1 |
| **l-741** | -0,11 | 0,07 | -0,02 | -0,04 | 0,04 | 0,03 | 0,02 | 0,07 | 0,03 |
| **p-tolylboronic_acid** | -0,11 | -0,01 | -0,01 | -0,02 | -0,01 | -0,06 | 0,03 | 0,05 | -0,04 |
| **rotigotine** | -0,11 | -0,01 | -0,02 | 0,03 | -0,02 | -0,02 | 0,07 | -0,14 | 0,02 |
| **dapagliflozin** | -0,11 | -0,02 | -0,04 | -0,02 | 0 | -0,05 | 0,01 | -0,13 | -0,03 |
| **vicriviroc** | -0,11 | -0,07 | 0,08 | 0,01 | 0,02 | 0,06 | 0,05 | 0,04 | -0,04 |
| **mf63** | -0,11 | -0,09 | 0,01 | 0,09 | 0,03 | 0,03 | -0,01 | 0,03 | 0,1 |
| **luf-5735** | -0,11 | -0,11 | 0,1 | 0,05 | 0,02 | 0,06 | 0,04 | 0,03 | -0,01 |
| **ch-223191** | -0,11 | -0,14 | 0,06 | -0,07 | 0 | -0,08 | -0,1 | -0,08 | -0,03 |
| **chembl200403** | -0,11 | -0,19 | 0,2 | 0,21 | 0,19 | 0,15 | 0,2 | 0,01 | 0,16 |
| **dapiprazole** | -0,11 | -0,19 | -0,03 | 0,01 | 0,04 | -0,03 | -0,03 | -0,07 | 0 |
| **onx_0914** | -0,11 | -0,94 | 0,77 | -0,09 | 0,59 | 0,53 | 0,02 | 0,07 | 0,6 |
| **ski_ii** | -0,12 | 0,11 | 0,04 | 0,09 | 0,04 | 0,06 | 0,08 | 0,05 | 0,07 |
| **lxr-623** | -0,12 | 0,06 | 0 | 0,02 | -0,01 | -0,02 | 0,03 | -0,07 | -0,09 |
| **febuxostat** | -0,12 | -0,02 | 0 | -0,02 | -0,08 | -0,09 | -0,06 | 0,03 | -0,05 |
| **calcipotriol** | -0,12 | -0,02 | -0,07 | -0,04 | -0,04 | -0,09 | -0,01 | 0,12 | -0,11 |
| **akr1c3-in-1** | -0,12 | -0,11 | -0,03 | 0,04 | -0,04 | 0,15 | 0,02 | 0,01 | -0,14 |
| **auda** | -0,12 | -0,13 | -0,07 | 0,01 | -0,03 | -0,04 | 0 | 0,02 | -0,04 |
| **rofecoxib** | -0,12 | -0,15 | -0,03 | -0,01 | 0,03 | -0,03 | -0,03 | 0,08 | -0,02 |
| **epz-6438** | -0,13 | 0,38 | -0,12 | -0,04 | -0,07 | 0 | -0,02 | -0,04 | -0,04 |
| **bms309403** | -0,13 | 0,17 | -0,03 | -0,02 | 0,03 | -0,04 | -0,06 | 0 | -0,06 |
| **chembl383736** | -0,13 | 0,1 | 0,04 | 0 | -0,01 | 0 | -0,03 | 0,05 | -0,02 |
| **bicifadine** | -0,13 | 0,08 | 0,09 | -0,06 | -0,04 | -0,02 | -0,02 | 0,02 | -0,05 |
| **tandutinib** | -0,13 | 0,05 | 0 | -0,05 | -0,03 | -0,04 | 0,01 | -0,07 | 0 |
| **h-1152** | -0,13 | 0,01 | 0,05 | -0,04 | 0,03 | -0,05 | 0,02 | 0,14 | -0,02 |
| **eniporide** | -0,13 | 0,01 | -0,12 | -0,04 | -0,05 | -0,02 | 0,04 | 0,04 | 0 |
| **tacrolimus** | -0,13 | -0,02 | 0,02 | -0,04 | -0,01 | -0,02 | 0,03 | 0,05 | 0,01 |
| **nb-598** | -0,13 | -0,02 | 0,05 | -0,01 | -0,06 | -0,03 | 0,03 | -0,08 | -0,06 |
| **dantrolene** | -0,13 | -0,03 | -0,07 | -0,05 | -0,2 | -0,11 | 0,04 | 0,04 | -0,08 |
| **bbl003126** | -0,13 | -0,11 | 0,08 | -0,16 | 0,04 | -0,06 | -0,01 | -0,01 | 0 |
| **l-nil** | -0,13 | -0,15 | 0,01 | -0,01 | -0,07 | 0,01 | -0,09 | 0 | -0,06 |
| **gsk189254a** | -0,13 | -0,15 | -0,02 | 0,01 | 0,05 | -0,02 | 0,05 | 0,06 | 0,01 |
| **nvp-bsk805** | -0,13 |  | -0,05 | 0,07 | 0,05 | 0,01 | 0,01 | -0,13 | -0,02 |
| **neurokinin_a** | -0,13 |  | -0,09 | -0,06 | -0,06 | -0,06 | -0,06 | 0,06 | -0,06 |
| **bay_60-7550** | -0,13 |  | 0,1 | 0,06 | -0,04 | 0,07 | -0,02 | 0,08 | 0,06 |
| **resmetiromв** | -0,14 | 0,1 | 0,03 | -0,01 | -0,01 | -0,1 | -0,02 | 0,05 | -0,05 |
| **gr79236** | -0,14 | 0 | 0,06 | -0,03 | 0,02 | -0,16 | -0,02 | 0,02 | -0,08 |
| **seratrodast** | -0,14 | -0,01 | 0,13 | 0,06 | -0,09 | -0,02 | -0,03 | 0,1 | 0 |
| **tasimelteon** | -0,14 | -0,03 | 0 | -0,04 | 0 | -0,04 | -0,02 | 0,04 | -0,06 |
| **stk235555** | -0,14 | -0,03 | 0,03 | -0,12 | -0,02 | 0 | -0,01 | -0,01 | 0 |
| **nifedipine** | -0,14 | -0,04 | 0 | -0,07 | 0,03 | -0,07 | -0,03 | -0,04 | -0,06 |
| **wz811** | -0,14 | -0,05 | -0,1 | -0,04 | 0,02 | 0,11 | 0,02 | 0,07 | 0,05 |
| **rifampicin** | -0,14 | -0,1 | -0,01 | 0,01 | 0,01 | -0,01 | -0,03 | -0,1 | -0,03 |
| **acamprosate** | -0,14 | -0,12 | 0,04 | 0,01 | -0,13 | -0,07 | -0,01 | -0,13 | -0,05 |
| **terazosin** | -0,14 | -0,13 | 0 | 0,01 | -0,01 | -0,04 | -0,05 | 0,05 | 0 |
| **7_8-dihydroxyflavone** | -0,14 | -0,18 | 0,03 | -0,02 | 0,03 | 0,03 | 0,03 | 0 | -0,01 |
| **bml-277** | -0,14 | -0,19 | 0,05 | 0,06 | 0,11 | 0,11 | 0,02 | 0,05 | 0,06 |
| **osilodrostat** | -0,14 | -0,19 | 0,06 | -0,03 | 0,01 | -0,04 | 0,05 | 0,02 | -0,05 |
| **cid-2745687** | -0,14 | -0,29 | -0,03 | -0,02 | 0,03 | -0,03 | 0 | -0,01 | -0,02 |
| **hx_531** | -0,14 | -0,31 | -0,1 | -0,08 | 0,01 | 0,29 | 0,01 | -0,4 | -0,13 |
| **gandotinib** | -0,14 | -0,38 | 0,12 | 0,04 | 0,14 | 0,05 | 0,09 | 0,1 | 0,17 |
| **debio-1347** | -0,15 | 0,21 | 0,2 | 0,1 | -0,04 | 0,06 | 0,1 | 0 | 0,02 |
| **prt062607** | -0,15 | 0,14 | 0,03 | 0,12 | 0,1 | 0,06 | 0,06 | -0,02 | 0,02 |
| **gefitinib** | -0,15 | 0,12 | -0,06 | 0,01 | 0 | 0,02 | -0,03 | -0,12 | 0,01 |
| **mre-269** | -0,15 | 0,07 | 0,06 | 0,01 | -0,07 | 0 | -0,02 | -0,04 | 0,02 |
| **stl304557** | -0,15 | 0,03 | -0,09 | -0,06 | -0,02 | 0 | -0,04 | 0,03 | -0,02 |
| **prucalopride** | -0,15 | 0,03 | 0 | -0,01 | 0,03 | -0,03 | 0 | 0,03 | -0,06 |
| **6-mercaptopurine** | -0,15 | 0,02 | 0,23 | 0,13 | -0,13 | -0,03 | -0,01 | 0,07 | -0,07 |
| **zm_336372** | -0,15 | 0 | -0,22 | -0,09 | 0 | 0 | -0,03 | -0,03 | -0,06 |
| **sb_225002** | -0,15 | -0,02 | 0,38 | 0,17 | 0,09 | 0,26 | 0,11 | -0,1 | -0,02 |
| **nsc43673** | -0,15 | -0,09 | -0,05 | 0,03 | -0,04 | -0,04 | 0,01 | 0,01 | -0,05 |
| **quizartinib** | -0,15 | -0,09 | 0 | -0,03 | 0,06 | 0,07 | -0,01 | 0,05 | 0 |
| **uk_383367** | -0,15 | -0,35 | 0,04 | -0,02 | 0,07 | -0,01 | -0,01 | 0,12 | 0,01 |
| **gnf-5** | -0,16 | 0,1 | -0,06 | 0,01 | -0,04 | -0,02 | -0,02 | 0,2 | 0,08 |
| **l-mimosine** | -0,16 | 0,07 | 0,01 | -0,01 | 0,02 | 0,06 | 0,01 | 0,04 | 0,03 |
| **agi-6780** | -0,16 | 0,03 | -0,04 | -0,01 | -0,02 | -0,06 | -0,03 | 0,03 | -0,07 |
| **tofa** | -0,16 | -0,09 | -0,01 | 0,01 | 0,03 | 0,03 | 0,01 | -0,02 | -0,01 |
| **paricalcitol** | -0,16 | -0,24 | -0,01 | 0 | 0,07 | 0 | 0,06 | 0,07 | 0,03 |
| **fedratinib** | -0,17 | 0,63 | -0,24 | -0,03 | 0,16 | 0,05 | -0,06 | -0,03 | 0,25 |
| **jp1302** | -0,17 | 0,26 | 0,04 | 0,27 | 0,1 | 0,31 | 0,2 | -0,03 | 0,18 |
| **agk2** | -0,17 | 0,26 | 0,05 | 0,02 | -0,08 | -0,13 | 0,02 | -0,27 | 0,05 |
| **olodaterol** | -0,17 | 0,08 | -0,01 | 0,05 | 0,06 | -0,04 | -0,05 | 0,01 | 0,04 |
| **hms3745g19** | -0,17 | 0,03 | 0,06 | 0,01 | -0,01 | -0,03 | -0,02 | -0,1 | -0,02 |
| **bcx_1470** | -0,17 | 0,02 | -0,01 | -0,02 | -0,03 | 0 | -0,03 | 0,03 | 0 |
| **tgx-221** | -0,17 | -0,04 | -0,01 | -0,06 | -0,03 | 0,01 | -0,03 | -0,03 | -0,02 |
| **maraviroc** | -0,17 |  | -0,01 | 0 | -0,07 | -0,12 | 0,04 | -0,1 | -0,07 |
| **orteronel** | -0,18 | 0,2 | 0,06 | -0,03 | -0,03 | -0,07 | 0,01 | -0,06 | -0,04 |
| **abiraterone2** | -0,18 | 0,07 | -0,05 | -0,01 | 0,01 | 0,04 | 0 | -0,01 | -0,02 |
| **at_406** | -0,18 | 0,01 | 0,01 | 0,02 | 0 | 0,03 | -0,03 | -0,02 | -0,03 |
| **ibrutinib2** | -0,18 | 0,01 | -0,02 | -0,01 | -0,04 | 0,05 | 0,02 | 0,08 | 0,04 |
| **iu1** | -0,18 | -0,02 | 0,07 | 0,04 | -0,09 | 0,01 | 0,01 | 0,06 | -0,04 |
| **sch_58261** | -0,18 | -0,03 | 0 | 0,01 | 0,04 | -0,04 | -0,09 | -0,01 | -0,03 |
| **veliparibв** | -0,18 | -0,06 | -0,01 | -0,07 | 0 | 0 | 0,07 | -0,07 | -0,02 |
| **sb_218795** | -0,18 | -0,1 | 0 | -0,1 | 0,03 | 0,07 | 0,05 | 0 | -0,01 |
| **irak-1-4_inhibitor_i** | -0,19 | 0 | 0,15 | -0,07 | 0,09 | 0 | 0,03 | -0,05 | -0,05 |
| **ic-87114** | -0,19 | -0,01 | -0,06 | -0,11 | -0,08 | 0 | -0,05 | 0 | -0,06 |
| **sitaxsentan** | -0,19 | -0,02 | 0,02 | 0,01 | 0 | -0,1 | -0,08 | -0,02 | -0,02 |
| **apd668** | -0,19 | -0,05 | -0,05 | 0,03 | -0,02 | -0,01 | -0,03 | 0,05 | 0,04 |
| **dabigatran** | -0,19 | -0,1 | -0,05 | 0,04 | 0,03 | -0,01 | 0,02 | -0,02 | 0 |
| **tr-14035** | -0,19 | -0,16 | -0,01 | -0,04 | 0,1 | 0,01 | -0,02 | 0,04 | -0,03 |
| **berzosertib** | -0,19 | -0,19 | -0,1 | 0,01 | 0,02 | 0,05 | -0,02 | 0 | 0,04 |
| **ml365** | -0,19 | -0,51 | -0,07 | -0,02 | 0 | 0 | -0,05 | 0,01 | 0 |
| **bexarotene** | -0,2 | 0,13 | -0,03 | 0 | 0,07 | -0,05 | -0,03 | -0,08 | -0,06 |
| **ursodiol** | -0,2 | 0,09 | -0,06 | 0,01 | 0,01 | 0,17 | 0,21 | -0,06 | -0,04 |
| **gsk_650394** | -0,2 | -0,01 | 0,05 | -0,04 | -0,07 | 0,04 | -0,01 | -0,05 | 0 |
| **tanomastat** | -0,2 | -0,04 | -0,02 | 0,02 | -0,1 | -0,01 | -0,03 | 0,01 | -0,02 |
| **ly2090314** | -0,2 | -0,13 | 0,1 | 0,04 | 0,09 | 0,07 | 0,04 | 0 | 0,05 |
| **unc2881** | -0,21 | 0,09 | 0,09 | 0,02 | 0,11 | 0,04 | 0,02 | 0,1 | 0,01 |
| **nisoldipine** | -0,21 | 0,02 | -0,04 | -0,03 | -0,01 | 0,04 | -0,03 | 0,01 | -0,01 |
| **cct241533** | -0,21 | -0,02 | 0,02 | -0,07 | 0,01 | 0 | 0,03 | 0,02 | -0,06 |
| **olanzapine** | -0,21 | -0,18 | 0,03 | -0,07 | 0 | 0 | 0,01 | -0,02 | -0,02 |
| **ibrutinib1** | -0,22 | -0,08 | 0,02 | 0,02 | 0,01 | -0,03 | 0,07 | 0,01 | 0,03 |
| **testolactone** | -0,22 | -0,12 | 0,04 | 0,09 | -0,01 | -0,01 | -0,03 | 0,07 | 0 |
| **idasanutlin** | -0,22 | -0,22 | 0,06 | 0,19 | 0,03 | 0,05 | 0,08 | 0,07 | 0,11 |
| **ro_48-8071** | -0,22 | -0,28 | -0,04 | -0,09 | 0,01 | 0 | -0,03 | 0,06 | 0,05 |
| **tubastatin_a** | -0,23 | 0,11 | 0,02 | -0,07 | 0 | -0,04 | -0,05 | 0,04 | -0,01 |
| **tmn_355** | -0,23 | 0 | 0,05 | -0,01 | -0,01 | 0 | -0,03 | -0,02 | 0,01 |
| **palonosetron** | -0,23 | -0,04 | -0,03 | -0,05 | 0,11 | -0,01 | 0,08 | 0 | -0,04 |
| **ivacaftor** | -0,23 | -0,23 | -0,15 | 0,03 | 0,09 | -0,03 | -0,06 | -0,18 | -0,02 |
| **cilengitide** | -0,23 | -0,27 | -0,19 | 0,04 | -0,1 | 0,13 | -0,13 | -0,19 | -0,05 |
| **histamine** | -0,24 | -0,04 | 0,03 | -0,02 | -0,08 | -0,06 | -0,06 | -0,03 | -0,04 |
| **gw842166x** | -0,24 | -0,26 | 0,15 | 0 | -0,03 | 0,08 | 0,01 | -0,09 | 0,01 |
| **anagrelide** | -0,25 | 0,27 | 0,03 | 0,01 | 0,04 | -0,02 | 0,01 | 0,04 | -0,01 |
| **tacrine** | -0,25 | 0,06 | -0,09 | -0,09 | 0,06 | -0,04 | 0 | -0,03 | -0,06 |
| **chembl569227** | -0,25 | -0,06 | 0,26 | 0 | 0,07 | -0,01 | -0,04 | 0 | -0,16 |
| **fk866** | -0,25 | -0,08 | 0,01 | 0,05 | 0 | -0,07 | 0,01 | 0,03 | -0,03 |
| **hfi-142** | -0,25 | -0,32 | 0,02 | -0,05 | 0 | -0,07 | -0,07 | -0,08 | 0 |
| **bcatc-in2** | -0,26 | 0,27 | 0,01 | -0,06 | 0,04 | -0,02 | 0 | -0,11 | 0 |
| **7-hydroxycoumarine** | -0,26 | -0,09 | 0 | -0,02 | -0,02 | -0,02 | 0,02 | -0,07 | -0,06 |
| **dimethylfraxetin** | -0,26 | -0,11 | -0,07 | -0,1 | -0,02 | -0,02 | -0,08 | -0,01 | -0,08 |
| **finasteride** | -0,26 | -0,19 | 0,04 | 0 | 0,01 | 0,02 | -0,02 | 0 | 0,05 |
| **fipi_hcl** | -0,27 | 0,12 | 0,01 | 0,08 | -0,03 | 0,03 | 0 | -0,15 | -0,02 |
| **defactinib** | -0,27 | 0,09 | -0,04 | -0,09 | -0,06 | 0,01 | 0,01 | 0,08 | 0 |
| **ns_3763** | -0,27 | -0,12 | -0,05 | -0,08 | -0,02 | -0,06 | -0,06 | -0,11 | -0,02 |
| **g-1** | -0,28 | 0,21 | 0,05 | 0,13 | 0,22 | 0,11 | 0,15 | 0,07 | 0,23 |
| **bombesin** | -0,28 |  | 0 | 0,03 | -0,01 | -0,04 | 0,03 | -0,05 | -0,02 |
| **7_3_4-thif** | -0,29 | -0,04 | -0,08 | 0,02 | -0,04 | 0,04 | -0,02 | -0,08 | 0 |
| **chembl513147** | -0,29 | -0,2 | 0,02 | -0,26 | 0,14 | 0,01 | -0,01 | -0,28 | -0,12 |
| **smi-16a** | -0,3 | 0,02 | -0,03 | -0,07 | -0,08 | -0,03 | 0 | 0,07 | -0,01 |
| **indapamide** | -0,3 | -0,07 | 0,04 | -0,05 | 0 | -0,07 | -0,02 | 0,01 | -0,02 |
| **stx-0119** | -0,31 | -0,04 | -0,01 | -0,05 | -0,04 | -0,01 | -0,02 | 0,02 | -0,01 |
| **mrs_2578** | -0,32 | -0,12 | 0,06 | 0,03 | 0,04 | 0,05 | 0 | -0,06 | 0,03 |
| **entinostat** | -0,33 | 1,38 | -0,21 | -0,2 | 0,06 | -0,07 | -0,17 | -0,12 | -0,14 |
| **ethoxzolamide** | -0,33 |  | 0,04 | 0 | 0,02 | -0,02 | -0,01 | 0,05 | 0,01 |
| **aripiprazole** | -0,34 | 0,1 | -0,01 | 0,05 | 0,05 | 0 | -0,03 | -0,03 | 0,01 |
| **vatalanib** | -0,34 | -0,15 | 0,05 | 0,08 | -0,02 | 0,03 | 0,04 | 0,02 | 0,07 |
| **otssp167** | -0,35 | -0,12 | 0,03 | 0,29 | -0,09 | 0,29 | 0,01 | -0,16 | 0,17 |
| **tazarotene1** | -0,36 | -0,09 | 0,01 | -0,02 | 0,01 | -0,02 | -0,03 | 0,03 | 0 |
| **nms-873** | -0,36 | -0,37 | -0,11 | -0,07 | 0,05 | 0,15 | 0,01 | -0,16 | -0,12 |
| **anacetrapib** | -0,37 | 0,01 | 0,04 | 0 | 0 | 0 | -0,03 | -0,05 | -0,02 |
| **stk841699** | -0,37 | -0,06 | 0,08 | 0,06 | -0,01 | 0,07 | 0,01 | 0,01 | -0,01 |
| **qnz** | -0,37 | -0,07 | -0,11 | -0,05 | -0,02 | -0,02 | 0 | -0,04 | -0,04 |
| **gsk2578215** | -0,37 | -0,22 | 0 | -0,07 | -0,04 | -0,02 | -0,03 | -0,03 | -0,08 |
| **hpgds-in1** | -0,38 | 0,01 | -0,03 | -0,02 | -0,06 | -0,08 | -0,05 | 0,04 | -0,04 |
| **bix_01294** | -0,38 | -0,03 | 0,11 | -0,02 | 0,02 | 0,15 | 0,08 | -0,14 | -0,07 |
| **enzastaurin** | -0,38 | -0,07 | -0,01 | -0,05 | 0,02 | -0,06 | -0,04 | -0,04 | -0,07 |
| **bi_2536** | -0,39 | 0,48 | -0,1 | -0,14 | -0,09 | 0 | -0,1 | -0,09 | -0,02 |
| **rvx-208** | -0,39 | 0,08 | 0,02 | 0,02 | 0 | 0,02 | -0,01 | -0,04 | -0,03 |
| **chembl2041153** | -0,39 | -0,11 | -0,03 | -0,03 | -0,01 | -0,05 | -0,03 | -0,04 | -0,02 |
| **sb_216763** | -0,39 | -0,25 | 0,03 | -0,1 | 0,09 | 0,09 | 0,04 | 0,08 | 0 |
| **mrs_1754** | -0,4 | -0,05 | 0,01 | 0 | -0,04 | 0,05 | 0,05 | 0,07 | -0,02 |
| **as_1517499** | -0,42 | 0,22 | 0,14 | 0,31 | 0,1 | 0,25 | 0,17 | -0,01 | 0,28 |
| **jq-1** | -0,44 |  | -0,08 | -0,1 | -0,05 | -0,02 | -0,09 | -0,07 | -0,06 |
| **olopatadine** | -0,46 |  | 0,05 | -0,04 | 0,01 | 0 | 0,05 | -0,02 | -0,05 |
| **mbx-2982** | -0,48 | 0,02 | 0,03 | -0,02 | -0,02 | -0,03 | 0,01 | 0,03 | 0,03 |
| **l-valyl-l-phenylalanine** | -0,51 | 1,15 | -0,1 | -0,16 | 0,07 | -0,03 | -0,17 | -0,1 | -0,15 |
| **cp-673451** | -0,52 | -0,15 | -0,01 | -0,09 | -0,03 | 0 | 0,02 | -0,05 | -0,02 |
| **quisinostat** | -0,6 | 0,5 | -0,19 | -0,3 | -0,01 | -0,01 | -0,33 | -0,19 | -0,16 |
| **rgfp966** | -0,65 | 1,12 | -0,07 | -0,17 | 0,1 | 0,02 | -0,07 | -0,02 | -0,1 |
| **nexturastat_a** | -0,73 | 1,23 | 0,03 | -0,15 | 0,06 | -0,05 | -0,24 | -0,37 | -0,06 |
| **rg2833** | -0,79 |  | -0,2 | -0,26 | 0,04 | -0,11 | -0,17 | -0,03 | -0,22 |
| **vorinostat** | -0,94 | 0,9 | -0,22 | -0,21 | 0,26 | -0,02 | -0,22 | -0,23 | -0,15 |
| **brequinar** |  | 1 | 0,08 | 0,22 | 0,14 | 0,23 | 0,18 | 0,11 | 0,2 |
| **tadalafil** |  | 0,81 | -0,01 | -0,03 | 0,06 | -0,09 | 0,04 | 0,06 | -0,04 |
| **mi-773** |  | 0,43 | 0,1 | 0,23 | 0,11 | 0,27 | 0,09 | 0,27 | 0,19 |
| **gw4064** |  | 0,38 | 0,02 | -0,01 | 0 | 0 | -0,05 | 0,05 | 0,01 |
| **sapropterinв** |  | 0,35 | -0,06 | -0,06 | 0,07 | 0,11 | 0,12 | 0,01 | 0 |
| **az20** |  | 0,34 | -0,16 | 0,29 | 0,01 | 0,08 | 0,06 | 0,02 | 0,12 |
| **pp_1** |  | 0,31 | -0,05 | -0,01 | -0,03 | 0,12 | 0,05 | 0,02 | 0,04 |
| **icg-001** |  | 0,3 | -0,13 | 0,12 | 0,04 | 0,11 | 0,12 | 0,1 | 0,1 |
| **gsk-1070916** |  | 0,3 | -0,14 | 0,17 | -0,17 | 0,02 | 0,19 | -0,01 | 0,15 |
| **ly_364947** |  | 0,29 | -0,05 | 0,03 | 0 | -0,08 | -0,07 | -0,07 | -0,08 |
| **ml349** |  | 0,29 | -0,02 | 0,07 | 0,03 | -0,02 | -0,03 | 0,11 | -0,03 |
| **sb_334867** |  | 0,25 | 0,05 | -0,05 | 0 | 0,1 | 0,08 | 0,02 | 0,04 |
| **lpa2_an1** |  | 0,23 | 0,05 | 0,07 | 0 | 0,08 | 0,04 | -0,04 | 0,02 |
| **nolatrexed** |  | 0,23 | 0,08 | 0,12 | 0,05 | 0,02 | 0,07 | 0,05 | 0,01 |
| **cetrorelix** |  | 0,2 | -0,08 | -0,01 | -0,06 | 0,1 | -0,02 | 0,11 | 0,03 |
| **alfuzosin** |  | 0,2 | 0,03 | 0,05 | -0,04 | -0,13 | -0,09 | 0,05 | -0,13 |
| **blonanserin** |  | 0,17 | 0 | 0,01 | 0,02 | -0,12 | 0,01 | -0,05 | 0,01 |
| **lomeguatrib** |  | 0,16 | -0,03 | -0,02 | -0,02 | 0,19 | 0 | 0,04 | -0,05 |
| **cb-839** |  | 0,16 | 0,02 | -0,03 | 0,09 | 0,02 | 0,03 | -0,05 | -0,05 |
| **gsk4112** |  | 0,15 | 0,01 | -0,04 | 0,02 | 0,02 | 0,01 | -0,03 | -0,02 |
| **2_2-biquinoline** |  | 0,15 | 0,02 | 0,02 | 0,02 | -0,01 | 0 | -0,02 | 0,01 |
| **ca-074** |  | 0,15 | -0,02 | -0,03 | -0,02 | -0,11 | -0,06 | -0,03 | -0,01 |
| **ml218** |  | 0,15 | -0,05 | 0 | -0,05 | -0,04 | -0,07 | -0,04 | -0,03 |
| **4egi-1** |  | 0,13 | 0,01 | -0,02 | 0,01 | 0,04 | 0,02 | -0,08 | 0 |
| **cb_13** |  | 0,13 | 0,02 | -0,01 | -0,04 | 0,07 | -0,05 | 0,05 | -0,07 |
| **pha_767491** |  | 0,13 | 0,34 | 0,28 | 0,13 | 0,34 | 0,22 | 0,22 | 0,35 |
| **ascomycin** |  | 0,12 | -0,06 | 0,1 | 0,03 | 0,06 | -0,01 | -0,03 | 0,09 |
| **sch_530348** |  | 0,11 | -0,01 | -0,02 | -0,03 | -0,01 | -0,03 | -0,02 | -0,02 |
| **bx_471** |  | 0,11 | -0,06 | -0,07 | 0 | -0,01 | 0 | -0,14 | 0,04 |
| **blz-945** |  | 0,11 | -0,03 | 0,01 | 0,03 | -0,01 | -0,06 | 0,08 | -0,01 |
| **mk_886** |  | 0,11 | 0,04 | 0,08 | 0,04 | 0,06 | 0,02 | -0,08 | 0,02 |
| **alectinib** |  | 0,11 | -0,01 | -0,08 | 0 | 0,03 | 0,02 | 0,04 | 0,08 |
| **hc_030031** |  | 0,09 | -0,02 | -0,02 | -0,06 | -0,02 | -0,05 | -0,01 | 0 |
| **anastrozole** |  | 0,09 | -0,01 | 0,02 | -0,03 | -0,17 | -0,05 | 0,02 | -0,04 |
| **jw_480** |  | 0,08 | 0,01 | 0,03 | -0,03 | 0,02 | 0,03 | 0,14 | 0,04 |
| **pentadecanoyl_ea** |  | 0,08 | -0,08 | -0,01 | 0 | -0,05 | -0,06 | -0,08 | 0 |
| **xct_790** |  | 0,08 | -0,03 | 0,08 | -0,01 | 0 | 0,02 | 0,05 | -0,03 |
| **bromfenac** |  | 0,08 | 0 | -0,06 | -0,02 | -0,09 | -0,12 | 0 | -0,07 |
| **dipyridamole** |  | 0,08 | -0,13 | 0,01 | -0,11 | -0,23 | -0,06 | 0,07 | -0,05 |
| **eipa** |  | 0,07 | 0,03 | 0,04 | 0,05 | -0,02 | 0,06 | 0,02 | 0,06 |
| **4-nitropiazthiole** |  | 0,07 | 0,02 | 0,11 | -0,08 | 0,05 | 0,04 | 0,1 | 0,18 |
| **salmeterol** |  | 0,07 | -0,03 | 0,05 | 0,07 | 0,19 | 0,12 | -0,03 | 0,06 |
| **undecanoic_acid** |  | 0,06 | 0 | -0,03 | -0,03 | -0,03 | -0,02 | 0,01 | 0 |
| **pirenzepine** |  | 0,06 | 0,04 | -0,08 | 0,02 | 0,09 | 0,09 | 0 | 0,1 |
| **raloxifene** |  | 0,06 | -0,08 | -0,05 | -0,04 | 0,02 | -0,03 | -0,09 | 0,03 |
| **acitretin** |  | 0,06 | -0,06 | -0,07 | -0,06 | 0,03 | 0,01 | 0,02 | 0,03 |
| **rislenemdaz** |  | 0,06 | -0,01 | -0,06 | 0 | 0,05 | -0,02 | 0,02 | 0,01 |
| **mardepodect** |  | 0,06 | 0,26 | -0,03 | -0,11 | -0,15 | -0,05 | 0,01 | -0,01 |
| **bq-788** |  | 0,05 | -0,02 | 0,03 | 0,06 | 0,01 | -0,05 | -0,06 | -0,01 |
| **ralfinamide** |  | 0,04 | 0 | 0,01 | -0,15 | 0 | 0,01 | 0,01 | 0,02 |
| **mk-1775** |  | 0,04 | -0,04 | 0,05 | 0,01 | 0,02 | 0 | 0,03 | 0,12 |
| **pamidronic_acid** |  | 0,04 | -0,02 | -0,09 | 0,12 | 0,1 | 0,03 | 0 | 0,04 |
| **fluoxymesterone** |  | 0,04 | 0,01 | 0,03 | 0,03 | 0,12 | 0,07 | -0,03 | 0,05 |
| **sr_142948** |  | 0,03 | 0,02 | -0,01 | -0,03 | -0,09 | 0 | 0,18 | -0,07 |
| **lubiprostone** |  | 0,03 | 0,03 | -0,01 | -0,06 | -0,03 | -0,02 | 0,07 | -0,11 |
| **tofacitinib** |  | 0,03 | 0,04 | -0,04 | 0,07 | -0,01 | 0,01 | 0,17 | 0,01 |
| **metyrapone** |  | 0,03 | -0,02 | 0,07 | -0,01 | -0,12 | -0,07 | -0,2 | -0,09 |
| **ml347** |  | 0,03 | -0,07 | -0,02 | -0,03 | -0,05 | 0 | 0,01 | -0,01 |
| **dinoprost** |  | 0,03 | -0,02 | -0,06 | 0 | 0,01 | 0,04 | -0,03 | 0,02 |
| **cysmethynil** |  | 0,02 | 0 | -0,1 | -0,03 | 0,01 | -0,02 | -0,05 | -0,07 |
| **nsc735380** |  | 0,02 | -0,07 | 0,11 | -0,13 | 0,07 | -0,01 | -0,01 | 0,05 |
| **cp_94253** |  | 0,02 | -0,02 | 0,01 | -0,02 | -0,05 | -0,02 | 0,08 | 0,01 |
| **pnu-120596** |  | 0,02 | -0,03 | -0,05 | 0,09 | 0 | -0,04 | 0,04 | -0,04 |
| **cyanuric_chloride** |  | 0,01 | -0,04 | -0,03 | -0,06 | -0,02 | 0,01 | -0,02 | 0 |
| **4_4-dichlorobenzil** |  | 0 | 0,02 | -0,01 | -0,01 | 0,02 | 0 | -0,08 | -0,01 |
| **tafamidis** |  | 0 | -0,01 | -0,06 | -0,06 | -0,05 | -0,06 | 0,05 | -0,03 |
| **senicapoc** |  | 0 | -0,01 | 0,03 | 0,05 | -0,07 | 0,01 | -0,02 | -0,02 |
| **ml-265** |  | -0,01 | 0,01 | 0 | 0 | -0,01 | -0,01 | -0,04 | 0 |
| **ezatiostat** |  | -0,01 | 0,04 | 0 | 0,02 | 0,05 | -0,03 | -0,06 | -0,02 |
| **sp2509** |  | -0,01 | 0,01 | -0,02 | 0,01 | 0,03 | 0,02 | -0,03 | 0,03 |
| **fenoldopam** |  | -0,01 | 0 | -0,11 | -0,02 | 0,05 | -0,01 | -0,03 | 0,02 |
| **2-pmpa** |  | -0,01 | 0,19 | 0,03 | -0,17 | -0,02 | 0,01 | -0,05 | -0,05 |
| **ly334370** |  | -0,01 | -0,03 | 0,06 | -0,11 | -0,13 | -0,12 | -0,02 | -0,02 |
| **cgp77675** |  | -0,02 | 0,01 | 0,04 | 0,02 | 0,01 | 0 | 0,04 | 0,04 |
| **isoproterenol** |  | -0,02 | -0,1 | -0,04 | 0 | -0,01 | 0 | -0,1 | -0,06 |
| **gw_3965** |  | -0,02 | -0,05 | -0,02 | -0,04 | -0,04 | 0,05 | -0,11 | -0,04 |
| **epz004777** |  | -0,02 | 0,12 | -0,09 | 0,07 | -0,06 | -0,01 | -0,03 | 0,03 |
| **glycyrrhizic_acid** |  | -0,03 | 0 | -0,03 | 0,11 | 0 | -0,01 | 0,15 | 0 |
| **cilostamide** |  | -0,03 | -0,04 | -0,07 | 0,03 | 0,1 | 0,13 | 0 | 0,09 |
| **metolazone** |  | -0,03 | 0,01 | -0,06 | 0,02 | -0,01 | -0,01 | -0,03 | 0,01 |
| **stiripentol** |  | -0,03 | 0,01 | 0,04 | -0,02 | -0,02 | -0,04 | -0,01 | -0,03 |
| **pf-8380** |  | -0,04 | -0,01 | 0,02 | 0 | 0,01 | -0,01 | -0,01 | 0 |
| **chloro-l-alanine** |  | -0,04 | -0,07 | -0,02 | 0,11 | 0,08 | 0,09 | 0,04 | 0,04 |
| **aca** |  | -0,05 | 0,03 | 0,02 | -0,04 | 0,01 | -0,01 | 0,03 | -0,04 |
| **benethamine** |  | -0,05 | -0,02 | -0,01 | 0,06 | -0,01 | -0,01 | 0,07 | 0 |
| **4-methylhistamine** |  | -0,05 | -0,02 | 0,04 | 0,02 | 0,02 | 0,01 | -0,04 | -0,02 |
| **a_803467** |  | -0,05 | 0,02 | 0,02 | -0,06 | -0,09 | -0,03 | 0,02 | -0,14 |
| **nitisinone** |  | -0,06 | 0,04 | -0,04 | 0,01 | -0,04 | 0,01 | -0,02 | -0,06 |
| **sildenafil** |  | -0,06 | -0,04 | -0,01 | 0 | 0,01 | -0,02 | -0,01 | 0 |
| **hy-50713** |  | -0,06 | 0,03 | 0,1 | -0,13 | 0,12 | 0,04 | -0,01 | 0,07 |
| **w-13_hcl** |  | -0,06 | 0,04 | -0,03 | -0,02 | -0,04 | -0,01 | 0,07 | 0,02 |
| **cbl0137** |  | -0,06 | 0,17 | 0,19 | 0,27 | 0,12 | 0,22 | 0,05 | 0,35 |
| **sr_3335** |  | -0,07 | 0,05 | -0,07 | -0,02 | -0,05 | -0,08 | -0,03 | -0,07 |
| **bms_754807** |  | -0,07 | 0 | 0,07 | 0,01 | -0,01 | 0,01 | -0,08 | 0,03 |
| **parecoxib** |  | -0,07 | -0,1 | -0,01 | -0,06 | -0,07 | -0,1 | -0,07 | 0 |
| **hmb-val-ser-leu-ve** |  | -0,08 | -0,11 | -0,03 | -0,02 | 0,16 | -0,06 | -0,06 | 0 |
| **zm_241385** |  | -0,08 | -0,01 | -0,1 | 0,01 | -0,07 | 0,01 | -0,06 | -0,05 |
| **azd-5438** |  | -0,08 | 0,02 | 0,43 | 0,24 | 0,34 | 0,32 | 0,19 | 0,39 |
| **iclaprim** |  | -0,09 | 0 | 0,09 | -0,05 | -0,13 | -0,08 | 0,08 | -0,08 |
| **tiplaxtinin** |  | -0,1 | -0,02 | -0,03 | -0,03 | -0,05 | -0,08 | 0,08 | -0,03 |
| **cay10550** |  | -0,1 | -0,04 | -0,12 | -0,05 | -0,08 | -0,07 | -0,01 | -0,13 |
| **tak-875** |  | -0,1 | -0,09 | 0,07 | -0,01 | -0,04 | -0,19 | 0 | -0,07 |
| **basimglurant** |  | -0,1 | 0 | -0,05 | -0,06 | 0,01 | 0,01 | -0,14 | -0,05 |
| **huperzine_a** |  | -0,1 | -0,03 | -0,01 | 0,01 | 0,04 | -0,01 | -0,1 | 0 |
| **ly2801653** |  | -0,1 | 0 | 0,01 | -0,05 | 0,03 | -0,03 | -0,19 | -0,03 |
| **birabresib** |  | -0,1 | 0,33 | -0,11 | -0,08 | -0,2 | -0,05 | -0,13 | -0,08 |
| **triclabendazole** |  | -0,11 | 0,04 | -0,07 | -0,03 | 0,04 | 0,03 | -0,04 | -0,05 |
| **pyrene** |  | -0,11 | 0,01 | -0,09 | 0,05 | 0,09 | 0,08 | 0 | 0,04 |
| **way_170523** |  | -0,12 | 0,01 | 0 | 0,06 | -0,03 | 0 | -0,02 | 0,03 |
| **perindopril** |  | -0,12 | -0,05 | 0,04 | -0,04 | 0,02 | -0,04 | -0,01 | 0,01 |
| **cloprostenol** |  | -0,12 | 0,22 | 0,08 | -0,13 | -0,21 | -0,06 | 0,08 | -0,08 |
| **pf_04447943** |  | -0,13 | -0,03 | -0,01 | 0 | -0,04 | 0,01 | 0,15 | -0,03 |
| **plx-4720** |  | -0,13 | 0,15 | -0,14 | -0,19 | 0 | 0,01 | -0,08 | -0,06 |
| **pf-04418948** |  | -0,15 | -0,02 | -0,06 | -0,02 | 0,01 | 0 | -0,01 | 0 |
| **trihydroxyisoflavone** |  | -0,15 | 0,07 | -0,02 | 0,03 | -0,16 | -0,02 | 0,1 | -0,04 |
| **compound-1a** |  | -0,16 | 0,08 | -0,06 | 0,01 | -0,04 | 0,05 | -0,15 | 0,03 |
| **racecadotril** |  | -0,16 | -0,02 | 0 | 0,06 | -0,01 | 0,03 | 0,02 | 0 |
| **mubritinib** |  | -0,17 | 0,07 | 0,04 | 0,07 | -0,01 | -0,03 | -0,09 | -0,02 |
| **quinidine** |  | -0,18 | -0,05 | -0,12 | 0,11 | 0,21 | 0 | 0,08 | 0,11 |
| **clorgiline** |  | -0,19 | -0,08 | 0,04 | -0,01 | -0,07 | 0,08 | 0,11 | 0,01 |
| **kb_nb_142-70** |  | -0,2 | 0,21 | 0,17 | 0,1 | 0,26 | 0,18 | -0,01 | 0,22 |
| **pamoic_acid** |  | -0,21 | 0,07 | 0,02 | -0,02 | -0,02 | -0,04 | 0 | -0,08 |
| **dpp-iv-in-2** |  | -0,22 | 0 | 0,03 | -0,01 | -0,25 | -0,04 | 0,05 | -0,03 |
| **uk_356618** |  | -0,23 | 0,02 | 0,13 | 0,08 | 0,06 | 0,04 | 0,27 | 0,11 |
| **int-747** |  | -0,23 | -0,03 | -0,01 | -0,07 | -0,02 | 0,08 | 0,04 | -0,01 |
| **dy_131** |  | -0,27 | 0,01 | 0,02 | -0,03 | 0 | 0 | -0,09 | 0,03 |
| **h-arg(no2)-oh** |  | -0,27 | 0,08 | 0,03 | -0,01 | -0,04 | 0 | -0,08 | -0,1 |
| **cgs_35066** |  | -0,29 | 0,03 | 0,01 | -0,01 | -0,07 | -0,02 | -0,05 | -0,07 |
| **thiothixene** |  | -0,3 | -0,07 | 0,08 | 0,17 | 0,03 | 0 | 0,04 | 0,05 |
| **ibc_293** |  | -0,33 | 0,11 | 0 | 0,11 | 0,02 | 0,01 | 0,04 | 0,04 |
| **mln4924** |  | -0,36 | 0 | 0,28 | 0,04 | 0,16 | 0,16 | -0,01 | 0,31 |
| **tolimidone** |  | -0,41 | -0,04 | 0,02 | 0,09 | 0,03 | 0,14 | -0,02 | 0,07 |
| **gsk2636771** |  |  | 0,05 | 0,02 | -0,01 | -0,04 | -0,06 | 0,04 | -0,07 |
| **4_4-dimethoxybenzil** |  |  | 0 | -0,06 | -0,08 | -0,07 | -0,04 | -0,09 | -0,06 |
| **2-bromophenethylamine** |  |  | 0,03 | -0,01 | 0,04 | 0,05 | -0,01 | -0,01 | 0,02 |
| **a_967079** |  |  | -0,02 | -0,07 | -0,02 | 0,08 | -0,01 | -0,1 | 0,01 |
| **atomoxetine** |  |  | 0,03 | 0,02 | 0,02 | 0,01 | 0,03 | -0,06 | 0 |
| **cetirizine** |  |  | 0,1 | -0,07 | -0,1 | 0,01 | 0,01 | -0,16 | -0,09 |
| **6-aminochrysene** |  |  | -0,04 | 0,04 | 0,11 | 0,05 | 0,04 | 0,01 | 0,09 |
| **abl127** |  |  | -0,02 | 0 | -0,05 | -0,01 | 0 | -0,11 | 0 |
| **guanethidine** |  |  | -0,02 | 0,01 | -0,03 | -0,01 | -0,04 | 0,08 | 0,03 |
| **tnp-470** |  |  | 0,07 | 0,19 | -0,02 | -0,03 | 0,09 | 0,09 | 0,15 |

**Supplementary Table 2.** Primers used in the study.

| **Primer** | **Sequence 5’ – 3’** | **Reference** |
| --- | --- | --- |
| Genome control_fwd (Primer A) | CCATGTTACCAACATCTTCCTCTCC | (Burov et al. 2021) |
| Control_mCherry_rev (Primer B) | CACCCTTGGTCACCTTCAGCTTGGC | (Burov et al. 2021) |
| Cherry_control_fwd (Primer C) | GCCACTACGACGCTGAGGTCAAGACC | (Burov et al. 2021) |
| Rev_control (Primer D) | GCCCCACCCAACAACCACAGGC | (Burov et al. 2021) |
| qPCR_transgene_fwd (Primer G) | GAAGGAAGATGGTTGGGTGAAAG | (Burov et al. 2021) |
| qPCR_transgene_rev (Primer H) | ACCTTGAAGCGCATGAACTCC | (Burov et al. 2021) |
| PSMB5_qPCR_fwd | CTCCAAACTGCTTGCCAAC | (Morozov et al. 2019) |
| PSMB5_qPCR_ rev | GTTCCCTTCACTGTCCACG | (Morozov et al. 2019) |
| PSMB9_qPCR_fwd | GCTGCTGATGCCCAAGC | (Morozov et al. 2019) |
| PSMB9_qPCR_ rev | GCTGATATTTCTCACCACATTTGC | (Morozov et al. 2019) |
| PSMB10_qPCR_fwd | GGTTCCAGCCGAACATGA | (Morozov et al. 2019) |
| PSMB10_qPCR_ rev | ATGCGTCCACATTGCCC | (Morozov et al. 2019) |
| CD274_qPCR_fwd | CTATTGGGAAATGGAGGATAAG |  |
| CD274_qPCR_rev | GTTCAGAGGTGACTGGAT |  |
| Actin_b_fwd | TTGGCAATGAGCGGTTCC | (Morozov et al. 2019) |
| Actin_b_rev | GAGTTGAAGGTAGTTTCGTGG | (Morozov et al. 2019) |

**Supplementary references**

1. Burov, A.; Funikov, S.; Vagapova, E.; Dalina, A.; Rezvykh, A.; Shyrokova, E.; et al. A Cell-Based Platform for the Investigation of Immunoproteasome Subunit β5i Expression and Biology of β5i-Containing Proteasomes. Cells. 2021, 10(11), 3049. DOI: 10.3390/cells10113049
2. Mitchell, D.C.; Kuljanin, M.; Li, J.; Van Vranken, J.G.; Bulloch, N.; Schweppe, D.K.; et al. A proteome-wide atlas of drug mechanism of action. Nat Biotechnol. 2023, 41(6), 845-857. DOI: 10.1038/s41587-022-01539-0
3. Morozov, A.V.; Burov, A.V.; Astakhova, T.M.; Spasskaya, D.S.; Margulis, B.A.; Karpov, V.L. Dynamics of the Functional Activity and Expression of Proteasome Subunits during Cellular Adaptation to Heat Shock. Mol Biol (Mosk). 2019, 53(4), 638-647. DOI: 10.1134/S0026898419040086
